# Enhanced Solubility and Deprotection of Pyrene-4,5,9,10-tetraones through Propylene Glycol and Propanediol Protection

**DOI:** 10.1021/acs.orglett.5c03752

**Published:** 2025-10-10

**Authors:** Robin Wessling, Kylie Chinner, Paula Wenz, Clara Douglas, Oliver Dumele, Birgit Esser

**Affiliations:** † Institute of Organic Chemistry II and Advanced Materials, 9189 Ulm University, Albert-Einstein-Allee 11, 89081 Ulm, Germany; ‡ Institute of Organic Chemistry, 9174 University of Freiburg, Albertstr. 21, 79104 Freiburg, Germany

## Abstract

Pyrene-4,5,9,10-tetraone (**PTO**) is a building block of significant interest for functional organic materials. Due to the sensitivity of the vicinal diones toward bases and metal-ion chelation, and low solubility, **PTO** is typically protected as a ketal with ethylene glycol (EtG). Herein, we report the use of propylene glycol (PrG), propane-1,3-diol (PD) or 2,2-dimethylpropane-1,3-diol (DMPD) for its protection. The PrG-protected **PTO** has a 44-fold increased solubility relative to EtG-protected **PTO** due the introduced regio- and stereoisomerism. Protection with PD and DMPD surprisingly produced “half” protected **PTO**s, with maintained protective capabilities, and milder deprotection conditions for PD. All protected **PTO**s underwent successful functionalization reactions with high yields. Due to its stereocenter, use of PrG-protecting groups gives entry to protected **PTO**s with chiral-optical properties. This new series of ketal-protecting groups should considerably facilitate the future synthesis of **PTO**-based materials.

## Graphical abstract

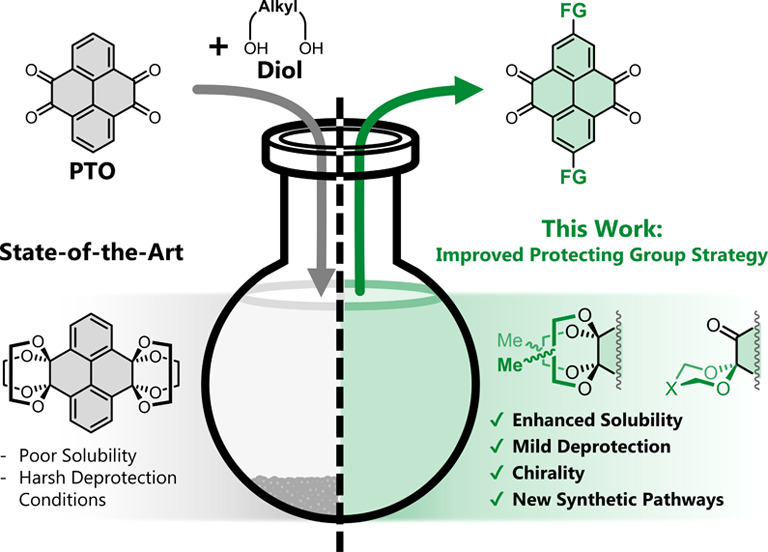

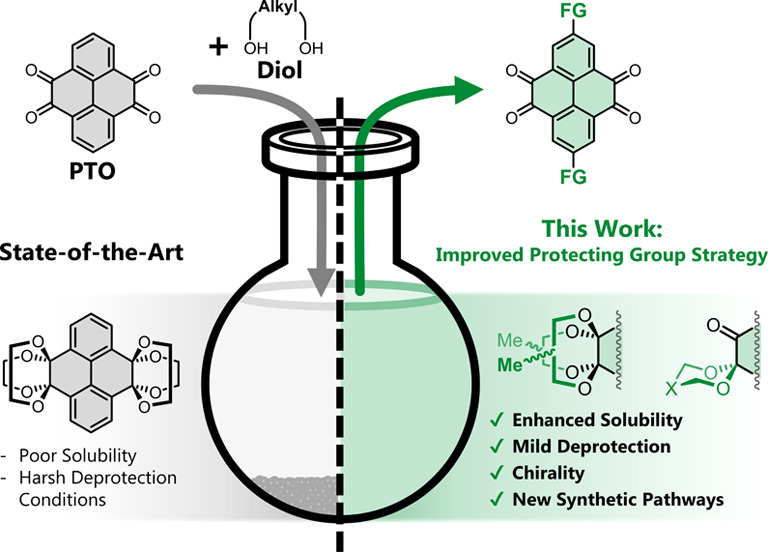

4,5,9,10-Pyrenetetraone (**PTO**, Scheme 1) is a synthetic building block of significant and increasing interest for a large variety of functional organic materials, used in organic batteries,^1^ organic light-emitting diodes,^2^ organic field-effect transistors,^3^ and organic solar cells,^4^ among others. **PTO** is often employed as an intermediate for certain synthetic routes, in particular, pyrene-fused azaacenes,^3,5,6^ covalent organic frameworks,^7^ and nanoribbons.^8,9^ Recently, **PTO** has gained particular interest as a redox-active group for organic energy storage.^1,10–26^ The four carbonyl groups of **PTO** enable a reversible uptake of up to four electrons, while **PTO** possesses a low molecular weight. This leads to a high theoretical specific capacity of 408 mAh g^–1^ for the four electrons in monomeric **PTO**. It has been employed both as a small-molecule battery-electrode-active material and in polymeric form (homopolymer and copolymers).^1,27^ Additionally, **PTO** shows promising results in so-called post-lithium cell technologies, such as sodium,^12^ magnesium,^28^ and aluminum batteries,^23^ due to its geminal diones, which are beneficial for the chelation of cations. However, these diones, in the K-region of the molecule, are also one of its greatest chemical weaknesses. While their reduction is electrochemically highly reversible and can serve as “handles” for ring fusion in many synthetic strategies, the two diones show little chemical resistance when exposed to alkaline conditions. Contact with bases in the presence of trace amounts of oxygen can irreversibly decompose **PTO** (see SI section S2.5). An additional shortcoming is the tendency for cation chelation, which inhibits the use of metal catalysis in many cases during synthesis.^29–31^ These issues, alongside its intrinsic poor solubility in organic solvents, greatly limit the versatility of **PTO** from a synthetic standpoint.

**Scheme 1:**
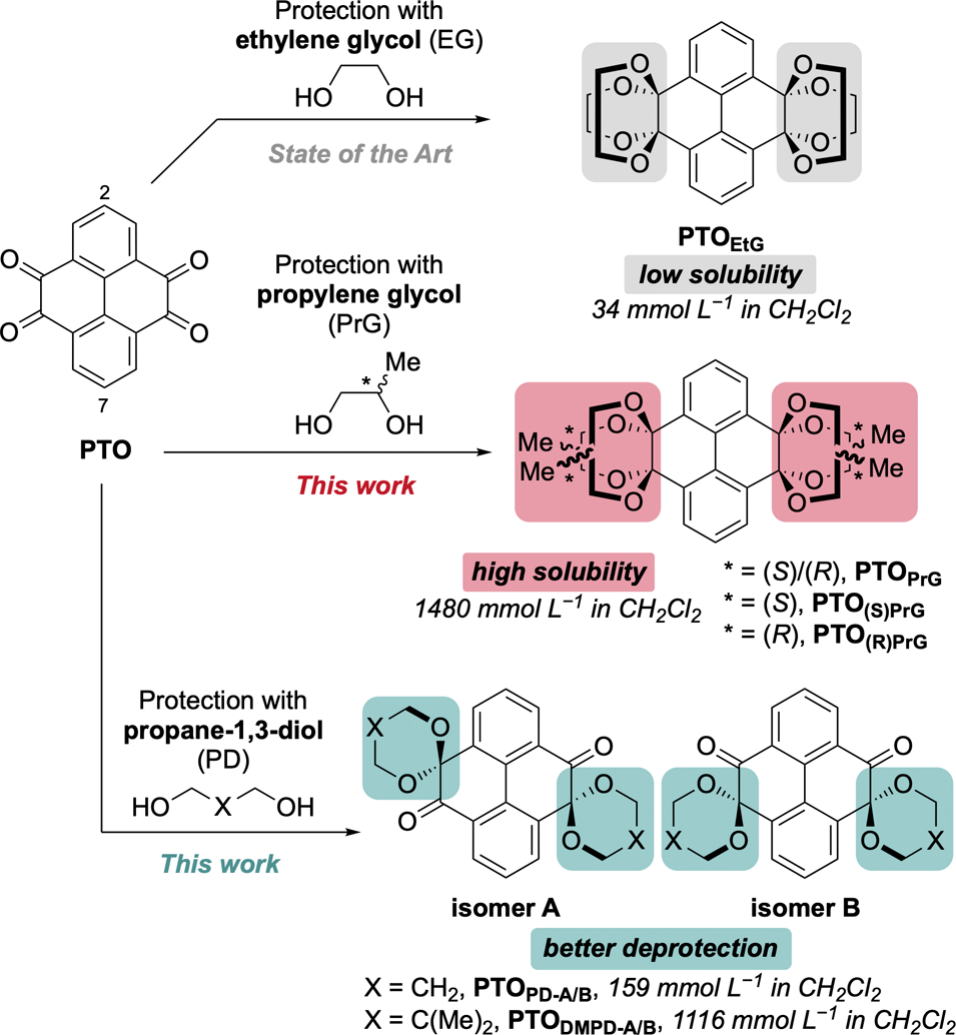
Protection of Pyrenetetraone (**PTO**)

A common strategy to circumvent these issues is the protection of the diones as ketals using ethylene glycol (EtG) (Scheme 1, top). The ketals offer protection against basic decomposition, inhibit cation chelation, and slightly increase solubility.^32^ However, the protection step usually suffers from moderate yields (50–82%)^5,24,30,32–35^ due to the still poor solubility of EtG-protected **PTO** (**PTO**_**EtG**_). This presents a limitation to, for instance, the synthesis of polymers. Additionally, deprotection of **PTO**_**EtG**_ often requires high concentrations of acid (i.e., 90% aq TFA),^10,34,36^ limiting the synthesis of more delicate functional organic materials, such as strained aromatic macrocycles^32^ (SI Figure S34).

To alleviate these issues, which we frequently struggle with in both polymerization reactions and the synthesis of strained **PTO**-containing macrocycles, we set out to investigate new protecting group strategies for **PTO**. We herein report new synthetic pathways to functionalized **PTO** derivatives with the goal of increasing the solubility of intermediates, and enabling subsequent deprotection at milder conditions.^37^ These significantly improve the synthetic versatility of **PTO** and facilitate new applications. The basis of this new approach is the simple and inexpensive exchange of the EtG protecting groups for an alternative diol that is sterically more demanding and introduces regio- and/or stereoisomerism to increase solubility.

For this purpose, we screened a plethora of options (SI Section S7). We found racemic and enantiomerically pure propylene glycol (1,2-propanediol, PrG), propane-1,3-diol (PD), and its derivative 2,2-dimethylpropane-1,3-diol (DMPD) to be the best options (Scheme 1) (price comparison: SI Section S1.1). The extra methyl groups of PrG drastically increase solubility through introduction of regio- and stereoisomerism compared to nonisomeric **PTO**_**EtG**_. Protection with PD and DMPD surprisingly produced “half” protected **PTO**s which maintained the same protective capabilities as “fully” protected **PTO**_**EtG**_ (Scheme 1, bottom). Deprotection at milder conditions is now also possible due to the different nature of the ketal and resulting steric and electronic effects.

First, we investigated PrG as a protecting group and obtained **PTO**_**PrG**_ in an outstanding yield of 96% by acid-catalyzed ketalization (Scheme 2). This yield was reliably >90% in repeated reactions of different batch sizes—a drastic increase in overall atom economy compared to **PTO**_**EtG**_. For **PTO**_**PrG**_, a total of 76 different regio- and stereoisomers theoretically exist (SI Figure S62). The multitude of isomers are evident in the nuclear magnetic resonance (NMR) spectrum of **PTO**_**PrG**_, which appears chaotic in comparison to **PTO**_**EtG**_. The crystal structure of a **PTO**_**EtG**_ analogue, reported by Mateo-Alonso and co-workers, indicates that instead of 1,3-dioxolanes (one protecting group per carbonyl), 1,4-dioxanes (two protecting groups over two carbonyls) are formed during protection.^38^ These 1,4-dioxane rings assume a chair conformation for **PTO**_**EtG**_ and **PTO**_**PrG**_ (SI Figure S4). For **PTO**_**EtG**_, which has four chemically equivalent methylene protons, two broad signals are observed in the ^1^H NMR spectrum, in line with literature reports.^5,32,38^

**Scheme 2:**
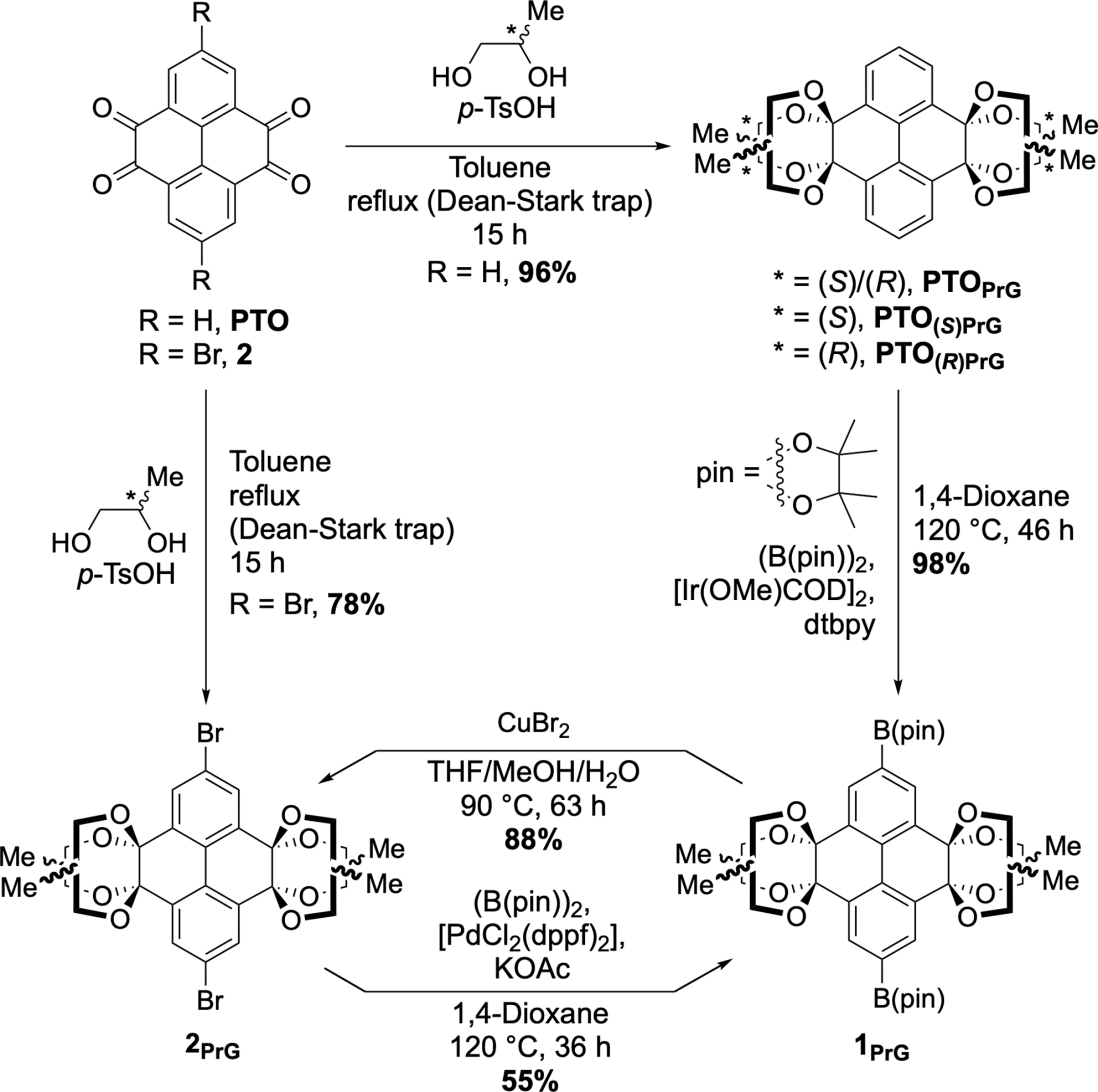
Synthesis and Versatility of Propylene Glycol (PrG)-Protected PTO

The regio- and stereoisomerism introduced with PrG dramatically increase the solubility of **PTO**_**PrG**_. While the solubility of **PTO**_**EtG**_ in dichloromethane amounts to merely 34 mmol L^–1^, **PTO**_**PrG**_ exhibits a solubility of 1480 mmol L^–1^, representing a considerable 44-fold increase. This enhanced solubility significantly facilitates the purification (the removal of undesired side products; the isomers of **PTO_PrG_** are inseparable) of **PTO**_**PrG**_ compared to **PTO**_**EtG**_, allowing for the use of column chromatography.

Implementing enantiomerically pure (*S*)-(+)- or (*R*)-(−)-PrG as the protecting group gave **PTO**_**(** *S* **)PrG**_ and **PTO**_**(** *R* **)PrG**_, respectively, as geometric isomers analogous to *rac*-**PTO**_**PrG**_ (both, >90% yield, Scheme 2). This drastically reduces the isomer complexity from 76 inequivalent isomers to only 7 regioisomers, as all four stereocenters of each regioisomer are homochiral. A calculated energy range of 7 kcal mol^–1^ among the isomers was found via density functional theory (DFT) calculations, indicating that multiple isomers are likely populated (SI Section S10.1). The ^1^H NMR spectra between racemic and homochiral isomer mixtures reflect this reduction in complexity, with a sharpening of signal shape for the homochiral isomer mixtures compared to that for *rac*-**PTO**_**PrG**_ (SI Figure S29).

Electronic circular dichroism (ECD) spectra of the homochiral isomers revealed the transfer of chirality of (*S*)- and (*R*)-PrG to the central pyrene core (SI Figure S58). Intense Cotton effects in the UV range indicate a chiral twisting of the central core. Hence, this motif could be implemented in **PTO**-based chiroptical materials.

As with **PTO**_**EtG**_, functional groups at the 2,7-positions can be introduced before (Br, I)^31^ or after protection (pinacol boronic esters (Bpin)),^5^ with potential follow-up reactions, and a final deprotection of the diones upon treatment with aqueous trifluoroacetic acid (TFA). To test the robustness of the PrG protection, we subjected the protected PTOs to transition-metal-containing reactions at the 2,7-positions, typically employed in the construction of functional organic materials (Scheme 2). The iridium-catalyzed borylation of protected PTO is a feasible method to introduce boronic ester groups, enabling consecutive Suzuki–Miyaura coupling reactions. Borylation of **PTO**_**PrG**_ furnished **1**_**PrG**_, the corresponding 2,7-dipinacol boronic ester, with close to quantitative yield (98%), and the PrG protection groups were fully retained.

For access to dibrominated PrG-protected **PTO** (**2**_**PrG**_) two options are available: transformation of **1**_**PrG**_ via a CuBr_2_-mediated bromodeborylation (88% yield, compared to <30% for less soluble intermediates),^5^ or beginning from 2,7-dibromo-**PTO** (**2**)^38^ and protecting the diones with PrG (78%) (Scheme 2). The bromides of **2**_**PrG**_ can also be converted into boronic esters via Miyaura borylation to obtain **1**_**PrG**_ (55%).

Our second approach was based on propane-1,3-diols as protecting groups (PD and DMPD) due to their symmetric, yet slightly bulkier, structure compared to EtG (Scheme 3).

**Scheme 3:**
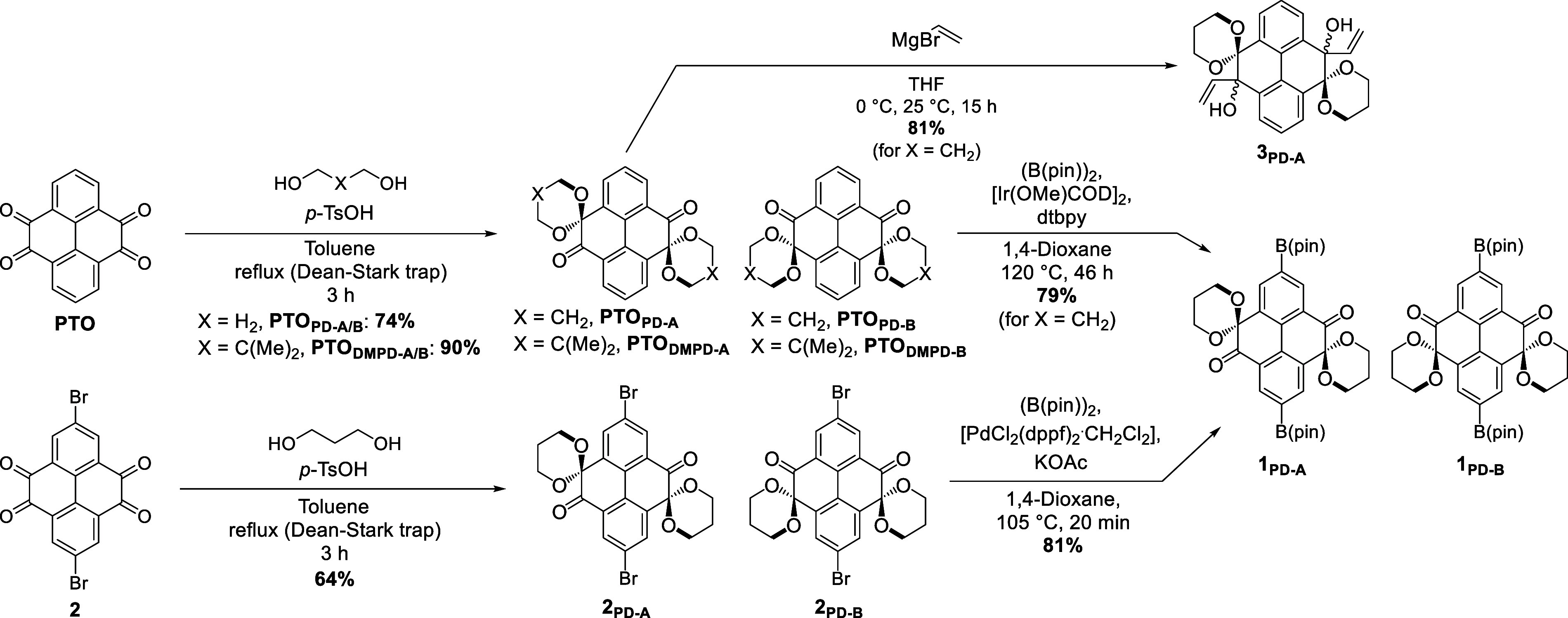
Synthesis and Versatility of Propane-1,3-diol (PD)- and 2,2-Dimethylpropane-1,3-diol (DMPD)-Protected PTOs

Surprisingly, instead of a 4-fold protection of the diones, only a 2-fold protection occurred to give **PTO**_**PD‑A/B**_, with **PTO**_**PD‑A**_ unambiguously confirmed by single-crystal X-ray crystallography (SI Section S8) and **PTO**_**PD‑A/B**_ confirmed by NOESY NMR (SI Figures S17 and S20). Increasing PD from 25 to 250 equiv in the protecting step, or the reaction time from 3 to 24 h, did not result in any conversion to a “fully” protected **PTO**. Interestingly, **PTO**_**PD‑A/B**_ exists as a mixture of two isomers with *anti*-protected ketones (**PTO**_**PD‑A**_) and *syn*-protected ketones (**PTO**_**PD‑B**_) on alternate sides of the molecule. This isomeric mixture is obtained in a 1:1 ratio from the chromatographed isomer mixture (74% yield, determined by ^1^H NMR spectroscopy) and can be separated by recrystallization from hot ethyl acetate to give **PTO**_**PD‑A**_ (42%) and **PTO**_**PD‑B**_ (26%, both isolated yields with regard to starting material of the reaction). The component of dissymmetry between the isomers in their mixture likely contributes to the 5-fold increased solubility of **PTO**_**PD‑A/B**_ (159 mmol L^–1^) in comparison to **PTO**_**EtG**_ (34 mmol L^–1^).

Again, this solubility can be dramatically increased through the introduction of additional methyl groups. Acid-catalyzed ketalization of **PTO** with DMPD gave **PTO**_**DMPD‑A/B**_ in an overall increased yield (90%) and solubility (1116 mmol L^–1^).

Although it only yields “half” protected **PTO**_**PD‑A/B**_, the protection with PD or DMPD is just as effective as that with EtG or PrG. The resulting **PTO** derivatives can successfully undergo transition-metal catalysis while retaining both protecting groups (Scheme 3). The protected 2,7-dipinacol boronic esters **1**_**PD‑A/B**_ can be achieved via iridium-catalyzed borylation of **PTO**_**PD‑A/B**_ (79%) or from palladium-catalyzed Miyaura borylation of dibrominated **2**_**PD‑A/B**_ (68%). Dibrominated **2**_**PD‑A/B**_ was obtained by the PD protection of **2** (64%).

Analysis of crude ^1^H NMR spectra of the borylated products (**1**_**PD‑A/B**_) showed a 1:1 ratio between the A and B isomers (starting from a 1:1 **PTO**_**PD‑A/B**_ isomeric mixture) (SI Figure S33). This indicates that during this transformation there is no chemical preference between isomers.

Further expanding on the dual protection offered by propane diol, we found that the two unprotected ketones in **PTO**_**PD‑A**_ undergo Grignard addition while maintaining their PD protection. Reaction with vinylmagnesium bromide furnished **3**_**PD‑A**_ in 81% yield (Scheme 3). This enables the use of PD-protected PTO moieties as precursors for **PTO**-based materials with unsymmetric functionalization (SI Section S2.2).

To investigate subsequent synthetic possibilities toward **PTO**-containing functional materials, the deprotection of our series of ketal-**PTO**s was investigated to identify the mildest conditions required for full deprotection (Table 1).

**Table 1:**
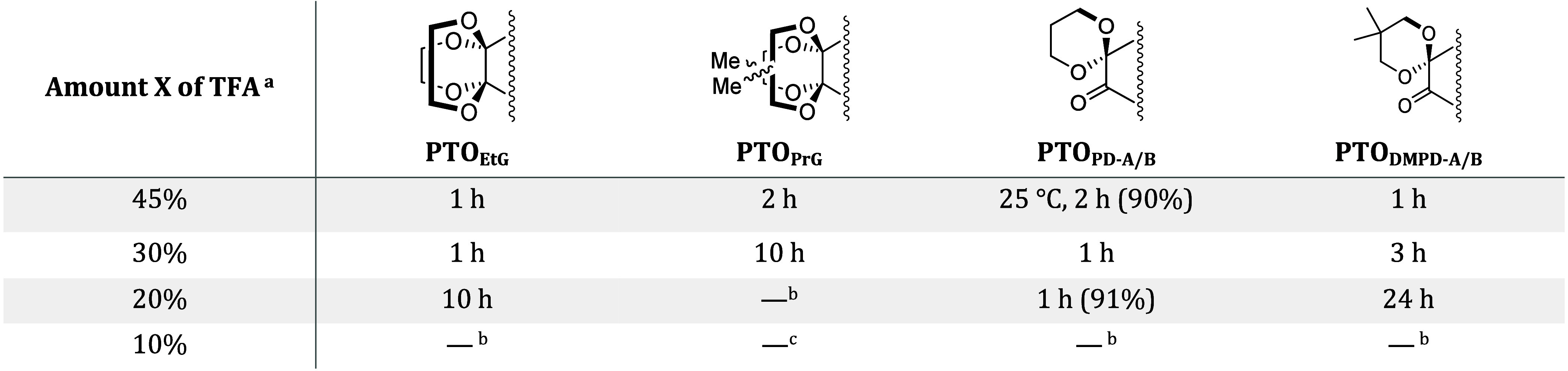
Full Deprotection of Ketal-Protected PTOs^a^

a Deprotections were conducted in TFA/CHCl_3_/H_2_O = X/(90–X)/10 (v/v/v; TFA = trifluoroacetic acid). Deprotections required a temperature of 60 °C for full deprotection, unless otherwise stated. Isolated yields in parentheses.

b Only partial deprotection observed after 48 h.

c No deprotection observed after 48 h.

**PTO**_**PD‑A/B**_ demonstrated the most efficient deprotection, achieving full conversion to **PTO** at 25 °C within 2 h using TFA/CHCl_3_/H_2_O 45:45:10 (v/v/v) with an isolated yield of 90%. Under the same conditions, **PTO**_**EtG**_, **PTO**_**PrG**_, and **PTO**_**DMPD‑A/B**_ achieved only partial deprotection and required heating to 60 °C for full deprotection to **PTO** (SI Figure S31). At the lowest tested ratio (10% TFA) no ketal-**PTO** was fully deprotected, even after 48 h at 60 °C. However, increasing the TFA ratio to 20% allowed **PTO**_**PD‑A/B**_ to be fully deprotected within 1 h at 60 °C (91% yield)—considerably faster than **PTO**_**EtG**_, which required 10 h under identical conditions (both **PTO**_**PrG**_ and **PTO**_**DMPD‑A/B**_ did not fully convert at these conditions). Furthermore, both **PTO**_**PD‑A**_ and **PTO**_**PD‑B**_ exhibited nearly identical rates of deprotection (SI Figure S32), indicating that they can be used as an isomeric mixture without affecting the overall outcome for deprotection.

Interestingly, while an increased solubility is extremely beneficial for synthesis in the construction of **PTO**-based materials (yields, purification, and characterization), the increased solubility had no significant effect on the conditions required to fully deprotect the ketal-**PTO**s, with **PTO**_**PrG**_ and **PTO**_**DMPD‑A/B**_ performing slightly worse than their less soluble counterparts. These findings suggest that a balance between solubility and deprotection efficiency is necessary with PD emerging as the optimal protecting group for **PTO** within this context.

In conclusion, we present new protecting-group strategies for **PTO**. Using PrG or PD derivatives instead of the state-of-the-art EtG furnishes a significantly higher solubility of protected **PTO**_**PrG**_ (44-fold increase), **PTO**_**PD‑A/B**_ (5-fold), and **PTO**_**DMPD‑A/B**_ (33-fold) compared to **PTO**_**EtG**_. The new series of protected **PTO**s all undergo successful functionalization reactions with higher yields and in simpler reaction setups compared to **PTO**_**EtG**_ due to increased solubility. Subsequent deprotection of **PTO**_**PD‑A/B**_ (60 °C, 1 h, 91% yield) could be performed under milder conditions (20% TFA) compared to **PTO**_**EtG**_ (60 °C, 10 h). Thus, demonstrating a clear advantage in using PrG as a protecting group for increased solubility and overall yields during postprotected functionalizations, as well as full protection of all ketones, and PD as a protecting group for subsequent deprotection under milder conditions. These results should greatly facilitate the synthesis of future **PTO**-based materials, enabling the use of **PTO** in a broader range of synthetic and materials contexts.

## Supplementary Material



## Data Availability

The data underlying this study are available in the published article, in its Supporting Information, and openly available in Zenodo https://zenodo.org/doi/10.5281/zenodo.15211105.

## Notes

Materials and methods, synthetic manipulations, information on the regioisomers, NMR spectra, mass spectra, single-crystal X-ray data, IR, UV–vis and ECD spectroscopy, computational methods (PDF)

## ⊥

Department for Energy Conversion and Storage, Technical University of Denmark (DTU), Anker Enge-lunds Vej 301, 2800 Kongens Lyngby, Denmark

## ¶

Institute of Organic Chemistry, University of Cologne, Greinstrasse 4, 50939 Cologne, Germany.
