# Supplementary material for: Enhanced Solubility and Deprotection of Pyrene-4,5,9,10-tetraones through Propylene Glycol and Propanediol Protection
Source: Org Lett. 2025 Oct 10;27(43):12047–52. doi: 10.1021/acs.orglett.5c03752 (PMC12584142; doi:10.1021/acs.orglett.5c03752)
Supplement: Supplementary file 1 [file ol5c03752_si_001.pdf]

Supplementary Information for

Enhanced Solubility and Deprotection of Pyrene-4,5,9,10-tetraones through Propylene Glycol and Propane Diol Protection

Robin Wessling,[§,a,b] Kylie Chinner,[§,b] Paula Wenz,[a] Clara Douglas,[b] Oliver Dumele,[b] and Birgit Esser[a*]*

[a]Institute of Organic Chemistry II and Advanced Materials, Ulm University, Albert-Einstein-Allee 11, 89081 Ulm, Germany; [b]Institute of Organic Chemistry, University of Freiburg, 79104, Freiburg, Germany.

Table of Contents

S1. Materials and Methods S1
S1.1. Price Comparison of Protecting Groups S3
S2. Synthetic Procedures S4
S2.1. Propylene Glycol (PrG) Protecting Groups S4
S2.2. Propane-1,3-diol (PD) Protecting Groups S9
S2.3. Dimethylpropane-1,3-diol (DMPD) Protecting Groups S16
S2.4. General Procedure for the Deprotection of Protected PTOs S17
S2.5. Decomposition Experiments S18
S3. Structural Analysis of PTO_{PrG} S21
S4. Selected NMR Spectra S23
S5. Mass Spectra S38
S6. Infrared Spectroscopy S41
S7. Attempts Towards Alternative PTO Protecting Groups S45
S8. Single-Crystal X-Ray Data S50
S9. UV-Vis and ECD Spectroscopy S54
S10. Computational Methods S58
S10.1. Geometry Optimized Structures of PTO_{PrG} Isomers S58
S10.2. Atomic Coordinates of Calculated Structures S60
S11. References S66
S12. Author Contributions S67

S1. Materials and Methods

Commercially Available Chemicals: Fine chemicals and solvents were purchased from ABCR, ACROS-ORGANICS, ALFA-AESAR, BLDPHARM, CHEMPUR, ROTH, SIGMA-ALDRICH / MERCK or TCI and used directly without further purification unless otherwise noted. Solvents were used as purchased in analytical or HPLC grade. Solvents denoted as "degassed" were degassed by using three cycles of freeze-pump-thaw procedure.

Inert Working Procedures: Moisture- or oxygen-sensitive reactions were carried out in glassware that was previously dried by heating under vacuum (< 10^{-2} mbar), and standard SCHLENK techniques were applied using dry argon (Argon 4.6 by MTI INDUSTRIEGASE). Anhydrous 1,4-dioxane was purchased from ACROS-ORGANICS (extra dry, < 50 ppm H_2O, AcroSeal™) and further stored over activated molecular sieves (3 Å).

Flash column chromatography was carried out using silica gel 60, grain size 40–63 µm (230–400 mesh) from MACHEREY-NAGEL.

Medium pressure liquid chromatography (MPLC) was carried out using a BIOTAGE Isolera One System or a TELEDYNE CombiFlash R_f+. Prepacked CHROMABOND Flash BT 25 SiOH (silica gel, 25 g) or TELEDYNE (silver) cartridges were utilized.

Analytical thin layer chromatography was carried out using silica gel-coated aluminum plates with a fluorescence indicator (MERCK *60 F254* or MACHEREY-NAGEL *ALUGRAM Xtra SIL G/UV254*). Detection was carried out by using UV light of two different wavelengths (λ_{max} = 254 or 365 nm).

Nuclear magnetic resonance (NMR) spectra were recorded at 298 K, unless otherwise noted, on the following spectrometers: BRUKER *Avance 400 neo* [400.1 MHz (^{1}H), 100.6 MHz (^{13}C)], BRUKER *Avance III HD 500* [500.3 MHz (^{1}H), 125.8 MHz (^{13}C)], Chemical shifts are reported in parts per million (ppm, δ scale) relative to the signal of tetramethylsilane (δ = 0.00 ppm). ^{1}H NMR spectra are referenced to tetramethylsilane as an internal standard or the residual proton signal of $CDCl_3$: δ = 7.26 ppm. ^{13}C NMR spectra are referenced to $CDCl_3$: δ = 77.2 ppm.[1] Analysis followed first order, and the following abbreviations for multiplets are used: broad (br), singlet (s), doublet (d), triplet (t), quartet (q), multiplet (m) and combinations thereof, *i.e.* doublet of doublets (dd).

Due to the chaotic appearance of the spectrum of the isomeric mixture, we report the integrals of the aliphatic multiplets in percentages of the whole integral of each respective group, to allow reproducibility. Further, the methyl groups of the pinacolato boronic ester could not be unambiguously assigned and were thus integrated together with the methyl groups of the protective groups. Structural assignments were made with additional information from gNOESY, gCOSY and gHSQC experiments.

High resolution mass spectra (HRMS) were measured on a THERMO FISHER SCIENTIFIC *Exactive* mass spectrometer with an orbitrap analyzer with electrospray ionization (ESI) or on a BRUKER *solariX Hybrid 7T FT-ICR* with atmospheric pressure chemical ionization (APCI).

Solubility tests were conducted by preparing a saturated solution of the respective substance in CH_2Cl_2 by successively adding substance to CH_2Cl_2 and repeatedly subjecting the suspension to ultrasound sonication until further dissolution was no longer apparent. Subsequently, the suspension was filtered through a syringe filter (13 mm, 0.45 µm PTFE). An exact amount of the filtrate was then separated off and the solvent was removed under reduced pressure before drying the resulting mass under high vacuum ($<10^{-2}$ mbar) at 60 °C over night. The mass of the residue was determined by subtracting the mass of the empty vial. This procedure was done twice for each substance to minimize variation and the results were averaged.

X-ray data were collected on a BRUKER APEX-II CCD diffractometer. The crystals were kept at 100.00 K during data collection. Using Olex2,[1] the structure was solved with the XT[2] structure solution program using Intrinsic Phasing and refined with the XL[3] refinement package using Least Squares minimization.

Ultraviolet–visible absorbance spectroscopy (UV-Vis) was performed on Agilent Cary 50 instruments connected to a cryostat from UNISOKU SCIENTIFIC INSTRUMENTS (temperature accuracy ±0.1 K) in 10 × 10 mm quartz cuvettes with 3 mL volume. Weighing of small quantities was performed on a Sartorius ME5 analytical microbalance.

Electron circular dichroism (ECD) spectra were recorded in a 1.00 mm path length cuvette using a JASCO J-1500 spectrometer. Wavelengths are reported in nanometers

(nm). The spectra were measured between 250–600 nm, data points were collected in 0.1 nm interval steps.

Melting points (MP) were measured on using a Schorpp Gerätetechnik MPM-HV2 melting point apparatus with the samples finely powdered and loaded into capillary tubes. Compounds which decomposed before melting are indicated with a trailing d at the temperature at which the sample darkened.

Fourier transform infrared (FT-IR) spectra were obtained from 4000 to 500 cm^{-1} using a PerkinElmer Spectrum Two FT-IR spectrometer or an ALPHA II FT-IR spectrometer by BRUKER using ATR (Attenuated Total Reflectance) technique. Absorption bands are reported in cm^{-1}.

S1.1. Price Comparison of Protecting Groups

While ethylene glycol is the most inexpensive reagent, the other diols used in this study are quite inexpensive as well, especially when compared to other common protecting groups such as di-tert-butyl dicarbonate and trimethylsilyl chloride (prices as of 18.06.2025).

Ethylene glycol, ≥99%: 11.57 €/L (Fischer Scientific)

Racemic propylene glycol, ≥99.5%: 27.13 €/L (Sigma-Aldrich)

(R)-(-)-1,2-Propanediol, ee: 98% (GLC), ≥96%: 246.81 €/L (Th. Geyer)

(S)-(+)-1,2-Propanediol, ee: 95% (GLC), ≥96%: 165.89 €/L (Th. Geyer)

Propane-1,3-diol, ≥98%: 57.92 €/L (Th. Geyer)

2,2-Dimethylpropane-1,3-diol, ≥95%: 25.29 €/L (Th. Geyer)

Di-tert-butyl dicarbonate, ≥97%: 190.10 €/kg (ABCR)

Trimethylsilyl chloride, ≥98% (GC): 63.80 €/kg (Sigma-Aldrich)

S2. Synthetic Procedures

S2.1. Propylene Glycol (PrG) Protecting Groups

Syntheses of pyrene-4,5,9,10-tetraone (**PTO**),[4] **2**,[5] and pyrene-4,5,9,10-tetra(ethylene-glycol)ketal (**PTO$_{EtG}$**)[6,7] were conducted according to literature procedures.

Pyrene-4,5,9,10-tetra(propyleneglycol)ketal (PTO$_{PrG}$)

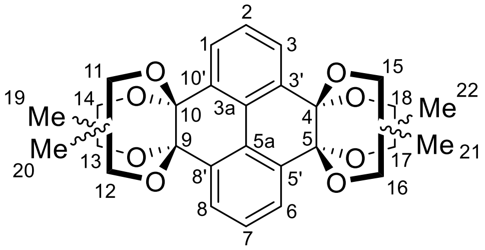

PTO$_{PrG}$

PTO$_{PrG}$ was synthesized by following a modified procedure that was earlier reported for EtG protections by MERZ *et al.*[6] Pyrene-4,5,9,10-tetraone (**PTO**, 1.00 g, 3.81 mmol), *p*-toluenesulfonic acid hydrate (653 mg, 3.43 mmol, 0.90 equiv.) and propane-1,2-diol (69 mL, 73 g, 0.95 mmol, 250 equiv.) were added to toluene (150 mL). The mixture was heated in an oil bath at 125 °C for 15 h using a DEAN-STARK apparatus. After cooling to room temperature, the reaction mixture was washed with H_2O (2 × 100 mL) and brine (100 mL). The combined aqueous phase was extracted with toluene (2 × 75 mL). The combined organic phase was dried over Na_2SO_4, the volatiles were removed under reduced pressure and the crude product was purified using flash column chromatography (SiO_2, *n*-hexane/ethyl acetate 3:1). The title compound (1.81 g, 3.66 mmol, 96%) was obtained as a yellow solid.[1]

***R*$_f$**: 0.40 (SiO_2; *n*-hexane/ethyl acetate 3:1). **^{1}H NMR** (500 MHz, $CDCl_3$, 25 °C): δ = 7.77 – 7.73 (m, 4H, 4 H–C(1,3,6,8)), 7.50 – 7.45 (m, 2H, 2 H–C(2,7)), 4.68 – 4.54 (m, 2H, 2 H–C(12,15)), 4.08 – 3.91 (m, 4H, 2 H–C(14,17), 2 equatorial H_2–C(13,18)), 3.90 – 3.83 (m, 2H, 2 axial H_2–C(11,16), 3.58 – 3.47 (m, 2 axial H_2–C(11,16)), 3.40 – 3.31 (m, 2H, 2 equatorial H_2–C(13,18)), 1.28 – 1.24 (m, 6H, 2 equatorial H_3–C(19,22)), 0.98 – 0.91 (m, 6H, 2 axial H_3–C(20,21)) ppm. **^{13}C NMR** (126 MHz, $CDCl_3$, 25 °C):

[1] The yield for the largest batch size (5.00 mmol **PTO**) was 86%.

δ = 133.8 (C(10′)), 133.5 (C(8′)), 133.3 (C(3′)), 133.1 (C(5′)), 129.6 (C(2,7)), 127.1 (C(1)), 127.04 (C(8)), 126.99 (C(3′)), 126.95 (C(5′)), 93.5 (C(3a)), 93.4 (C(5a)), 92.12 (C(10)), 92.05 (C(5)), 91.53 (C(9)), 91.47 (C(4)), 69.31 (C(11)), 69.26 (C(16)), 68.7 (C(13)), 68.6 (C(18)), 64.71 (C(14)), 64.66 (C(17)), 64.35 (C(12)), 64.27 (C(15)), 16.5 (C(19,22)), 16.0 (C(20,21)) ppm. **HRMS** (pos. ESI): *m/z*: $[M + H]^+$ calcd. for $C_{28}H_{31}O_8^+$ 495.2013; found 495.2018. **MP**: 175.7 – 176.2 °C. **FT-IR (ATR)**: ṽ = 2974 (C–H), 2929 (C–H), 2868 (C–H), 1071 (C=C) cm^{-1}.

2,7-Bis(4,4,5,5-tetramethyl-1,3,2-dioxaborolan-2-yl)-4,5,9,10-tetra (propyleneglycol)ketal-pyrene (1_{PrG})

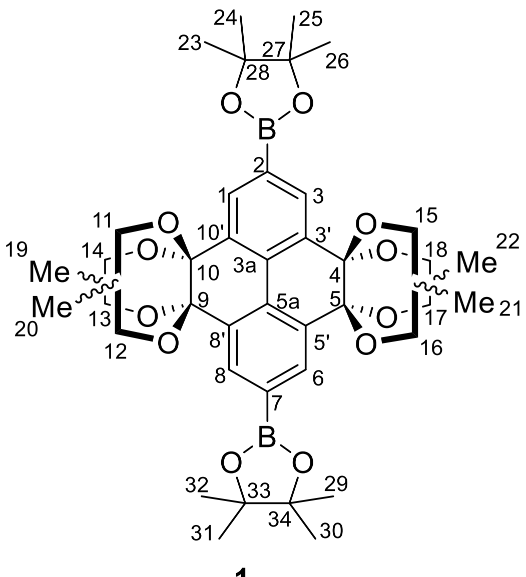

$\mathbf{1_{PrG}}$

Method A (HARTWIG Borylation of $\mathbf{PTO_{PrG}}$):

$\mathbf{1_{PrG}}$ was synthesized following a modified procedure by MERZ *et al.*[6] $\mathbf{PTO_{PrG}}$ (99.1 mg, 200 µmol), bis(pinacolato)diboron (204 mg, 800 µmol, 4.00 equiv.), $[Ir(OMe)COD]_2$ (14.0 mg, 21.1 µmol, 10.5 mol-%) and 4,4′-di-tert-butyl-2,2′-dipyridyl (11.6 mg, 43.2 µmol, 21.6 mol-%) were added to a Schlenk tube and dissolved in anhydrous and degassed 1,4-dioxane (2.0 mL). The closed vessel was heated at 120 °C in an oil for 46 h. After cooling to room temperature, the reaction mixture was washed with H_2O (2 × 20 mL) and brine (20 mL). The combined aqueous phase was extracted with CH_2Cl_2 (4 × 20 mL), and the combined organic phase was dried over $MgSO_4$. After the volatiles were removed under reduced pressure, the crude product was purified by

column chromatography (SiO_2, cyclohexane/CH_2Cl_2/ethyl acetate 4:2:1). The title compound (146 mg, 195 µmol, 98 %) was obtained as a brown solid.[2]

Method B (MIYAURA Borylation of **2_{PrG}**):

1_{PrG} was synthesized following a modified procedure by YAO *et al.*[8] **2_{PrG}** (100 mg, 153 µmol), bis(pinacolato)diboron (85.6 mg, 337 µmol, 2.20 equiv.), [Pd(dppf)Cl_2] (25.0 mg, 30.7 µmol, 20.0 mol-%) and potassium acetate (90.3 mg, 920 µmol, 6.00 equiv.) were added to a Schlenk tube and dissolved in anhydrous and degassed 1,4-dioxane (10 mL). The closed vessel was heated at 120 °C in an oil bath for 44 h. After cooling to room temperature, the reaction mixture was washed with H_2O (20 mL). The aqueous phase was extracted with CH_2Cl_2 (3 × 20 mL). The combined organic phase was washed with brine (20 mL) and dried over Na_2SO_4. The solvent was removed under reduced pressure. The crude product was purified using flash column chromatography (SiO_2, *n*-hexane/ethyl acetate (2:1 to 1:1, v:v)). The title compound (62.6 mg, 83.9 µmol, 55%) was obtained as a dark yellow solid.

R_f: 0.36 (SiO_2; cyclohexane/CH_2Cl_2/ethyl acetate 4:2:1). **^{1}H NMR** (500 MHz, $CDCl_3$, 25 °C): δ = 8.23 – 8.17 (m, 4H, 4 H–C(1,3,6,8)), 4.68 – 4.54 (m, 2H, 2 H–C(12,15)), 4.08 – 3.95 (m, 4H, 2 H–C(14,17), 2 equatorial H_2–C(13,18)), 3.94 – 3.86 (m, 2H, 2 axial H_2–C(11,16)), 3.62 – 3.50 (m, 2 equatorial H_2–C(11,16)), 3.40 – 3.26 (m, 2H, 2 axial H_2–C(13,18)), 1.35 – 1.30 (m, 24H, H_3–C(23,24,25,26,29,30,31,32)), 1.30 – 1.27 (m, 6H, 2 equatorial H_3–C(19,22)), 0.95 – 0.88 (m, 6H, 2 axial H_3–C(20,21)) ppm. **^{13}C NMR** (126 MHz, $CDCl_3$, 25 °C): *δ* = 133.4 (C(1,8)), 133.3 (C(3,6)), 132.7 (C(10′,8′)), 132.5 (C(3′,5′), 116.8 (C(2,7)), 93.5 (C(3a,5a)), 92.9 (C(10,9)), 91.6 (C(4,5)), 83.9 (C(28,33)), 83.7 (C(27,34)), 69.4 (C(11,16)), 68.6 (C(14,17)), 64.6 (C(13,18)), 64.3 (C(12,15)), 25.3 (C(23,32)), 25.2 (24,31), 24.7 (C(25,30)), 24.4 (C(26,29)), 16.5 (C(19,22)), 16.0 (C(20,21)) ppm. **HRMS** (pos. ESI): *m/z*: $[M + H]^+$ calcd. for $C_{40}H_{53}O_{12}B_2^+$ 747.3718; found 747.3723. **MP**: 203 °C d. **FT-IR (ATR)**: ṽ = 2975 (C–H), 2929 (C–H), 1075 (C=C) cm^{-1}.

[2] The yield for the largest batch size (4.85 mmol **PTO_{PrG}**) was 82%.

2,7-Dibromo-4,5,9,10-tetra(propyleneglycol)ketal-pyrene ($\mathbf{2_{PrG}}$)

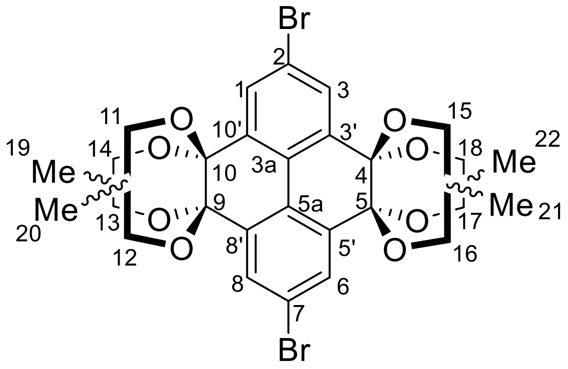

$\mathbf{2_{PrG}}$

Method A (Bromodeborylation of $\mathbf{1_{PrG}}$):

$\mathbf{2_{PrG}}$ was synthesized following a modified procedure by MERZ *et al.*[6] **1** (100 mg, 134 µmol) was dissolved in tetrahydrofuran (1.8 mL), and methanol (1.4 mL) was added. After the mixture was heated to 90 °C in an oil bath, a solution of $CuBr_2$ (179 mg, 804 µmol, 6.00 equiv.) in H_2O (1.4 mL) was added under stirring. The resulting mixture was heated 90 °C for 66 h. After cooling to room temperature CH_2Cl_2 (10 mL) was added, and the mixture was washed with H_2O (2 × 15 mL) and brine (20 mL). The combined aqueous phase was extracted with CH_2Cl_2 (3 × 15 mL) and dried over Na_2SO_4. Removal of the volatiles under reduced pressure and flash column chromatography (SiO_2, *n*-hexane/ethyl acetate 9:1) yielded the title compound (77.1 mg, 118 mmol, 88%) as a pale-yellow powder.

Method B (Acetal Protection of **2**):

$\mathbf{2_{PrG}}$ was synthesized following a modified procedure by MERZ *et al.*[6] **2** (400 mg, 0.95 mmol), *p*-toluenesulfonic acid (179 mg, 0.62 mmol, 0.90 equiv.) and propane-1,2-diol (19 mL, 20 g, 0.26 mmol, 250 equiv.) were added to toluene (75 mL). The mixture was heated in an oil bath at 125 °C for 17 h using a DEAN-STARK apparatus. After cooling to room temperature, the reaction mixture was washed with H_2O (50 mL) and brine (50 mL). The combined organic phase was dried over Na_2SO_4, the volatiles were removed under reduced pressure and the crude product was purified using flash column chromatography (SiO_2, *n*-hexane/ethyl acetate 4:1). The title compound (482 mg, 0.74 mmol, 78%) was obtained as a yellow solid.

***R*f**: 0.37 (SiO_2; *n*-hexane/ethyl acetate 9:1). **^{1}H NMR** (400 MHz, $CDCl_3$, 25 °C, sample contains traces of H_2O with s at δ = 1.55): δ = 7.87 − 7.85 (m, 4H, H–C(1,3,6,8)), 4.66 − 4.52 (m, 2H, 2 H–C(12,15)), 4.06 − 3.93 (m, 4H, 2 H–C(14,17), 2 equatorial H_2–C(13,18)), 3.92 − 3.86 (m, 2H, 2 axial H_2–C(11,16)), 3.54 − 3.42 (m, 2 equatorial H_2–C(11, 16)), 3.39 − 3.33 (m, 2H, 2 axial H_2–C(13,18)), 1.28 − 1.27 (m, 6H, 2 equatorial H_3–C(19,22)), 0.99 − 0.93 (m, 6H, 2 axial H_3–C(20,21)) ppm. **^{13}C NMR** (100 MHz, $CDCl_3$, 25 °C): δ = 135.8 (C(3a,5a)), 135.6 (C(3a,5a)), 135.3 (C(3a,5a)), 135.1 (C(3a,5a)), 130.4 (C(1,3,6,8)), 130.2 (C(1,3,6,8)), 127.6 (C(3′,5′,8′,10′)), 127.3 (C(3′,5′,8′,10′)), 124.1 (C(2,7)), 124.0 (C(2,7)), 123.9 (C(2,7)), 123.8 (C(2,7)), 92.8 (C(4,5,9,10)), 92.1 (C(4,5,9,10)), 91.4 (C(4,5,9,10)), 90.8(C(4,5,9,10)), 69.3 (C(11,12,13,14,15,16,17,18)), 68.9(C(11,12,13,14,15,16,17,18)), 68.9(C(11,12,13, 14,15,16,17,18)), 64.6(C(11,12,13,14,15,16,17,18)), 16.5 (C(19,20,21,22)), 15.9 (C(19,20,21,22)) ppm. **HRMS** (pos. APCI): *m/z*: [*M* + H]$^+$ calcd. for $C_{28}H_{29}{}^{79}Br^{81}BrO_8^+$ 653.0209; found 653.0203. **MP**: 214.4 °C d. **FT-IR (ATR)**: ṽ = 2973 (C–H), 2933 (C–H), 2875 (C–H), 1590, 1431, 1379, 1313, 1288, 1236, 1190, 1161, 1106, 1058, 1006 (C=C) cm^{-1}.

S2.2. Propane-1,3-diol (PD) Protecting Groups

Pyrene-bis(propane-1,3-diol)ketal (PTO$_{PD-A/B}$)

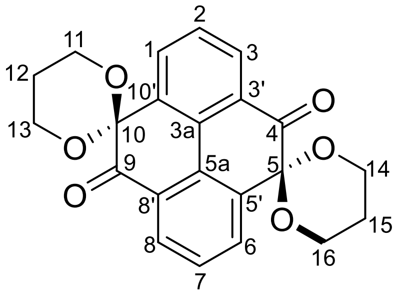

PTO$_{PD-A}$

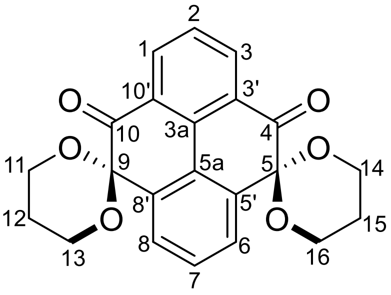

PTO$_{PD-B}$

PTO (500 mg, 1.91 mmol, 1.00 equiv.), *p*-toluenesulfonic acid (326 mg, 1.72 mmol, 0.9 equiv.) and propane-1,3-diol (2.76 mL, 38.1 mmol, 20.0 equiv.) were dissolved in toluene (64 mL, 0.03 M). The mixture was refluxed for 3 h in an oil bath using a DEAN-STARK apparatus. After cooling to room temperature, the reaction mixture was washed with H_2O (2 × 50 mL) and brine (50 mL). The combined aqueous phases were extracted with toluene (3 × 50 mL). The combined organic phases were dried over $MgSO_4$, the volatiles removed under reduced pressure, and the crude product was purified by preparative MPLC (SiO_2, 40 g, gradient cyclohexane/ethyl acetate 10:0 to 8:2). **PTO$_{PD-A/B}$** (530 mg, 1.40 mmol, 74%) as a mixture of two regioisomers was obtained as a yellow solid. Crystallization from hot ethyl acetate (74 mL, 25 °C) by slow evaporation over 3 days separated the isomers. **PTO$_{PD-A}$** crystallizes first as colorless crystalline needles, at the bottom of the crystallization flask, which can be collected by suction with a spatula and with suction using a pipette; then **PTO$_{PD-B}$** as yellow crystalline blocks, stuck at the upper walls of the flask. The crystals were separated, washed with cold ethyl acetate, and dried under vacuum to give **PTO$_{PD-A}$** (302 mg, 0.80 mmol, 42%) and **PTO$_{PD-B}$** (189 mg, 0.50 mmol, 26%).

PTO$_{PD-A}$: ***R*$_f$**: 0.53 (SiO_2; ethyl acetate/cyclohexane 1:1); **^{1}H NMR** (700 MHz, $CDCl_3$, 25 °C, sample contains traces of ethyl acetate with q at δ = 4.12 and s at δ = 2.04 and grease with t at δ = 1.26): *δ* = 8.12 (dd, 2H, H–C(1,6)), 7.96 (dd, 2H H–C(3,8)), 7.46 (t, 2H, H–C(2,7)), 4.55 (td, 4H, H_2–C(13,14)), 4.04 (dd, 4H, C(11,16)), 2.27 (qt, 2H, H_{ax}–C(12,15)), 1.57 (d. septet, 2H, H_{eq}–C(12,15)) ppm; **^{13}C NMR** (175 MHz, $CDCl_3$, 25 °C), assignments based on ^{1}H,^{13}C-HSQC and HMBC NMR spectra): *δ* = 195.6 (C(9,4)), 136.9 (C(5′,10′)*), 133.0 (C(1,6)), 131.1 (C(3a,5a)), 129.55 (C(2,7)), 129.58

(C(3′,8′)*), 128.6 (C(3,8)), 92.4 (C(5,10)), 62.8 (C(11,13,14,16)), 25.2 (C(12,15)) ppm (*interchangeable); **HRMS** (pos. ESI): *m/z*: [*M* + H]+ calcd. for $C_{22}H_{19}O_6^+$ 379.1177; found 379.1176. **MP**: 201.9 – 202.0 °C. **FT-IR (ATR)**: ṽ = 2995 (C–H), 2972 (C–H), 2853 (C–H), 1708 (strong, C=O) cm^{-1}.

$PTO_{PD\text{-}B}$: ***R_f***: 0.53 (SiO_2; ethyl acetate/cyclohexane 1:1); **^1H NMR** (700 MHz, $CDCl_3$, 25 °C, sample contains traces of ethyl acetate with q at δ = 4.12 and s at δ = 2.04 and grease with t at δ = 1.26): *δ* = 8.11 (d, 2H, H–C(1,3)), 7.96 (d, 2H H–C(8,6)), 7.47 (t, 1H, H–C(2)), 7.51 (t, 1H, H–C(7)), 4.54 (td, 4H, H_{ax}–C(11,13,14,16)), 4.04 (dd, 4H, H_{eq}–C(11,13,14,16)), 2.28 (qt, 2H, H_{ax}–C(12,15)), 1.58 (d. septet, 2H, H_{eq}–C(12,15)) ppm; **^{13}C NMR** (175 MHz, $CDCl_3$, 25 °C), assignments based on ^1H,^{13}C-HSQC and HMBC NMR spectra): *δ* = 195.2 (C(4,10)), 137.1 (C(5′,8′)), 135.4 (C(3a)), 132.7 (C(1,3)), 130.5 (C(7)), 129.8 (C(3′,10′)), 129.0 (C(2)), 128.9 (C(6,8)), 125.4 (C(5a)), 92.5 (5,9), 62.8 (C(11,13,14,16)), 25.1 (C(12,15)) ppm; **HRMS** (pos. ESI): *m/z*: [*M* + H]+ calcd. for $C_{22}H_{19}O_6^+$ 379.1177; found 379.1176. **MP**: 201.9 – 202.0 °C. **FT-IR (ATR)**: ṽ = 2995 (C–H), 2972 (C–H), 2853 (C–H), 1708 (strong, C=O) cm^{-1}.

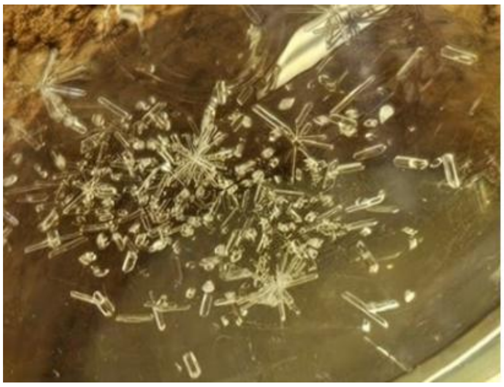

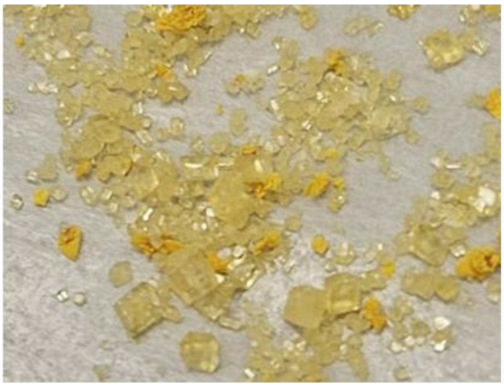

Figure S1. Crystals from recrystallization of **$PTO_{PD\text{-}A}$** (left) and **$PTO_{PD\text{-}B}$** (right).

2,7-Dibromo-bis(propane-1,3-diol)ketal-pyrene (**2**$_{PD-A/B}$)

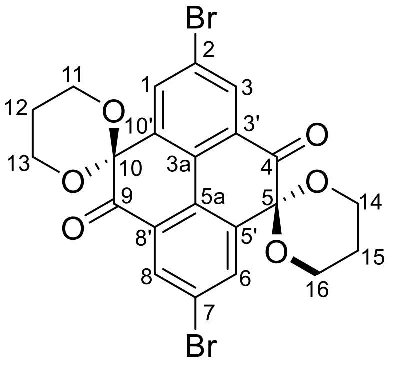

2$_{PD-A}$

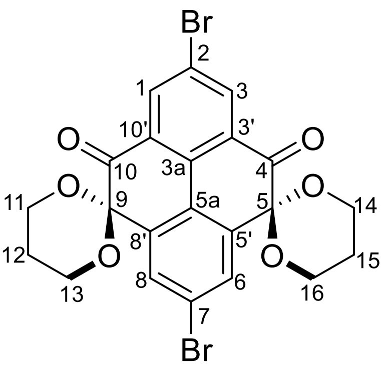

2$_{PD-B}$

2,7-Dibromo-PTO **(2)**[5] (500 mg, 1.19 mmol, 1.00 equiv.), *p*-toluenesulfonic acid (204 mg, 1.07 mmol, 0.9 equiv.) and propane-1,3-diol (1.73 mL, 23.8 mmol, 20.0 equiv.) were dissolved in toluene (64 mL, 0.03 M). The mixture was refluxed for 3 h in an oil bath using a DEAN-STARK apparatus. After cooling to room temperature, the reaction mixture was washed with H_2O (2 × 50 mL) and brine (50 mL). The combined aqueous phases were extracted with toluene (3 × 50 mL). The combined organic phases were dried over $MgSO_4$, the volatiles removed under reduced pressure, and the crude product was purified by preparative MPLC (SiO_2, 40 g, gradient cyclohexane/ethyl acetate 10:0 to 8:2). **2**$_{PD-A/B}$ (410 mg, 0.76 mmol, 64%) as a mixture of two regioisomers was obtained as a yellow solid. Further purification by recrystallization from hot ethyl acetate only achieved partial separation of the two isomers.

2$_{PD-A}$**:** $\boldsymbol{R_f}$: 0.78 (SiO_2; ethyl acetate/cyclohexane 1:1); **^{1}H NMR** (500 MHz, $CDCl_3$, 25 °C, sample contains traces of CH_2Cl_2 with s at δ = 5.30 and H_2O with s at δ = 1.53): δ = 8.21 (d, 2H, H–C(1,6)), 8.06 (d, 2H H–C(3,8)), 4.50 (m, 4H, H_2–C(13,14)), 4.05 (m, 4H, C(11,16)), 2.28 (m, 2H, H_{ax}–C(12,15)), 1.58 (m, 2H, H_{eq}–C(12,15)) ppm; **^{13}C NMR** (125 MHz, $CDCl_3$, 25 °C), assignments based on ^{1}H,^{13}C-HSQC and HMBC NMR spectra): δ = 193.4 (C(9,4)), 138.7 (C(5′,10′)), 136.2 (C(1,6)), 131.1 (C(3a,5a)), 129.55 (C(2,7)), 129.58 (C(3′,8′)), 131.8 (C(3,8)), 91.9 (C(5,10)), 63.0 (C(11,13,14,16)), 25.0 (C(12,15)) ppm; **HRMS** (pos. ESI): *m/z*: $[M + H]^+$ calcd. for $C_{22}H_{17}O_6Br_2^+$ 536.9366; found 536.9377. **MP**: 214.4 °C d. **FT-IR (ATR)**: ṽ = 2965 (C–H), 1697 (strong, C=O) cm^{-1}.

2$_{PD-B}$: ***R*$_f$**: 0.78 (SiO_2; ethyl acetate/cyclohexane 1:1); **^{1}H NMR** (500 MHz, $CDCl_3$, 25 °C, sample contains traces of CH_2Cl_2 with s at δ = 5.30 and H_2O with s at δ = 1.53): *δ* = 8.19 (s, 2H, H–C(1,3)), 8.07 (s, 2H H–C(8,6)), 4.50 (m, 4H, H_{ax}–C(11,13,14,16)), 4.05 (m, 4H, H_{eq}–C(11,13,14,16)), 2.28 (m, 2H, H_{ax}–C(12,15)), 1.58 (m, 2H, H_{eq}–C(12,15)) ppm; **^{13}C NMR** (125 MHz, $CDCl_3$, 25 °C), assignments based on ^{1}H,^{13}C-HSQC and HMBC NMR spectra): *δ* = 193.0 (C(4,10)), 138.6 (C(5′,8′)), 133.3 (C(3a)), 135.4 (C(1,3)), 130.7 (C(7)), 129.1 (C(3′,10′)), 124.2 (C(2)), 132.5 (C(6,8)), 124.2 (C(5a)), 92.0 (5,9), 63.0 (C(11,13,14,16)), 25.0 (C(12,15)) ppm; **HRMS** (pos. ESI): *m/z*: $[M + H]^+$ calcd. for $C_{22}H_{17}O_6Br_2^+$ 536.9366; found 536.9377. **MP**: 214.4 °C d. **FT-IR (ATR)**: ṽ = 2965 (C–H), 1697 (strong, C=O) cm^{-1}.

2,7-Bis(4,4,5,5-tetramethyl-1,3,2-dioxaborolan-2-yl)-bis(propane-1,3-diol)ketal-pyrene (1$_{PD-A/B}$)

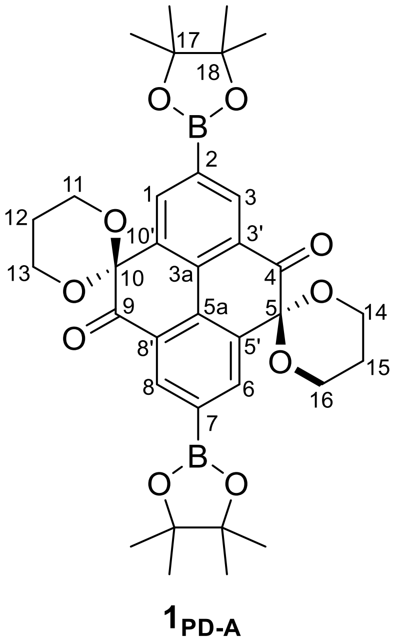

1$_{PD-A}$

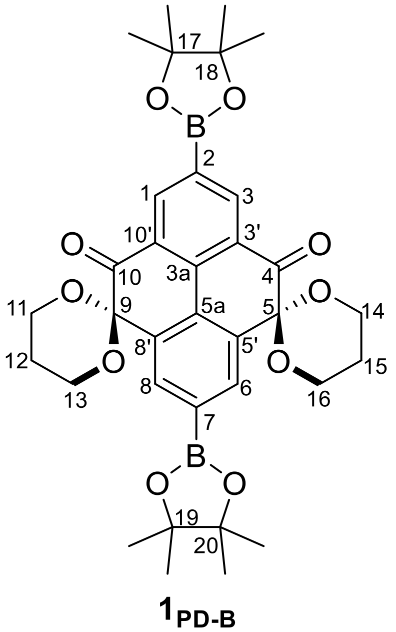

1$_{PD-B}$

<u>Method A (Hartwig Borylation of **PTO$_{PD-A/B}$**):</u>

A solution of **PTO$_{PD-A/B}$** (20.0 mg, 53.0 µmol, 1.00 equiv.) in dry degassed 1,4-dioxane (2.6 mL) was treated with 4,4'-di-*tert*-butyl-2,2'-bipyridine (5.70 mg, 21.0 µmol, 0.40 equiv.), bis(pinacolato)diboron (40.0 mg, 160 µmol, 3.00 equiv.), and $[Ir(OMe)COD]_2$ (7.00 mg, 11.0 µmol, 0.20 equiv.). The reaction mixture was degassed for 10min, until the color changed from dark-brown red to a brighter red color, then stirred at 120 °C in an oil bath for 48 h. After cooling to room temperature, ethyl

acetate was added (10 mL) and the reaction mixture was washed with H_2O (2 × 5 mL) and brine (5 mL). The combined aqueous phases were extracted with ethyl acetate (3 × 5 mL). The combined organic phases were dried over $MgSO_4$, the volatiles removed under reduced pressure, and the crude product was purified by preparative MPLC (SiO_2, 12 g, gradient cyclohexane/ethyl acetate 10:0 to 8:2). **1$_{PD-A/B}$** (26 mg, 41.3 µmol, 79%) as a mixture of two regioisomers was obtained as a cream solid. Further purification by recrystallization from hot ethyl acetate only achieved partial separation of the two isomers.

<u>Method B (MIYAURA Borylation of **2$_{PD-A/B}$**):</u>

A solution of **2$_{PD-A/B}$** (100 mg, 187 µmol, 1.00 equiv.), bis(pinacolato)diboron (118 mg, 466 µmol, 2.50 equiv.), and dry potassium acetate (91.5 mg, 933 µmol, 5.00 equiv.) in dry 1,4-dioxane (6.22 mL) was degassed for 10 min via a constant flow of argon. The solution was treated with [Pd(dppf)Cl_2 × CH_2Cl_2] (15.2 mg, 18.7 µmol, 0.10 equiv.) and stirred at 105 °C in an oil bath for 20 min, during which a change of color to dark brown was observed. After cooling to room temperature, ethyl acetate was added (20 mL) and the reaction mixture was washed with H_2O (2 × 10 mL) and brine (10 mL). The combined aqueous phases were extracted with ethyl acetate (3 × 10 mL). The combined organic phases were dried over $MgSO_4$, the volatiles removed under reduced pressure, and the crude product was purified by preparative MPLC (SiO_2, 12 g, gradient cyclohexane/ethyl acetate 10:0 to 8:2). **1$_{PD-A/B}$** (80 mg, 127 µmol, 68%) as a mixture of two regioisomers was obtained as a cream solid. Further purification by recrystallization from hot ethyl acetate only achieved partial separation of the two isomers.

1$_{PD-A}$: ***R*$_f$:** 0.40 (SiO_2; ethyl acetate/cyclohexane 1:4); **^{1}H NMR** (500 MHz, $CDCl_3$, 25 °C, sample contains traces of ethyl acetate with q at δ = 4.12, s at δ = 2.04 and t at δ = 1.26): *δ* = 8.49 (dd, 2H, H–C(1,6)), 8.39 (dd, 2H H–C(3,8)), (m, 4H, H_2–C(13,14)), 4.04 (dd, 4H, H_2–C(11,16)), 2.30 (m, 2H, H_{ax}–C(12,15)), 1.53 (m, 2H, H_{eq}–C(12,15)), 1.37 (s, 24H, H_3–C(17,18)) ppm; **^{13}C NMR** (175 MHz, $CDCl_3$, 25 °C), assignments based on ^{1}H,^{13}C-HSQC and HMBC NMR spectra): *δ* = 196.0 (C(9,4)), 138.6 (C(5′,10′)), 137.2 (C(2,7)), 136.2 (C(1,6)), 131.1 (C(3a,5a)), 129.6 (C(3′,8′)), 129.3 (C(3,8)), 92.9 (C(5,10)), 60.5 (C(11,13,14,16)), 25.0 (C(12,15)), 21.1 (C(17,18)), 14.3 (H_3–C(17,18)) ppm; **HRMS** (pos. ESI): *m/z*: $[M + H]^+$ calcd. for

$C_{34}H_{41}B_2O_{10}^+$ 631.2881; found 631.2898. **MP**: 205 °C d. **FT-IR (ATR)**: ṽ = 2877 (C–H), 2928 (C–H), 1718 (C=O) cm^{-1}.

1PD-B: ***R*f**: 0.40 (SiO_2; ethyl acetate/cyclohexane 1:4); **^{1}H NMR** (500 MHz, $CDCl_3$, 25 °C sample contains traces of ethyl acetate with q at δ = 4.12, s at δ = 2.04 and t at δ = 1.26): *δ* = 8.51 (d, 2H, H–C(1,3)), 8.35 (d, 2H H–C(8,6)), 4.52 (m, 4H, H_2–C(13,14)), 4.04 (dd, 4H, H_2–C(11,16)), 2.30 (m, 2H, H_{ax}–C(12,15)), 1.53 (m, 2H, H_{eq}–C(12,15)), 1.37 (s, 24H, H_3–C(17,18)) ppm; **^{13}C NMR** (175 MHz, $CDCl_3$, 25 °C), assignments based on ^{1}H,^{13}C-HSQC and HMBC NMR spectra): *δ* = 195.7 (C(4,10)), 138.7 (C(5′,8′)), 137.2 (C(3a)), 136.5 (C(1,3)), 136.2 (C(2)), 130.5 (C(7)), 129.8 (C(3′,10′)), 129.5 (C(6,8)), 127.8 (C(5a)), 93.0 (C(5,9)), 62.9 (C(11,13,14,16)), 25.0 (C(12,15)), 21.1 (C(17,18,19,20)), 14.3 (H_3–C(17,18,19,20)) ppm; **HRMS** (pos. ESI): *m/z*: [*M* + H]$^+$ calcd. for $C_{34}H_{41}B_2O_{10}^+$ 631.2881; found 631.2898. **MP**: 205 °C d. **FT-IR (ATR)**: ṽ = 2877 (C–H), 2928 (C–H), 1718 (C=O) cm^{-1}.

(±)-Pyrene-bis(propane-1,3-diol)-divinyl-diol (3PD-A)

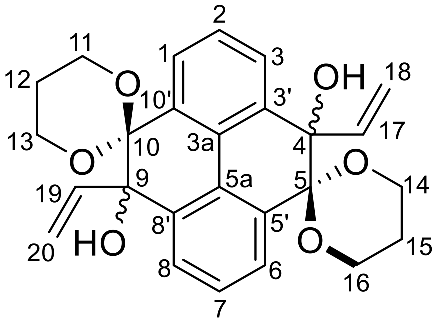

3PD-A

An ice-cooled solution of **PTOPD-A** (30 mg, 0.079 mmol, 1.00 equiv.), in dry tetrahydrofuran (1 mL) was added dropwise to an ice-cooled solution of vinylmagnesium bromide (0.320 mL, 0.320 mmol, 4.00 equiv., 1 M in tetrahydrofuran). After addition, the reaction mixture was stirred at room temperature for 15 h. The mixture was quenched with aqueous ammonium chloride, extracted with ethyl acetate, dried over $MgSO_4$, the volatiles removed under reduced pressure, and the crude product purified by preparative MPLC (SiO_2, 4 g, gradient cyclohexane/ethyl acetate 2:8 to 4:6) to give **3PD-A** (28 mg, 0.064 mmol, 81%) as a cream-colored solid.

***R*f**: 0.21 (SiO_2; ethyl acetate/cyclohexane 1:3); **^{1}H NMR** (500 MHz, $CDCl_3$, 25 °C): *δ* = 7.67 (dd, 4H, H–C(1,3,8,6)), 7.33 (t, 2H, H–C(2,7)), 5.90 (dd, 2H, H–C(17,19)), 4.97 (d, 2H, H_2–C(18,20)), 4.92 (d, 2H, H_2–C(18,20)), 4.51–4.48 (m, 2H, H_2–C(13,14)), 4.40–4.36 (m, 2H, H_2–C(13,14)), 3.76–3.73 (m, 2H, H_2–C(11,16)), 3.67–3.73 (m, 2H, H_2–C(11,16)), 3.11 (s, 2H, H–O), 2.10–2.04 (m, 2H, H_{ax}–C(12,15)), 1.89–1.84 ppm (m, 2H, H_{eq}–C(12,15)); **^{13}C NMR** (175 MHz, $CDCl_3$, 25 °C), assignments based on ^{1}H,^{13}C-HSQC and HMBC NMR spectra): *δ* = 207.1 (C(9,4)), 139.3 (C(5′,10′)), 137.3 (C(2,7)), 133.2 (C(1,6)), 128.8 (C(3a,5a)), 128.0 (C(3,8)), 126.6 (C(18,20)), 126.9 (C(17,19)), 99.6 (C(5,10)), 80.8 (C(3′,8′)), 62.1 (C(11,13,14,16)), 58.8 (C(11,13,14,16)), 25.0 ppm (C(12,15)); **HRMS** (pos. ESI): *m/z*: [*M* + Na]$^+$ calcd. for $C_{26}H_{26}O_6Na^+$ 457.1622; found 457.1619. **FT-IR (ATR)**: ṽ = 3483 (strong broad, O–H), 2923 (C–H), 1084 (C=C) cm^{-1}.

S2.3. Dimethylpropane-1,3-diol (DMPD) Protecting Groups

Pyrene-bis(2,2-dimethylpropane-1,3-diol)ketal (PTO$_{DMPD-A/B}$)

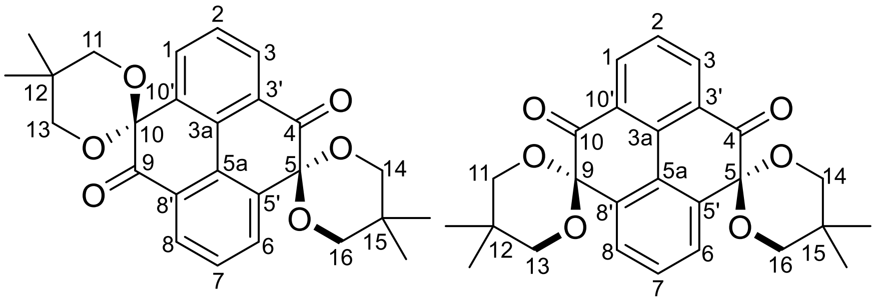

PTO (100 mg, 0.38 mmol, 1.00 equiv.), *p*-toluenesulfonic acid (65.3 mg, 0.34 mmol, 0.9 equiv.) and 2,2-dimethylpropane-1,3-diol (794 mg, 7.63 mmol, 20.0 equiv.) were dissolved in toluene (13 mL, 0.03 M). The mixture was refluxed for 3 h in an oil bath using a DEAN-STARK apparatus. After cooling to room temperature, the reaction mixture was washed with H_2O (2 × 10 mL) and brine (10 mL). The combined aqueous phases were extracted with toluene (3 × 10 mL). The combined organic phases were dried over $MgSO_4$, the volatiles removed under reduced pressure, and the crude product was purified by preparative MPLC (SiO_2, 12 g, gradient cyclohexane/ethyl acetate 10:0 to 8:2). **PTO$_{DMPD-A/B}$** (150 mg, 0.35 mmol, 90%) as a mixture of two regioisomers was obtained as a yellow solid.

PTO$_{DMPD-A}$: *R*$_f$: 0.47 (SiO_2; ethyl acetate/cyclohexane 1:4); **^{1}H NMR** (700 MHz, $CDCl_3$, 25 °C, sample contains traces of H_2O with s at δ = 1.53): *δ* = 8.15 (dd, 2H, H–C(1,6)), 7.94 (dd, 2H H–C(3,8)), 7.48 (t, 2H, H–C(2,7)), 4.22 (t, 4H, H_2–C(13,14)), 3.58 (t, 4H, C(11,16)), 1.31 (s, 6H, H_3–C(12,15)), 0.82 ppm (s, 6H, H_3–C(12,15)); **^{13}C NMR** (175 MHz, $CDCl_3$, 25 °C), assignments based on ^{1}H,^{13}C-HSQC and HMBC NMR spectra): *δ* = 195.5 (C(9,4)), 136.7 (C(5′,10′)), 132.67 (C(1,6)), 131.3 (C(3a,5a)), 129.8 (C(3′,8′)), 128.7 (C(3,8)), 92.5 (C(5,10)), 30.0 (C(11,13,14,16)), 23.3 (C(12,15)), 22.1 ppm (H_3–C (12,15)); **HRMS** (pos. ESI): *m/z*: $[M + H]^+$ calcd. for $C_{26}H_{27}O_6^+$ 435.1803; found 435.1802. **MP**: 195.8 – 196.1 °C. **FT-IR (ATR)**: ṽ = 2912 (C–H), 1686 (strong, C=O) cm^{-1}.

PTO$_{DMPD-B}$: ***R*$_f$**: 0.47 (SiO_2; ethyl acetate/cyclohexane 1:4); **^{1}H NMR** (700 MHz, $CDCl_3$, 25 °C, sample contains traces of H_2O with s at δ = 1.53): *δ* = 8.07 (d, 2H, H–C(1,3)), 8.01 (d, 2H H–C(8,6)), 7.52 (t, 1H, H–C(2)), 7.47 (t, 1H, H–C(7)), 4.22 (t, 4H, H_2–C(13,14)), 3.58 (t, 4H, C(11,16)), 1.31 (s, 6H, H_3–C(12,15)), 0.82 (s, 6H, H_3–C(12,15)) ppm; **^{13}C NMR** (175 MHz, $CDCl_3$, 25 °C), assignments based on ^{1}H,^{13}C-HSQC and HMBC NMR spectra): *δ* = 195.0 (C(4,10)), 137.0 (C(5′,8′)), 135.4 (C(3a)), 132.6 (C(1,3)), 130.7 (C(7)), 129.9 (C(3′,10′)), 129.0 (C(2)), 128.7 (C(6,8)), 126.0 (C(5a)), 92.6 (5,9), 30.0 (C(11,13,14,16)), 23.3 (C(12,15)), 22.2 (H_3–C (12,15)) ppm; **HRMS** (pos. ESI): *m/z*: [*M* + H]$^+$ calcd. for $C_{26}H_{27}O_6^+$ 435.1803; found 435.1802. **MP**: 195.8 – 196.1 °C. **FT-IR (ATR)**: ṽ = 2965 (C–H), 1697 (strong, C=O) cm^{-1}.

S2.4. General Procedure for the Deprotection of Protected PTOs

A mixture of protected-**PTO** (11 µmol) in a solvent mixture of $CHCl_3$/TFA/H_2O (0.01 M **PTO**; ratio given below) was stirred until full conversion to **PTO** was observed, monitored via TLC (1:1 ethyl acetate/cyclohexane). The mixture was quenched with 2 M aqueous K_2CO_3, then washed with H_2O and brine. The organic layer was dried over $MgSO_4$, filtered, and concentrated under reduced pressure to give **PTO** as an orange solid.

Deprotection of propane-1,3-diol-protected PTO (PTO$_{PD-A/B}$)

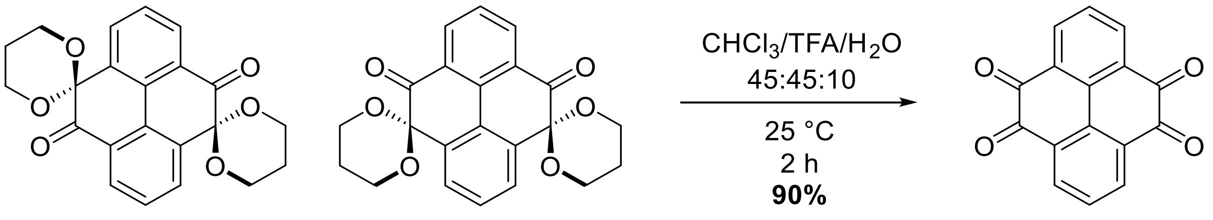

PTO$_{PD-A}$ **PTO$_{PD-B}$** **PTO**

A mixture of **PTO$_{PD-A/B}$** (64 mg, 0.17 mmol) in $CHCl_3$/TFA/H_2O 45:45:10 (17.1 mL) was stirred at 25 °C for 2 h. Full conversion to **PTO** was observed via TLC (1:1 ethyl acetate/cyclohexane). The mixture was quenched with 2 M aqueous K_2CO_3, then washed with H_2O (10 mL) and brine (10 mL). The organic layer was dried over $MgSO_4$, filtered, and concentrated under reduced pressure to give **PTO** as an orange solid (40 mg, 0.15 mmol, 90%). The characterization agrees with the literature.[5]

S2.5. Decomposition Experiments

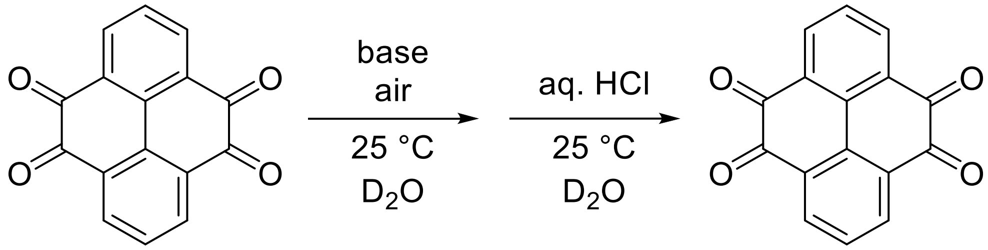

PTO **PTO**

An NMR tube with **PTO** (5 mg, 0.020 mmol), under air atmosphere and at 25 °C, was treated with a 1 M solution of KOH or NaOH in D_2O. The solution became dark immediately, and an insoluble black precipitate was formed. The reaction mixture was then reacidified with a few drops of aq. HCl. The resulting precipitate was extracted with chloroform, dried over $MgSO_4$, and the solvents were removed under reduced pressure to recover **PTO** as a yellow solid (KOH: 1.9 mg, 40% recovered; NaOH: 3.3 mg, 70% recovered).

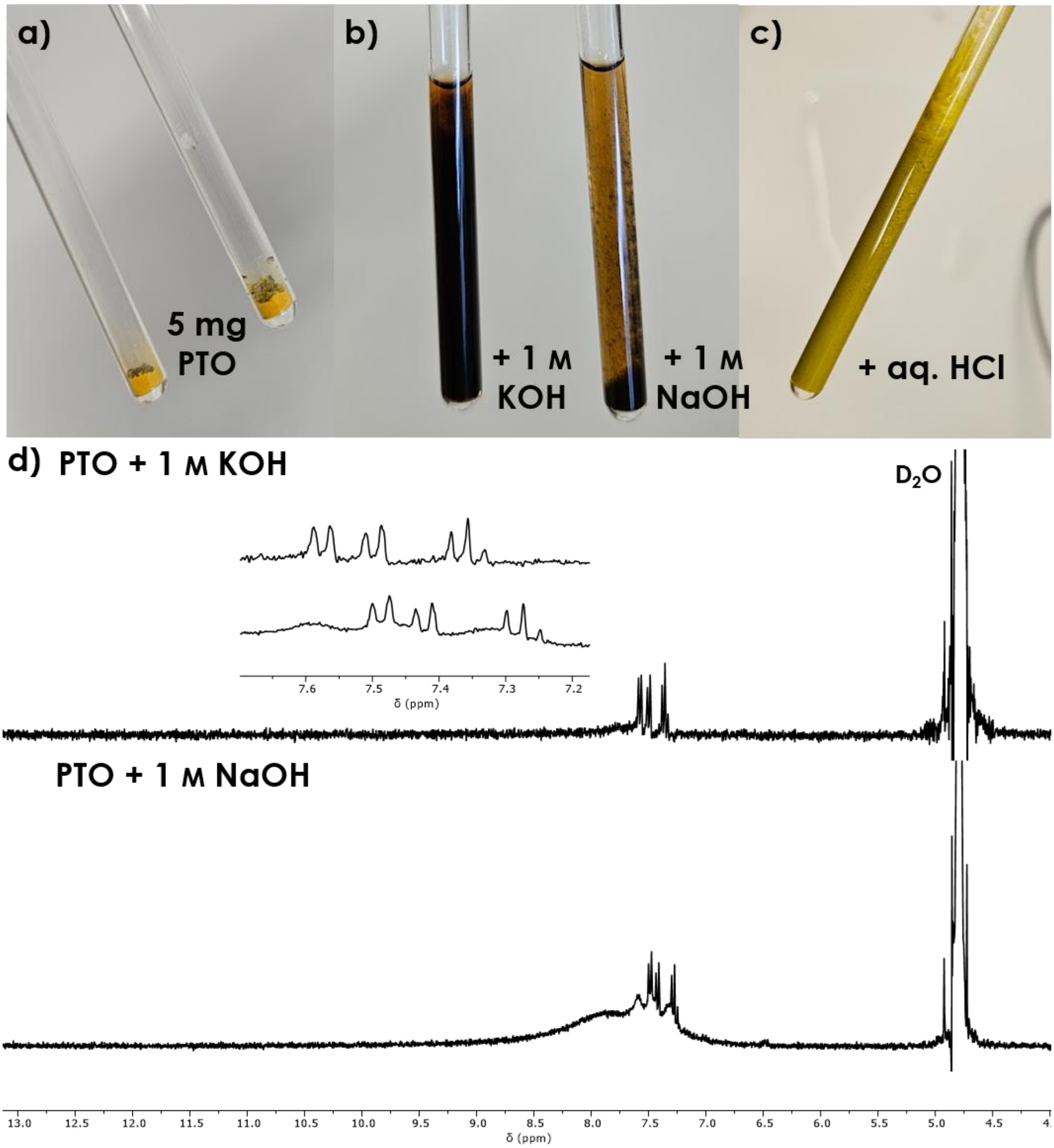

Figure S2. a) Initial **PTO** solid in NMR tube; b) upon addition of 1 M KOH or 1 M NaOH, immediate darkening of solution and black insoluble precipitate formation which is completely insoluble in common solvents ($CHCl_3$, CH_2Cl_2, H_2O, DMSO); c) reacidification of reaction mixture with aq. HCl allowed recovery of PTO but with reduced yields (KOH: 40%, NaOH: 70%); d) ^1H NMR (300 MHz, D_2O, 25 °C) comparison between KOH and NaOH basified solutions of PTO. As the main product of **PTO** decomposition is an insoluble black precipitate, this could not be identified via NMR.

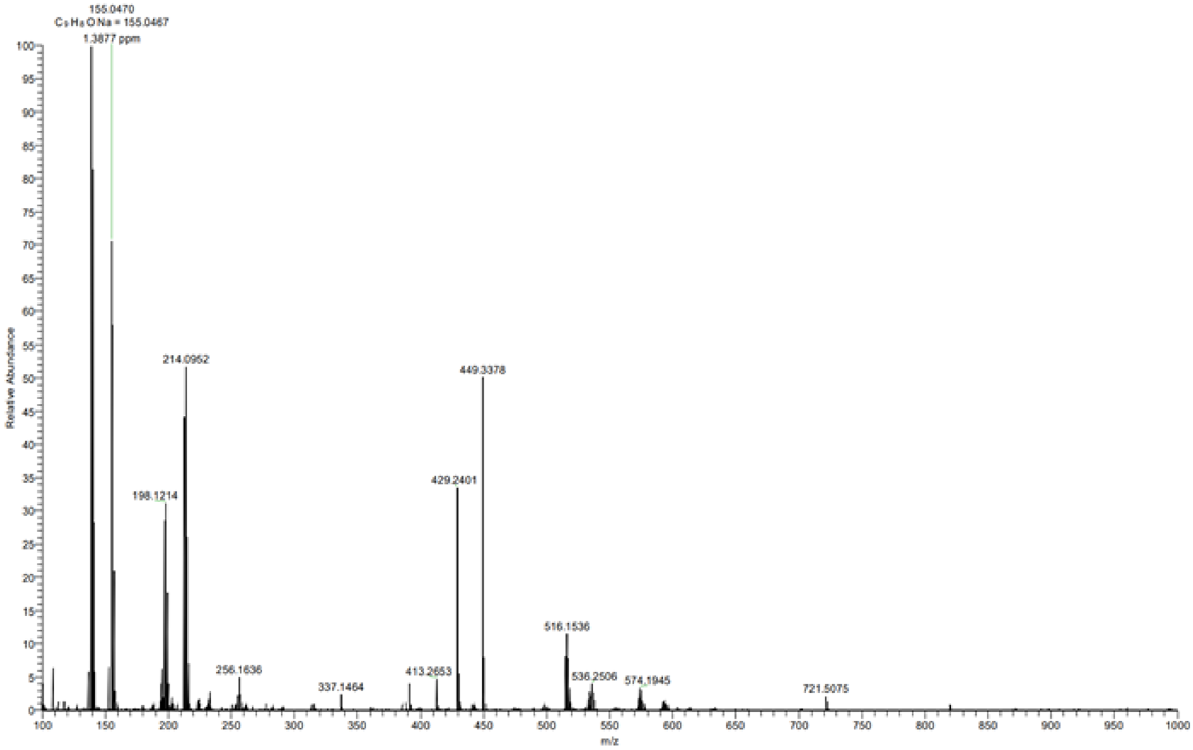

Figure S3. a) HRMS (ESI pos.) of decomposition experiment of **PTO** treated with 1 M KOH.

S3. Structural Analysis of PTO$_{PrG}$

In contrast to **PTO$_{EtG}$** (**Figure S4a**, left), in **PTO$_{PrG}$**, the symmetry of the 1,4-dioxane rings is broken by the additional methyl group (**Figure S4**a, right). This results in three inequivalent protons (1 × methine, 1 × methylene *anti*-methyl and 1 × methylene *syn*-methyl), as shown in **Figure S4**a. Using racemic propylene glycol, the methyl group of each protective group can adopt two different regio-orientations, each of which can then have two different stereo-orientations. Within NMR spectroscopy, each of the two sides of the pyrene bearing two 1,4-dioxane rings can be considered independent. Thus, two different "regioisomeric semi-structures" can be observed, as the methyl groups on each side can be oriented either in the same (**I**, **Figure S4**b) or in opposite directions (**II**, **Figure S4**b). Consequently, three signals are observed for the three protons of I and another three for the three protons of **II**. The additional stereoirregularity of the methyl groups results in four stereoisomers for **I** and **II**, which only slightly differ in their chemical shifts, i.e. they overlap and produce the chaotic form of all the signals visible in **Figure S4**c.

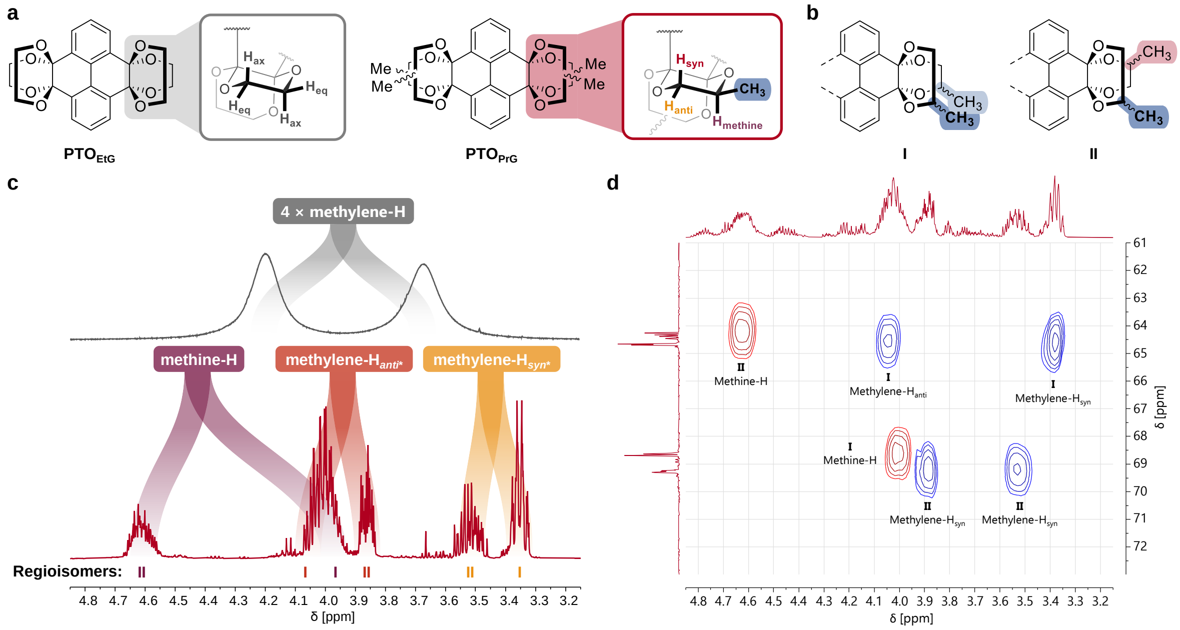

Figure S4. a) Idealized representation of the 1,4-dioxane rings on **PTO$_{EtG}$** and **PTO$_{PrG}$** as chair conformations with proton assignment; b) Possible orientations for the methyl groups on each side of the protected pyrene **PTO$_{PrG}$**. Every side of the pyrene can form one of two "regioisomeric semi-structures" **I** or **II**; c) Sections of the ^{1}H-NMR spectra ($CDCl_3$, 500 MHz) of **PTO$_{EtG}$** (top) and **PTO$_{PrG}$** (bottom) showing the methylene and methine resonance. For **PTO$_{PrG}$,** the double signals for each proton are assigned by line connections in the respective colors. Assignment is based on 2D spectra. For full spectrum see section S4. The assignment

of the *syn*- and *anti*-proton is interchangeable (*) d) Section of the edited HSQC spectrum ($CDCl_3$, 600/151 MHz; blue = CH_2, red = CH & CH_3) of **PTO_{PrG},** showing that the five signals visible in the ^{1}H-NMR spectrum can be attributed to the two "regioisomers" **I** and **II**. Assignment is interchangeable. For full spectrum and measurement parameters, see section S4.

The presence of both **I** and **II** is evident from an edited ^{1}H,^{13}C-hetero single quantum coherence (HSQC) NMR experiment (**Figure S4**d). The assignments of which cross peaks represent **I** and which represent **II** are interchangeable, as are the assignments of the respective *syn*- and *anti*-oriented methylene protons. The determination of which of the three cross peaks belong to the same "regioisomeric semi-structure" was based on a ^{1}H,^{1}H-correlation spectroscopy (COSY) experiment, which can be found in **Figure S9** and **Figure S10**.

S4. Selected NMR Spectra

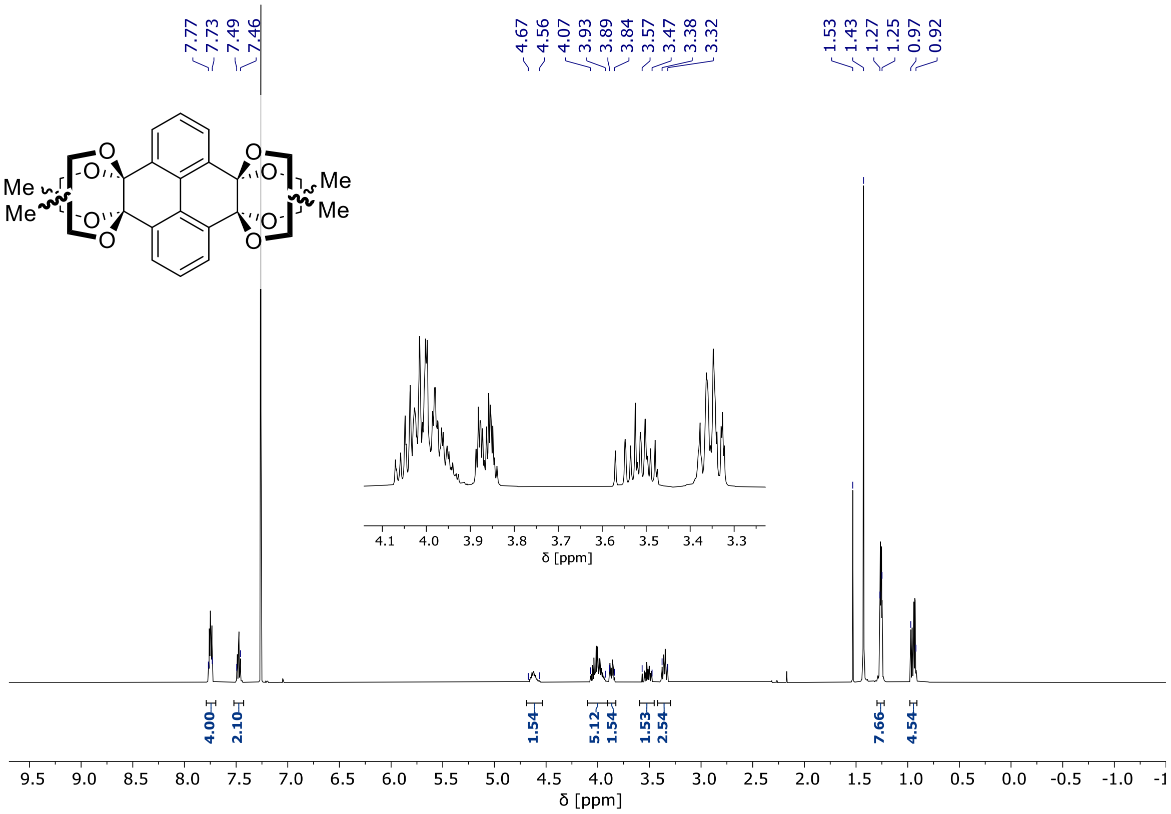

Figure S5. ^{1}H NMR spectrum (500 MHz, $CDCl_3$, 25 °C) of $\mathbf{PTO_{PrG}}$.

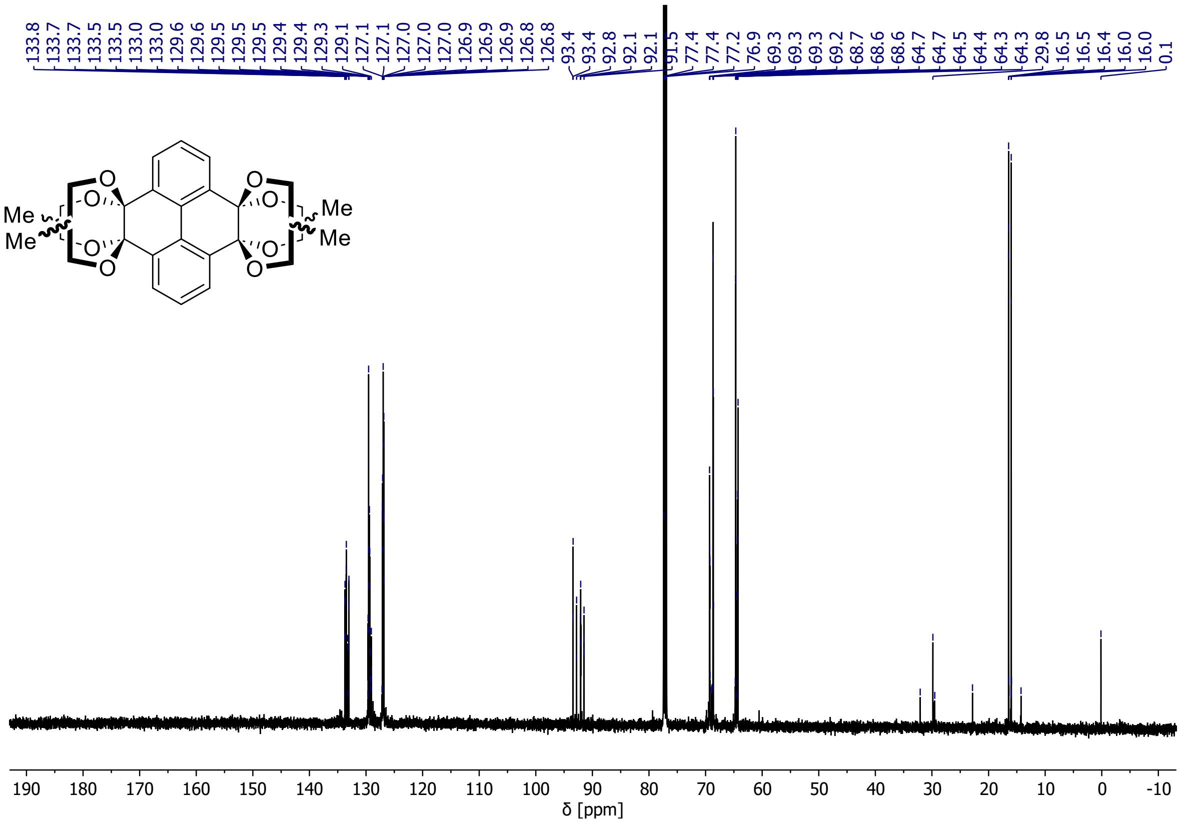

Figure S6. ^{13}C NMR spectrum (125 MHz, $CDCl_3$, 25 °C) of $\mathbf{PTO_{PrG}}$.

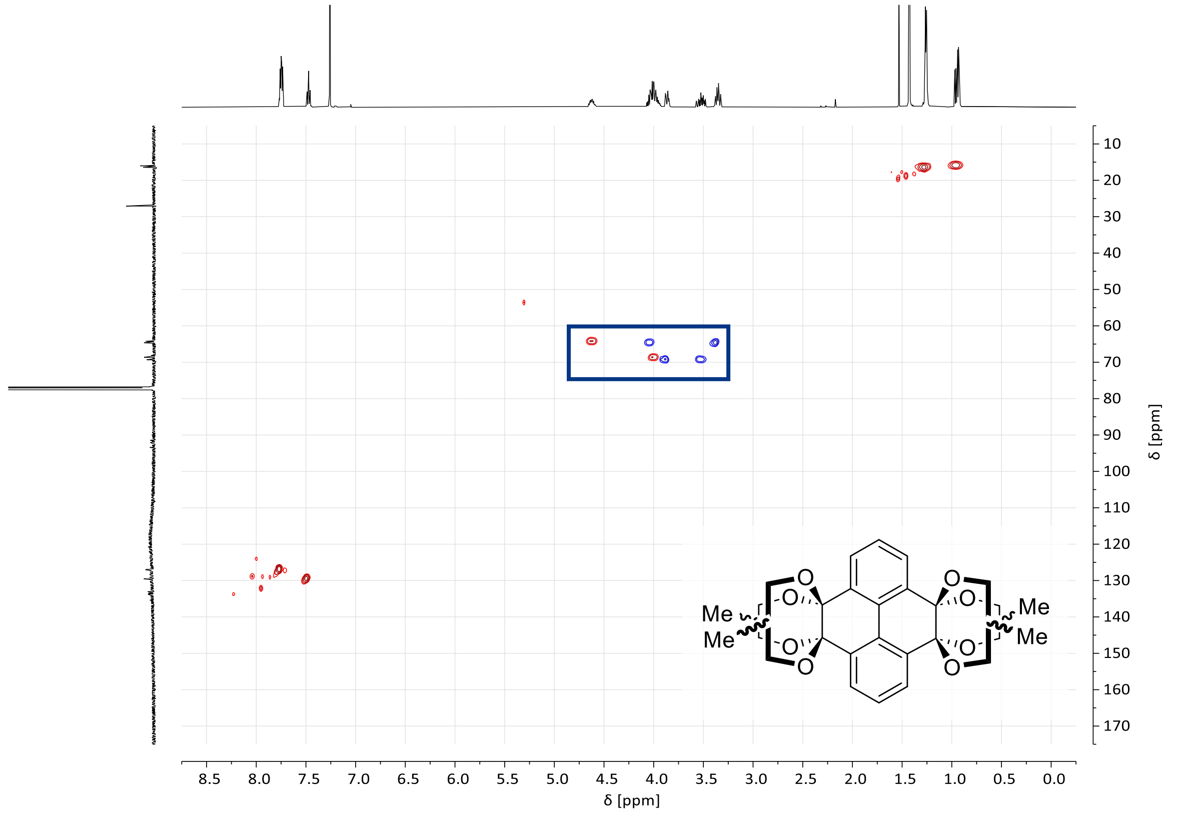

Figure S7. Edited ^{1}H-, ^{13}C-HSQC spectrum (600 MHz; 150 MHz, $CDCl_3$, 25 °C) (Blue = CH_2, Red = CH & CH_3) of **PTO$_{PrG}$**. Blue box indicates the magnified view shown in **Figure S8**.

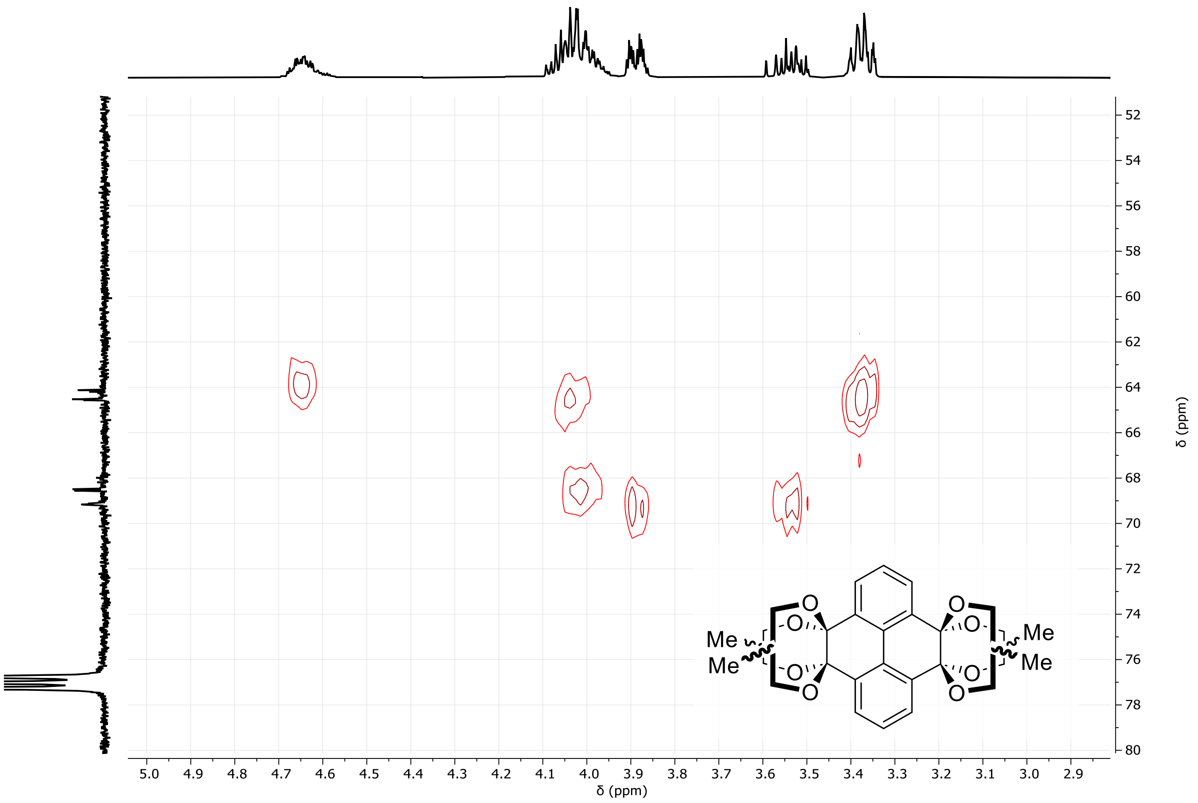

Figure S8. Magnified section of HSQC spectrum (600 MHz; 150 MHz, $CDCl_3$, 25 °C) of **PTO$_{PrG}$**.

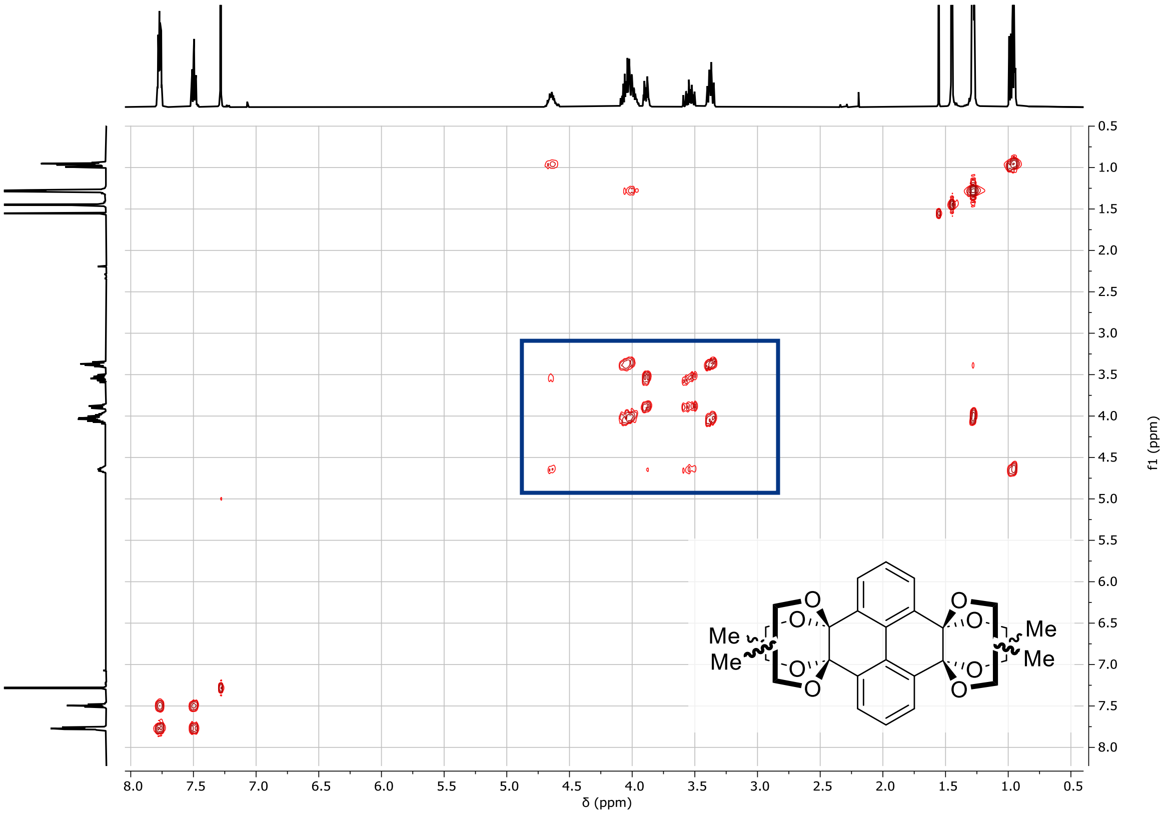

Figure S9. Full COSY spectrum (600 MHz; 600 MHz, $CDCl_3$, 25 °C) of **PTO$_{PrG}$**. Blue box indicates the magnified view shown in **Figure S10**.

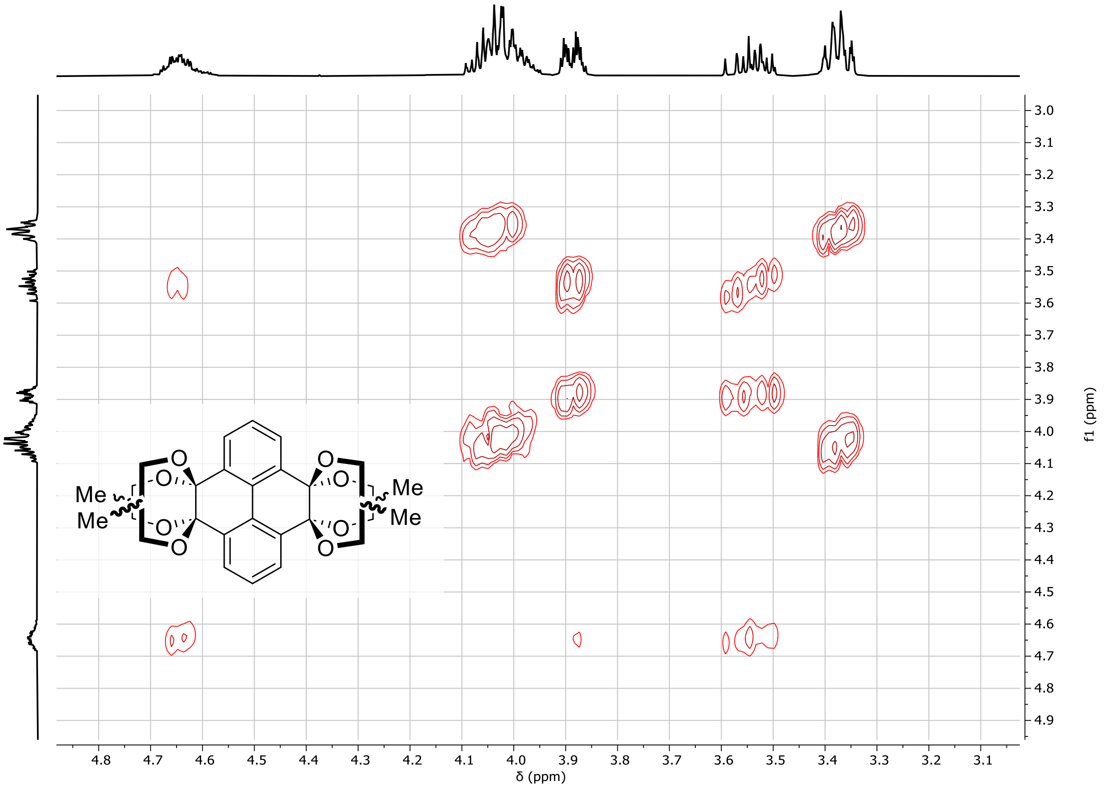

Figure S10. Section of COSY spectrum (600 MHz; 600 MHz, $CDCl_3$, 25 °C) of **PTO$_{PrG}$**.

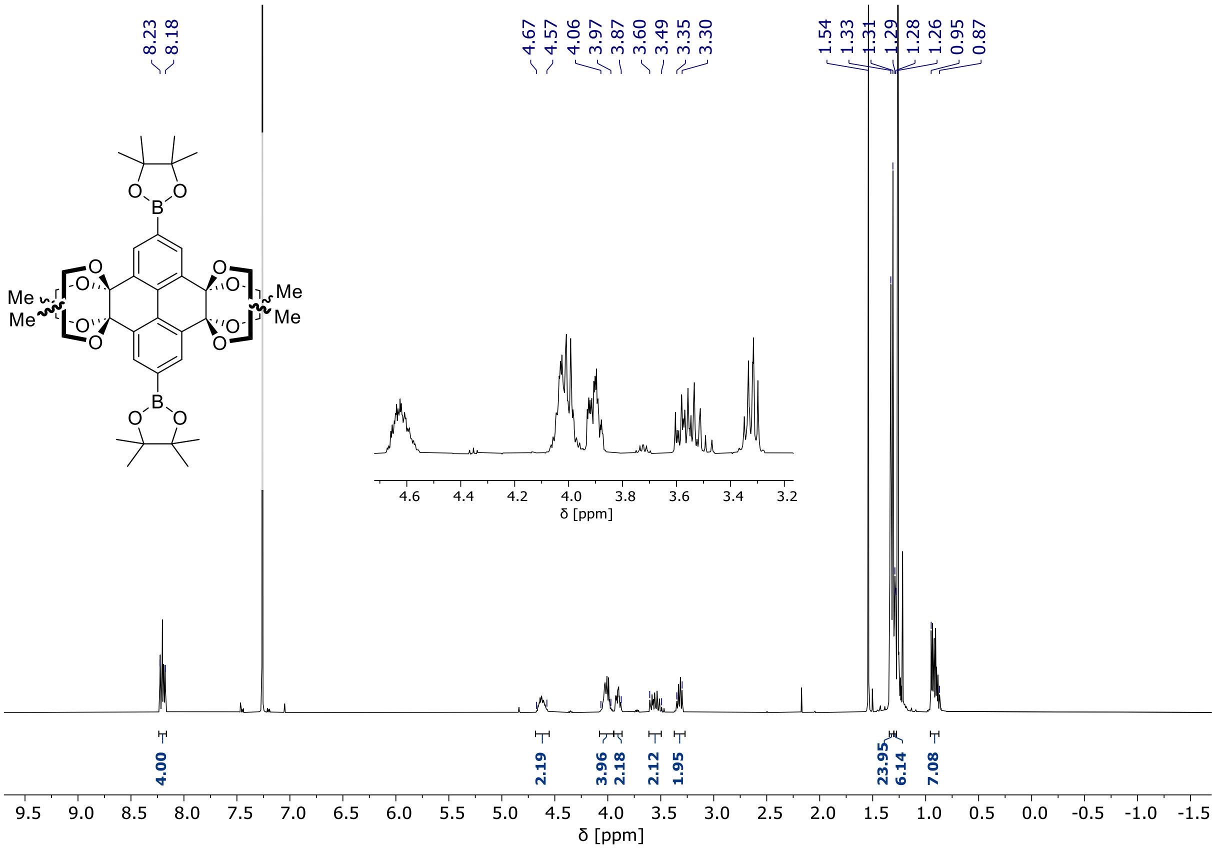

Figure S11. ^{1}H NMR spectrum (500 MHz, $CDCl_3$, 25 °C) of $\mathbf{1}_{PrG}$.

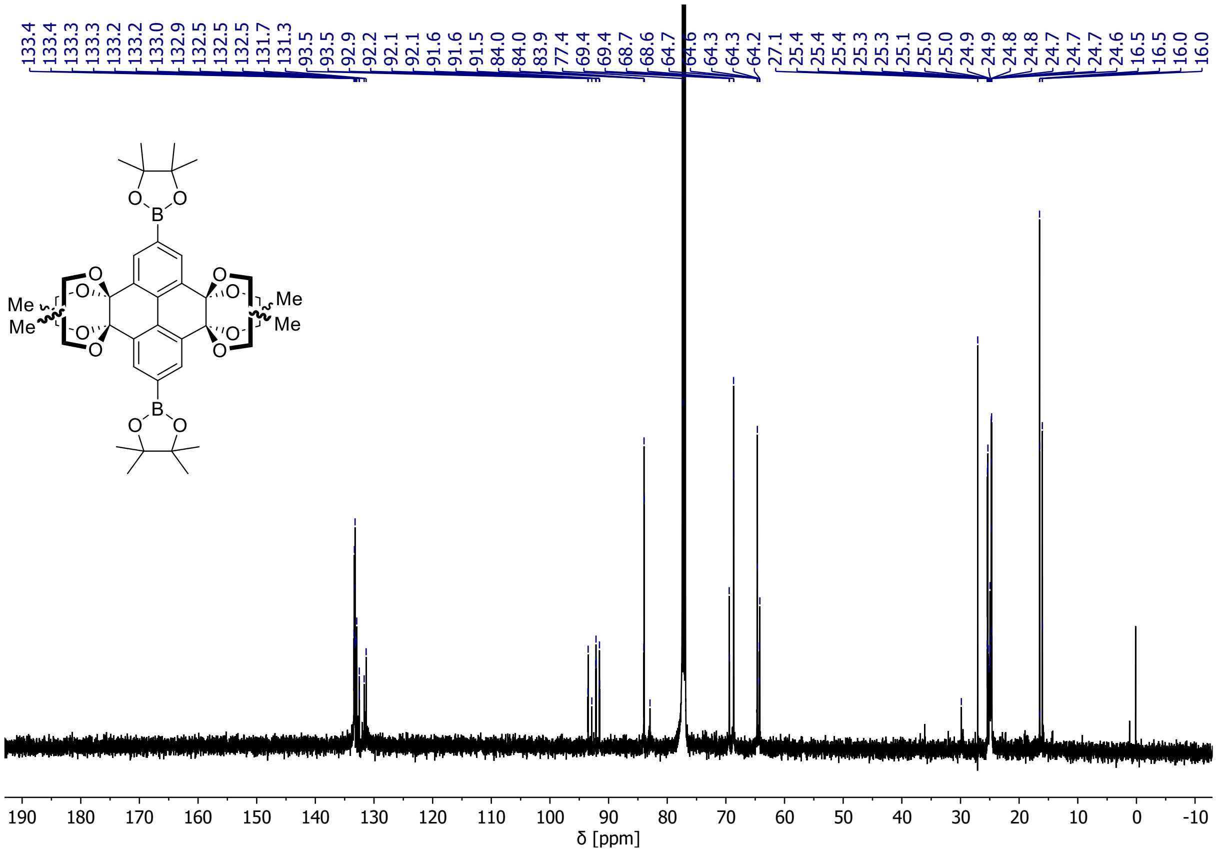

Figure S12. ^{13}C NMR spectrum (125 MHz, $CDCl_3$, 25 °C) of $\mathbf{1}_{PrG}$.

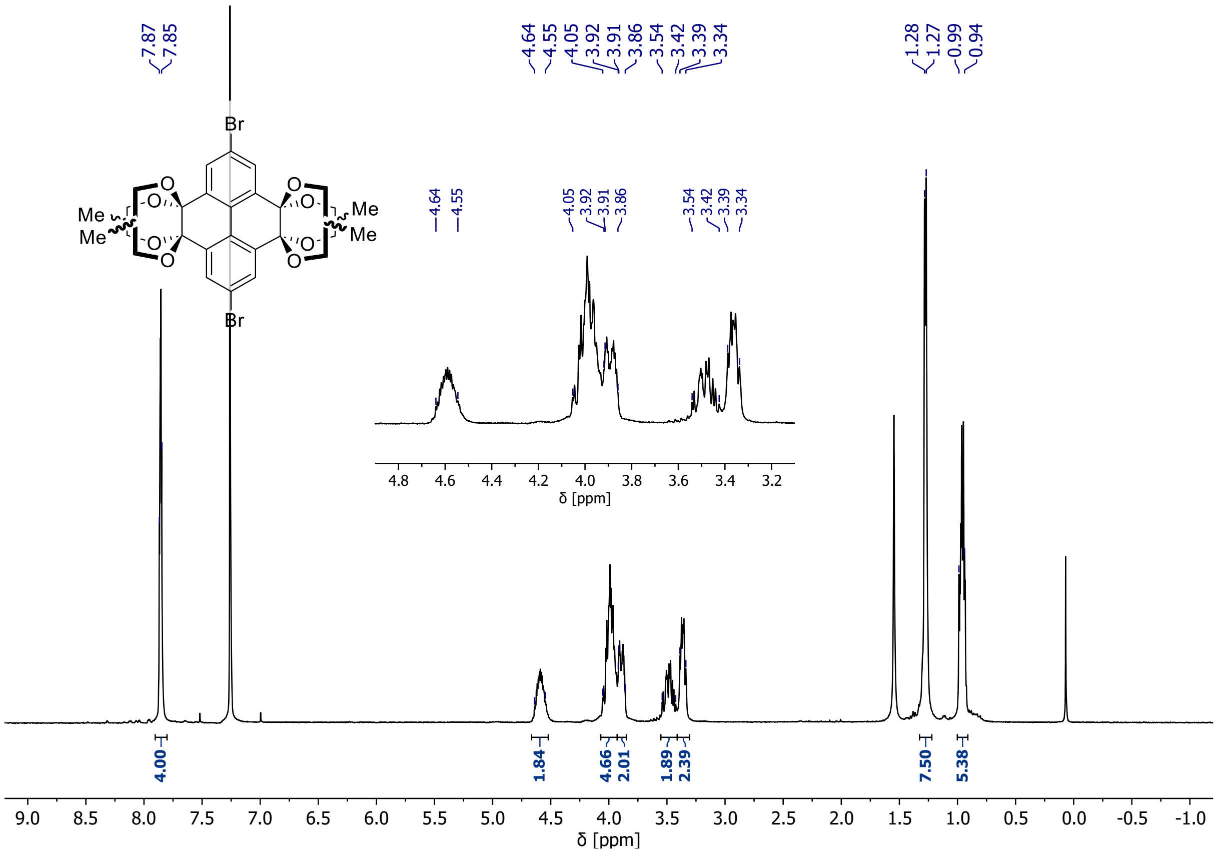

Figure S13. ^{1}H NMR spectrum (400 MHz, $CDCl_3$, 25 °C) of $\mathbf{2}_{\mathbf{PrG}}$.

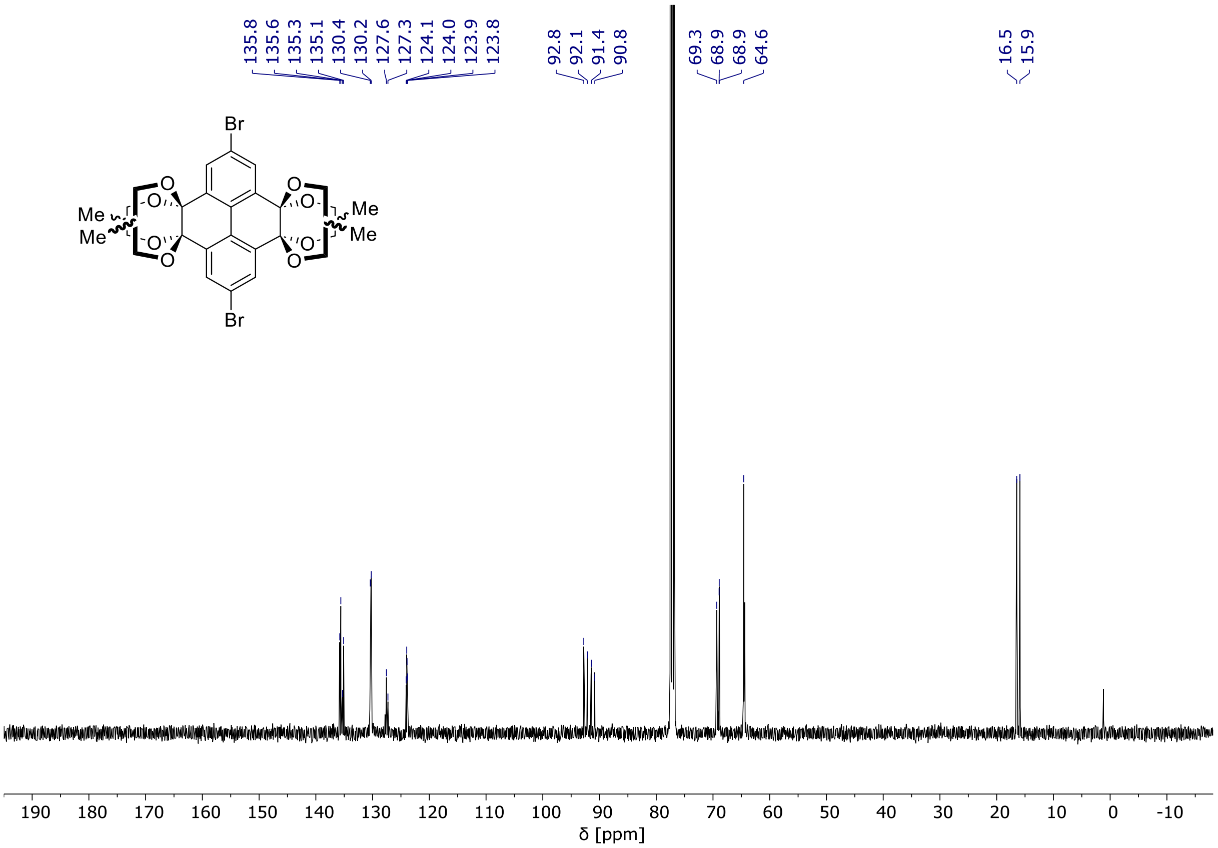

Figure S14. ^{13}C NMR spectrum (100 MHz, $CDCl_3$, 25 °C) of $\mathbf{2}_{\mathbf{PrG}}$.

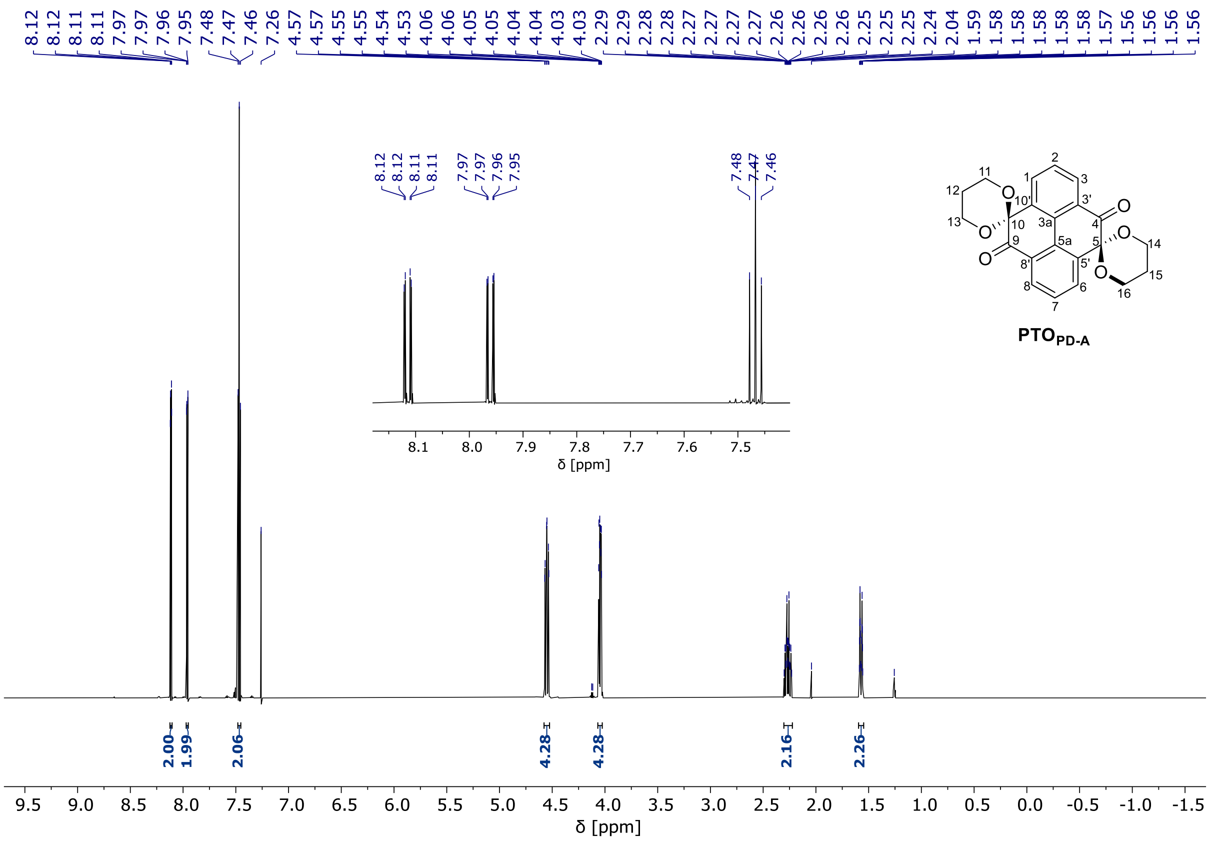

Figure S15. ^{1}H NMR spectrum (700 MHz, $CDCl_3$, 25 °C) of **PTO_{PD-A}**.

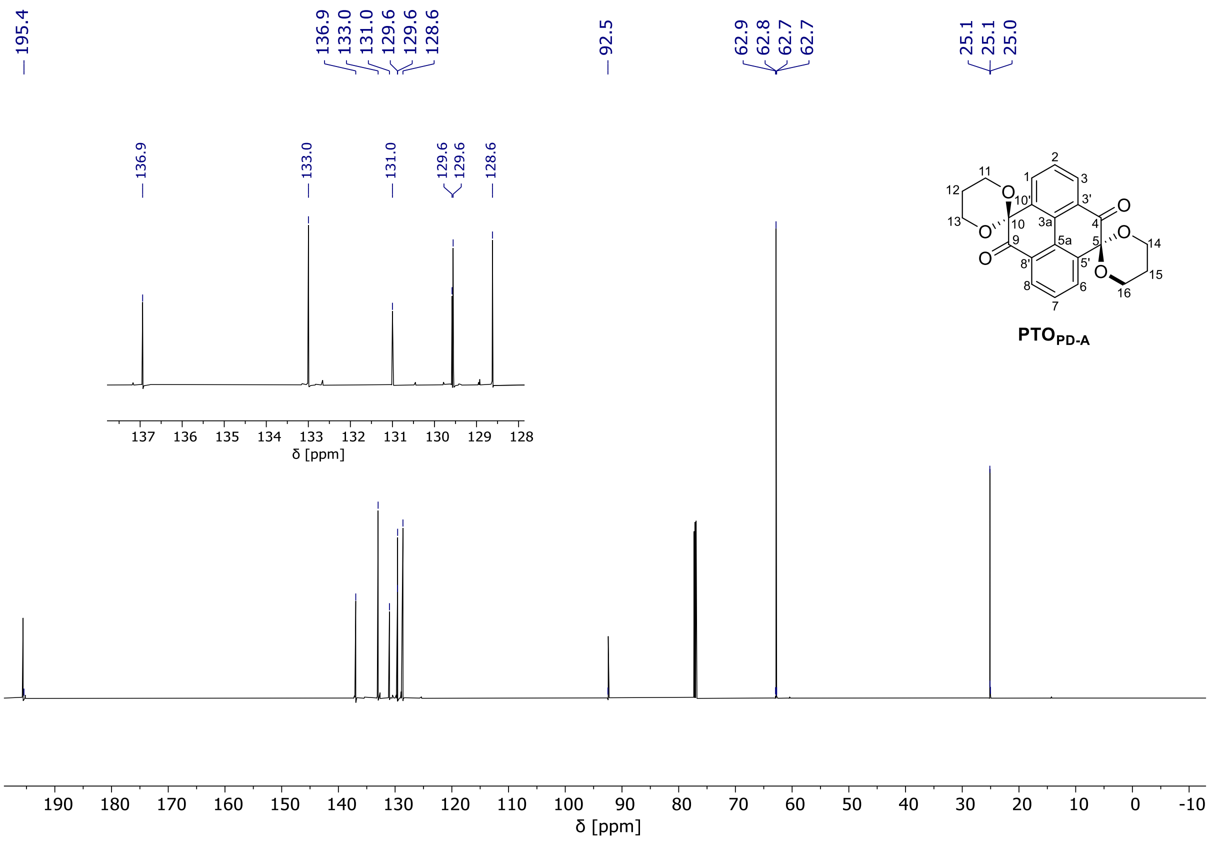

Figure S16. ^{13}C NMR spectrum (175 MHz, $CDCl_3$, 25 °C) of **PTO_{PD-A}**.

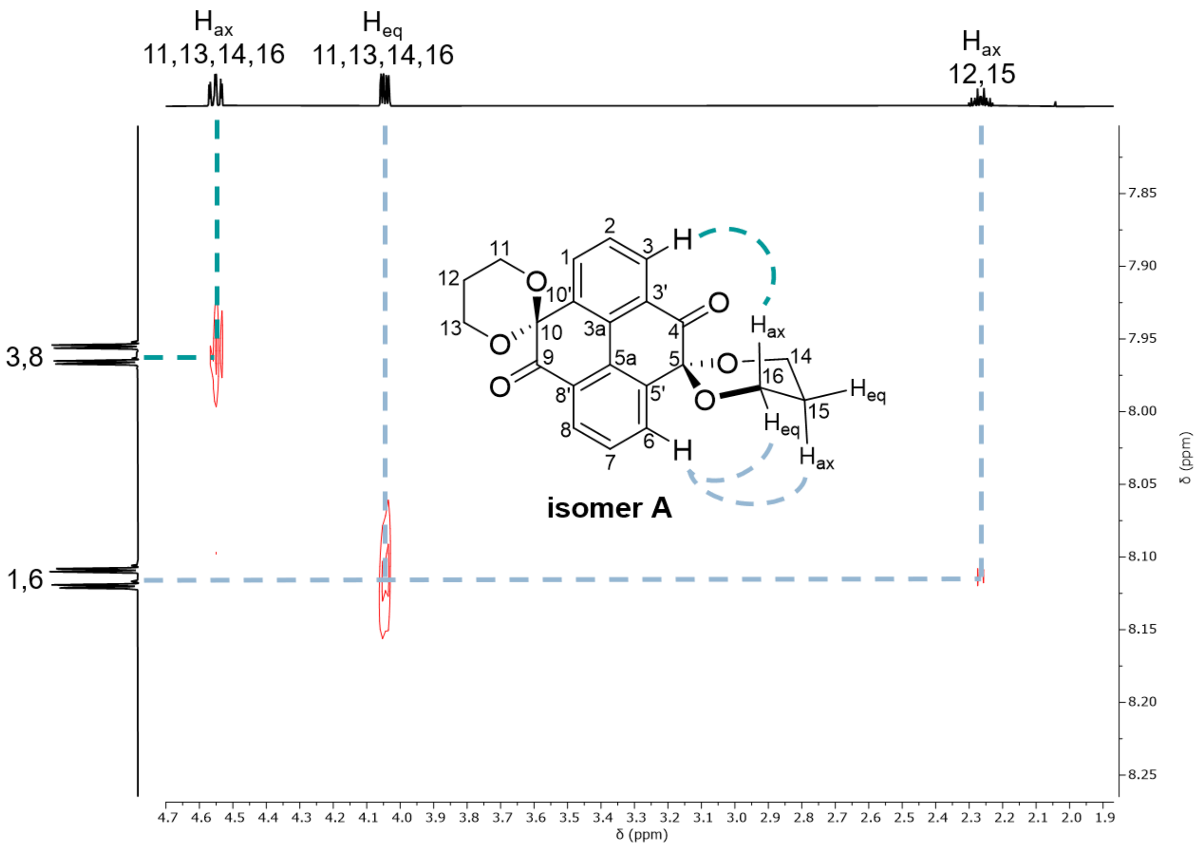

Figure S17. ^{1}H,^{1}H-NOSEY NMR spectrum (700 MHz, $CDCl_3$, 25 °C) of **PTO$_{PD-A}$**.

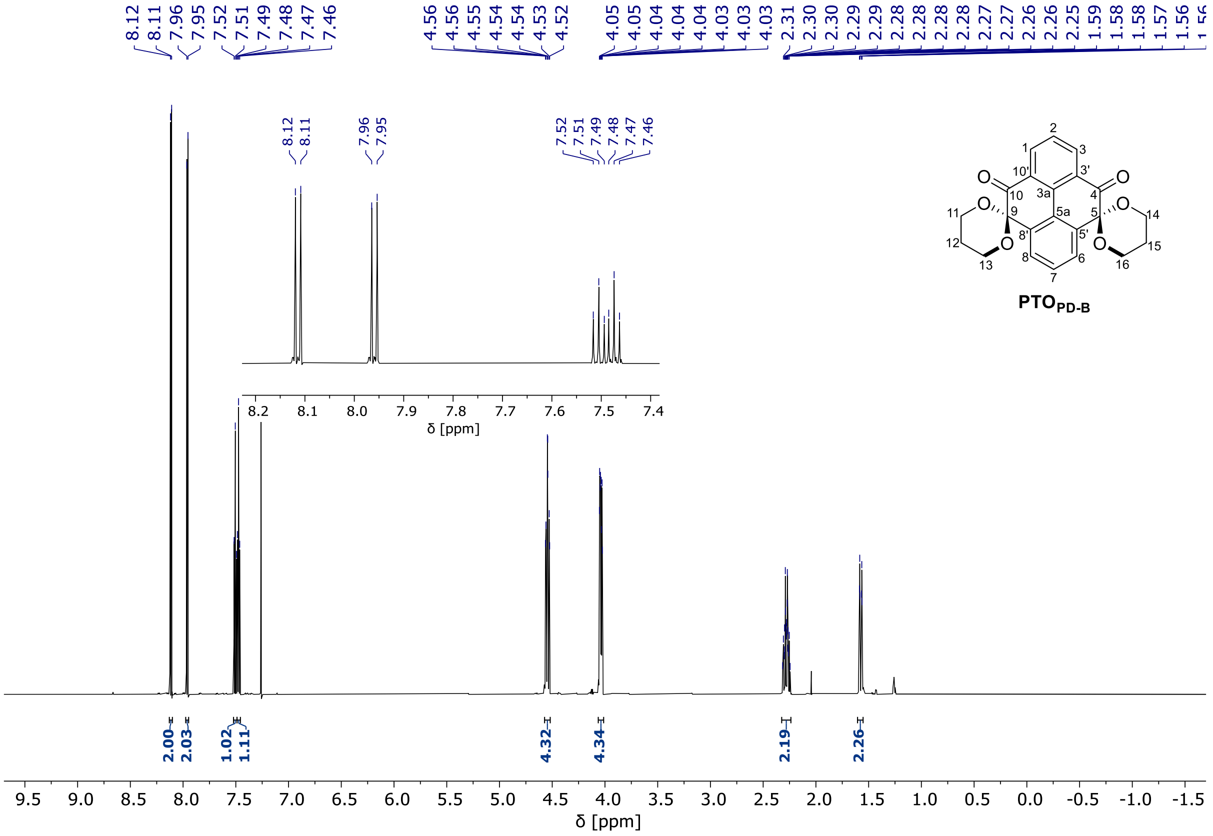

Figure S18. ^{1}H NMR spectrum (700 MHz, $CDCl_3$, 25 °C) of **PTO$_{PD-B}$**.

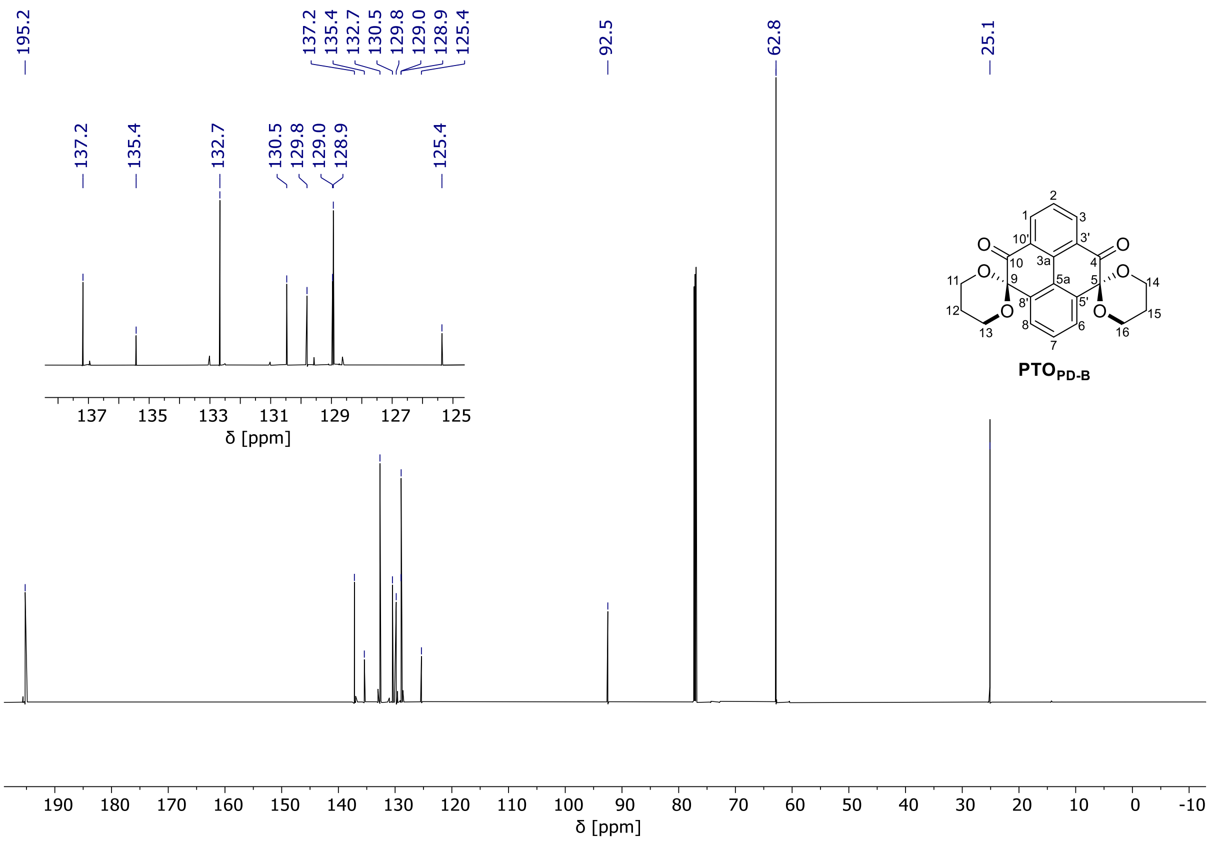

Figure S19. ^{13}C NMR spectrum (175 MHz, $CDCl_3$, 25 °C) of **PTO$_{PD-B}$**.

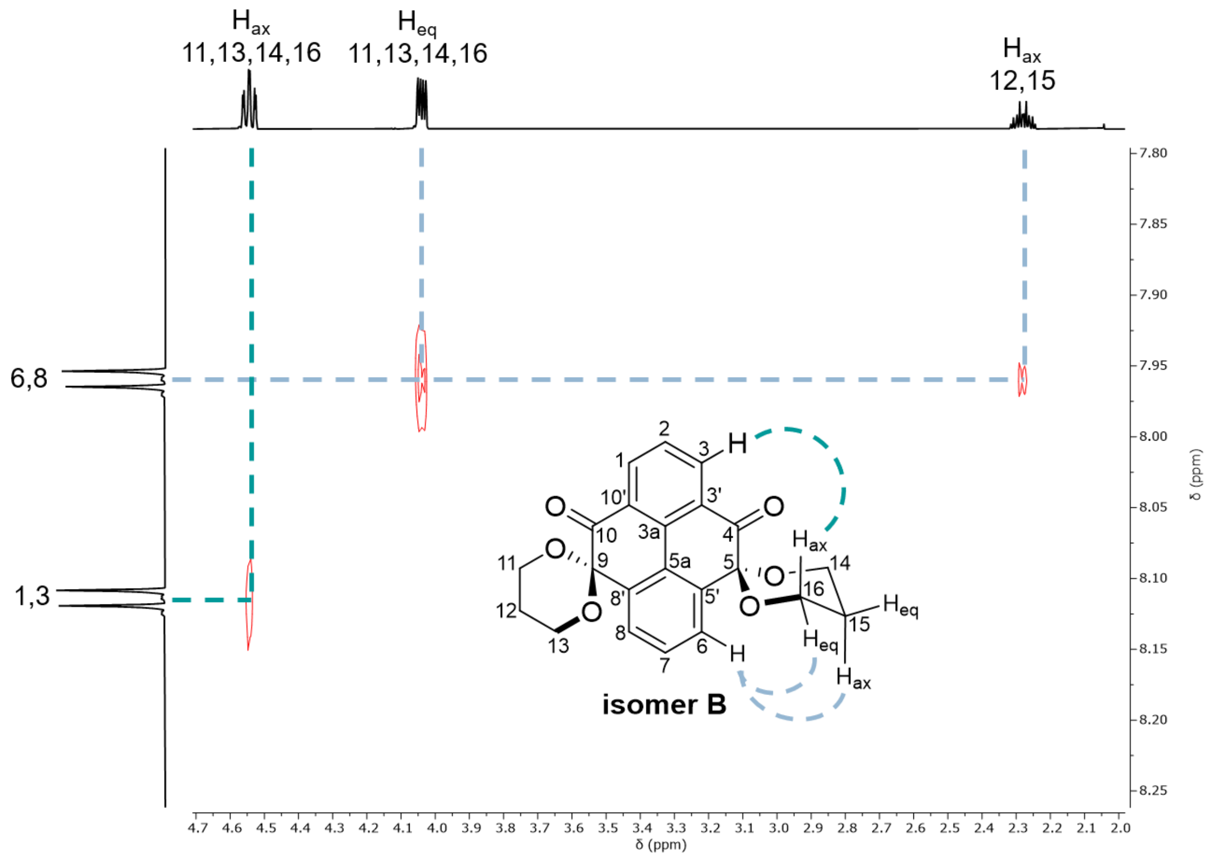

Figure S20. $^{1}H,^{1}H$-NOSEY NMR spectrum (700 MHz, $CDCl_3$, 25 °C) of **PTO$_{PD-B}$**.

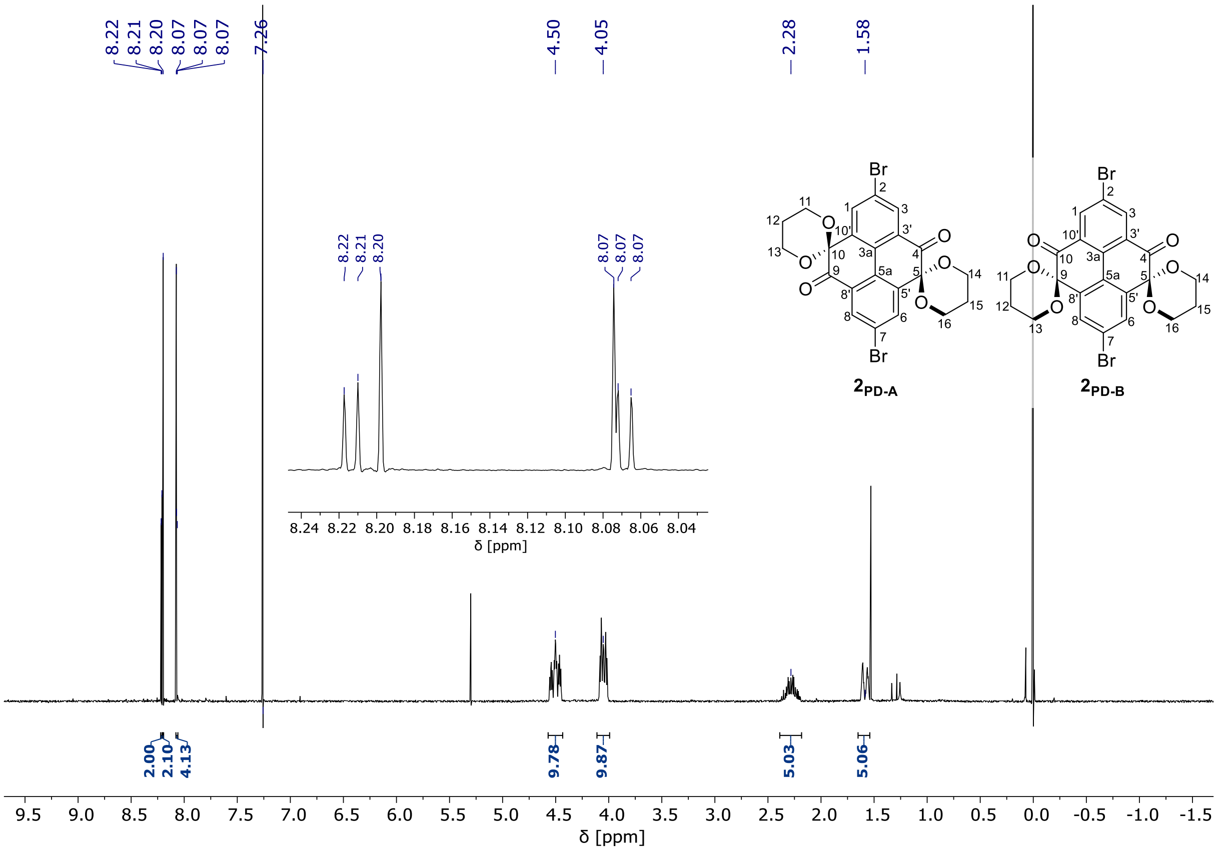

Figure S21. ^{1}H NMR spectrum (500 MHz, $CDCl_3$, 25 °C) of isomeric mixture **$2_{PD-A/B}$**.

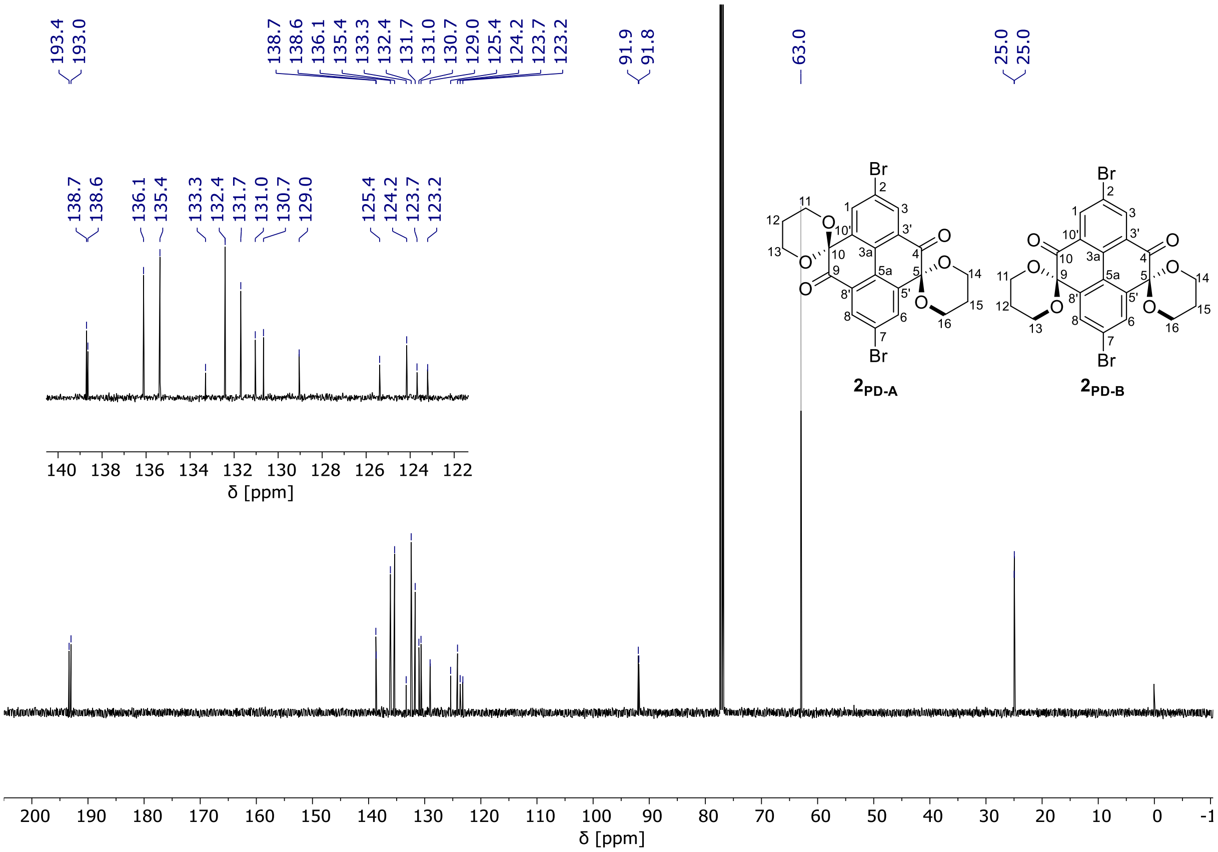

Figure S22. ^{13}C NMR spectrum (125 MHz, $CDCl_3$, 25 °C) of isomeric mixture **$2_{PD-A/B}$**.

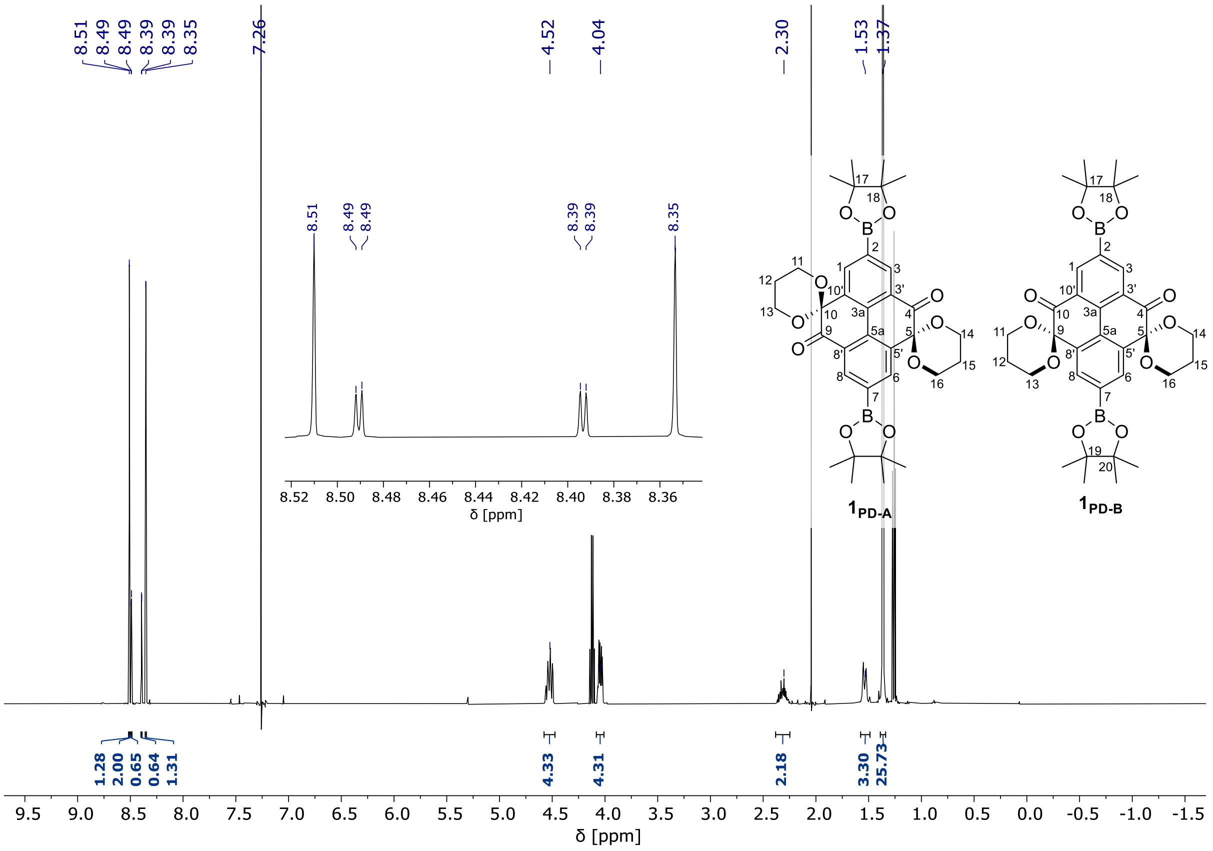

Figure S23. ^{1}H NMR spectrum (700 MHz, $CDCl_3$, 25 °C) of isomeric mixture $\mathbf{1_{PD\text{-}A/B}}$.

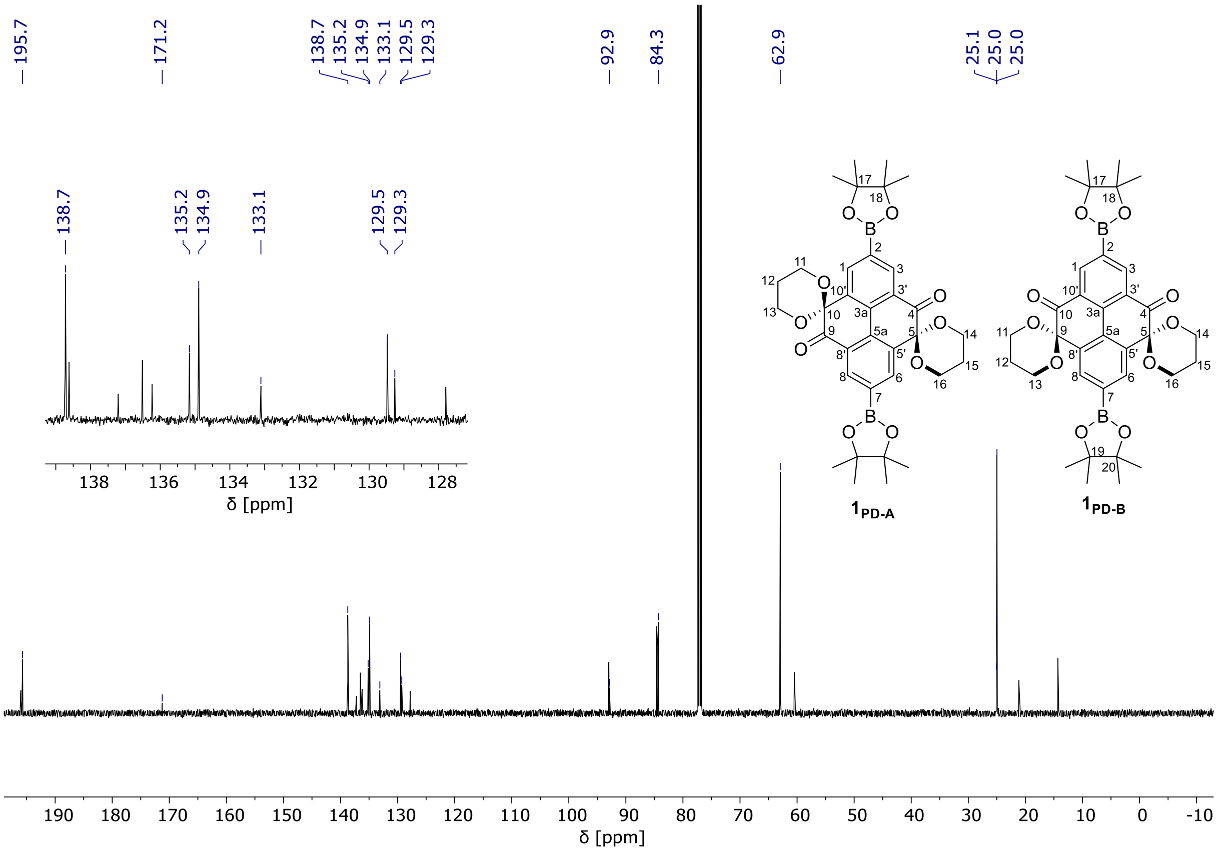

Figure S24. ^{13}C NMR spectrum (175 MHz, $CDCl_3$, 25 °C) of isomeric mixture $\mathbf{1_{PD\text{-}A/B}}$.

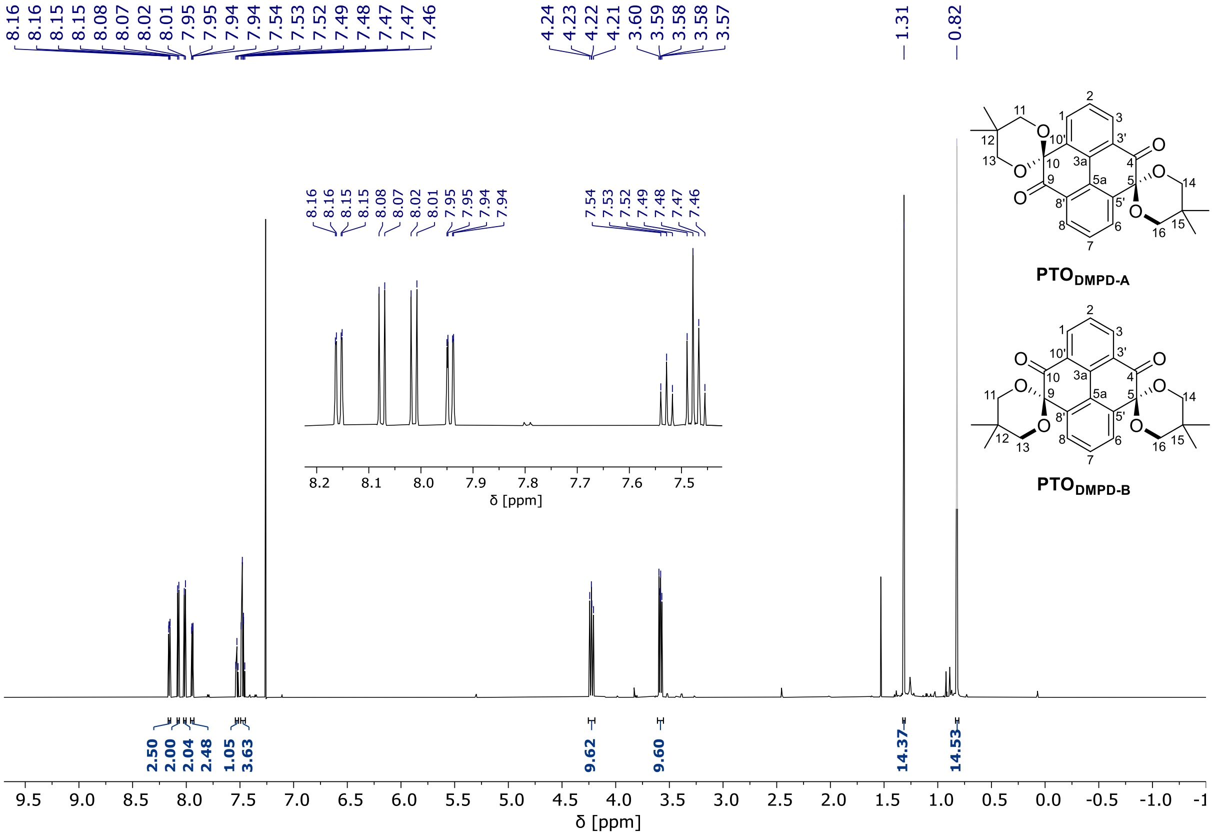

Figure S25. ^{1}H NMR spectrum (700 MHz, $CDCl_3$, 25 °C) of isomeric mixture **PTO$_{DMPD-A/B}$**.

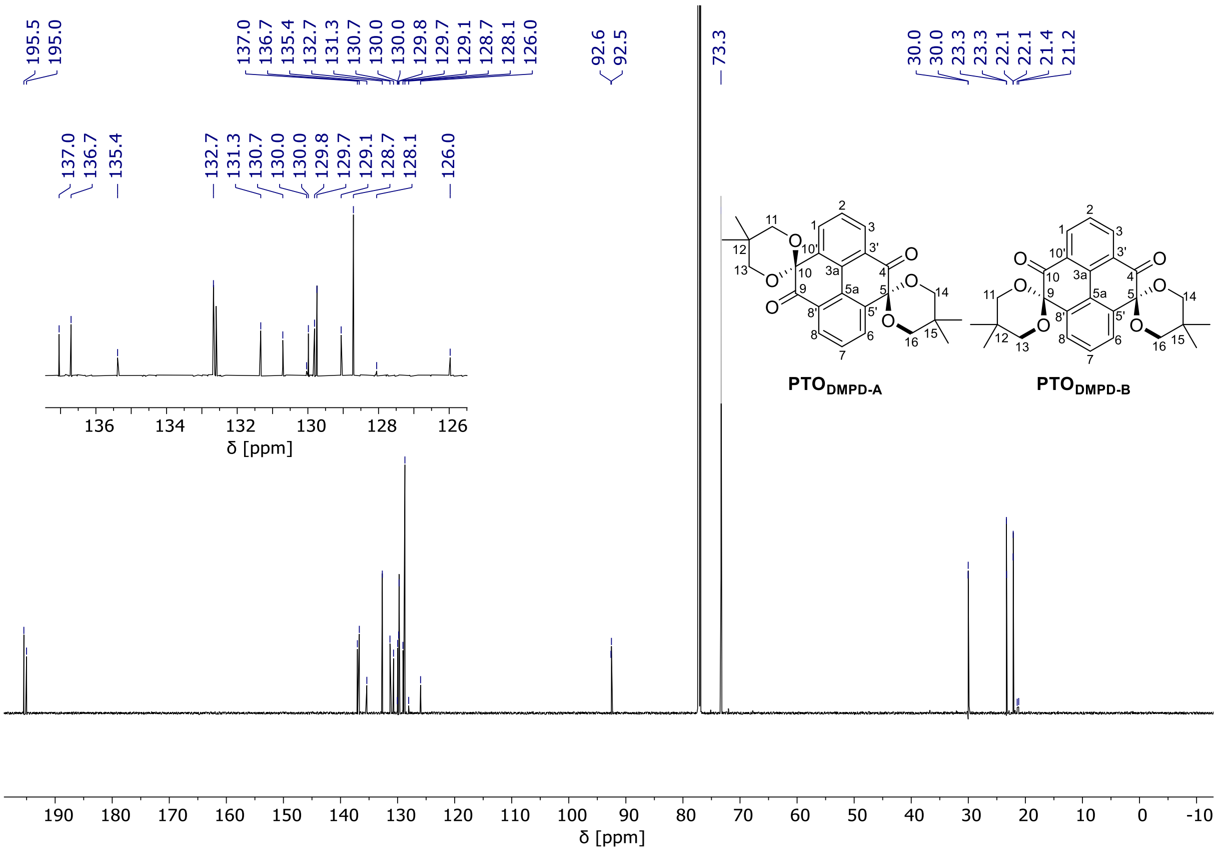

Figure S26. ^{13}C NMR spectrum (175 MHz, $CDCl_3$, 25 °C) of isomeric mixture **PTO$_{DMPD-A/B}$**.

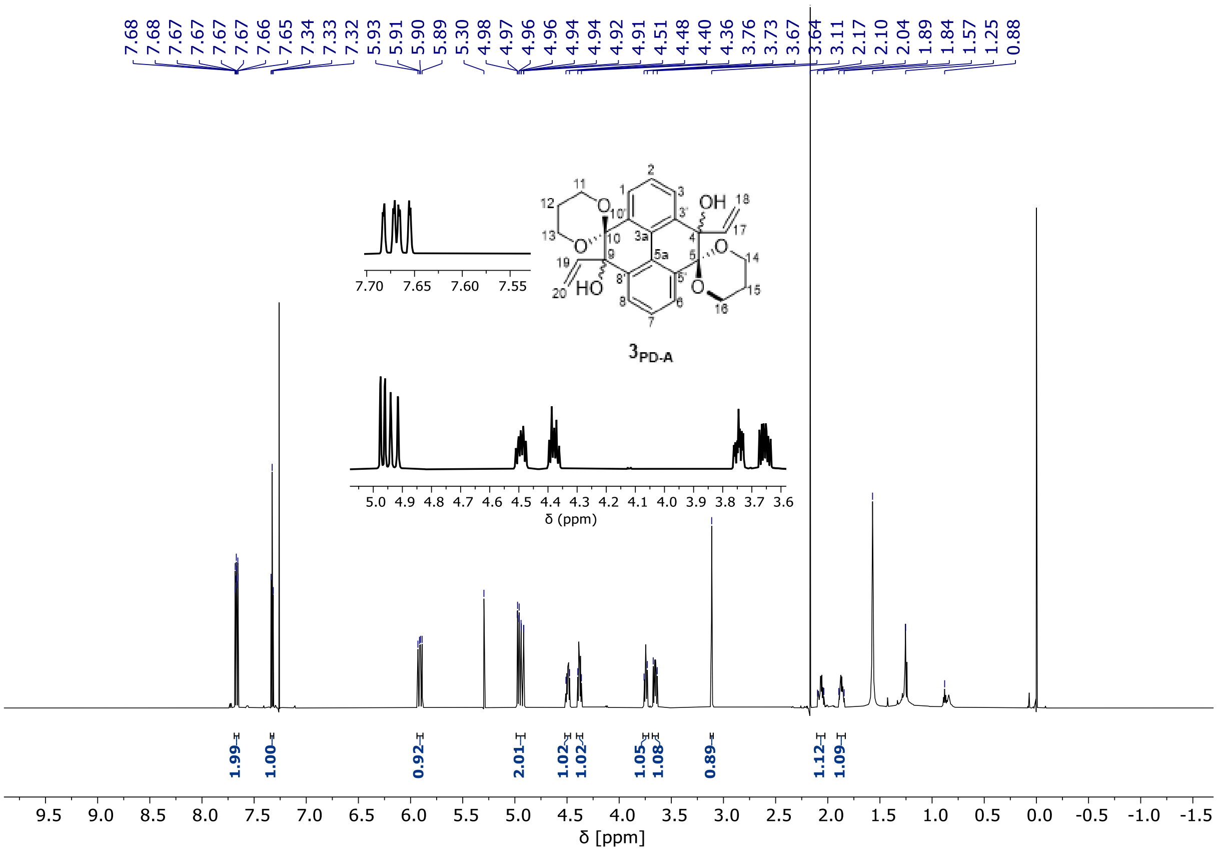

Figure S27. ^{1}H NMR spectrum (700 MHz, $CDCl_3$, 25 °C) of **3$_{PD-A}$**.

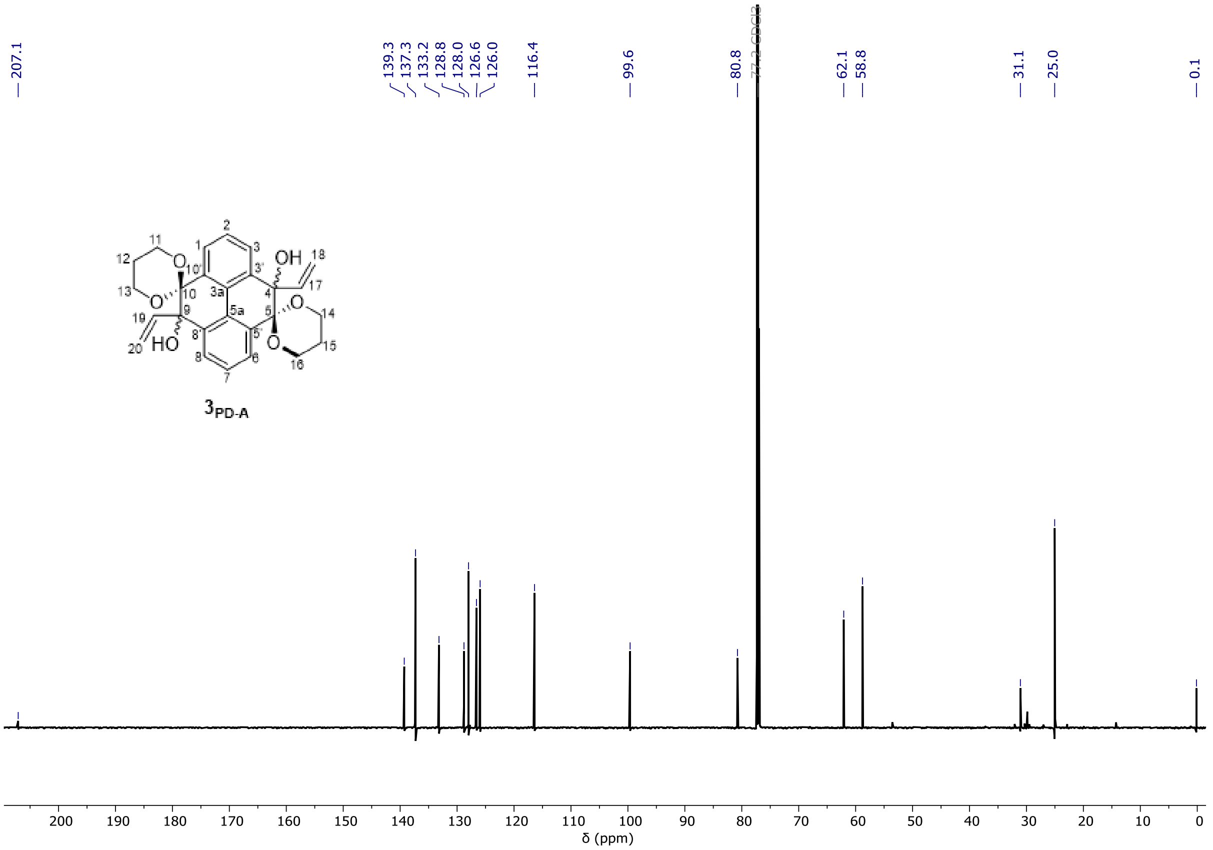

Figure S28. ^{13}C NMR spectrum (175 MHz, $CDCl_3$, 25 °C) of **3$_{PD-A}$**.

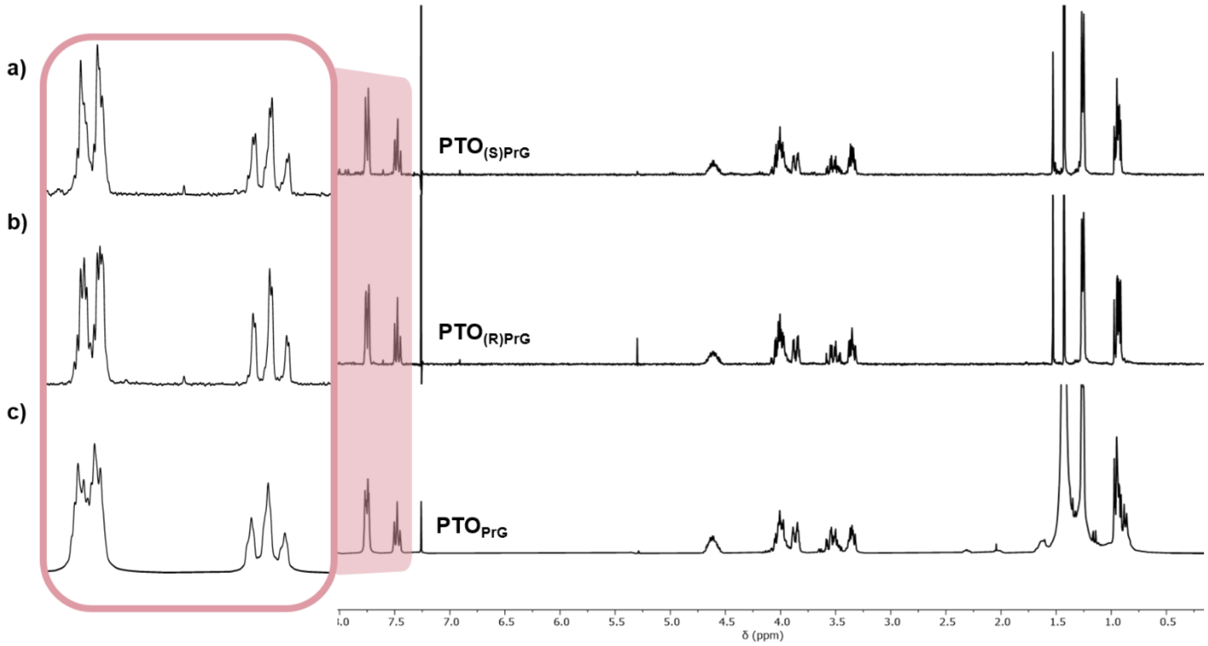

Figure S29. ^{1}H NMR spectrum (300 MHz, $CDCl_3$, 25 °C) of a) **PTO$_{(S)PrG}$** b) **PTO$_{(R)PrG}$** and c) racemic **PTO$_{PrG}$**.

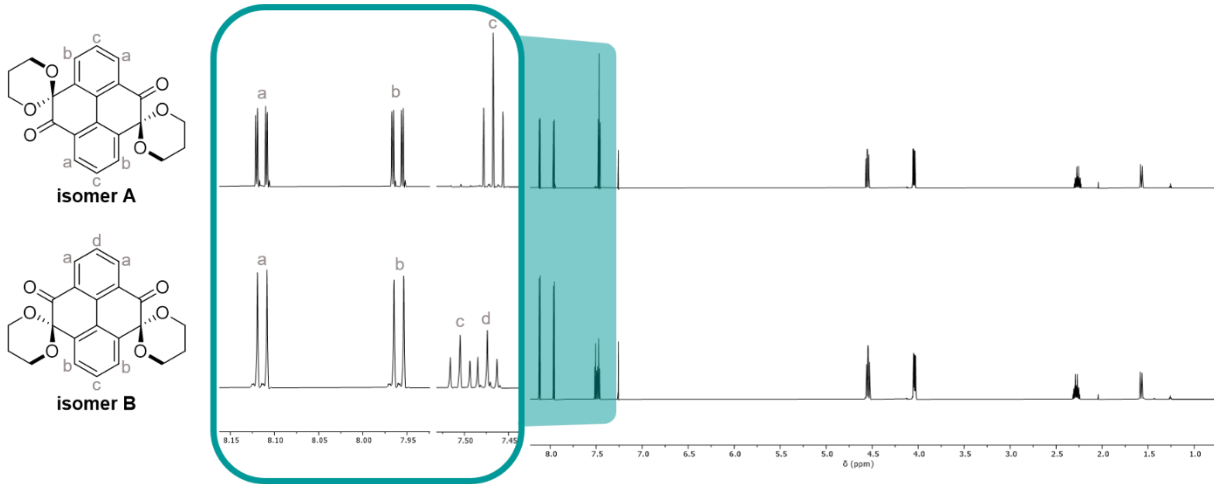

Figure S30. ^{1}H NMR spectrum (700 MHz, $CDCl_3$, 25 °C) of **PTO$_{PD-A}$** (top) and **PTO$_{PD-B}$** (bottom).

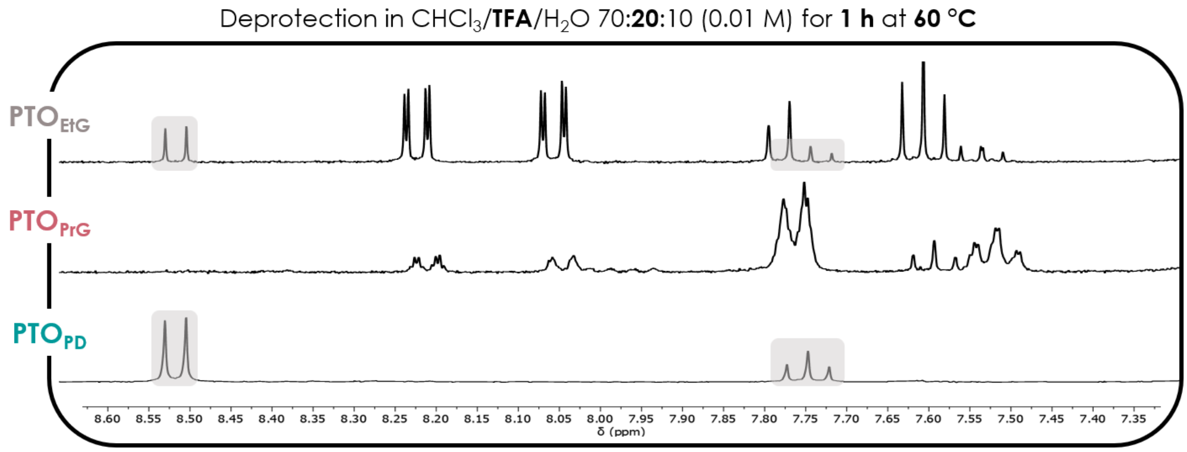

Figure S31. Comparison of deprotection between **PTO_{EtG}**, **PTO_{PrG}**, and **$PTO_{PD-A/B}$** (^{1}H NMR spectrum (300 MHz, 25 °C)) in $CDCl_3/TFA/H_2O$ 70:20:10 (0.01 M) for 1 h at 60 °C. Highlighted in grey are the characteristic doublet and triplet peaks of **PTO**.

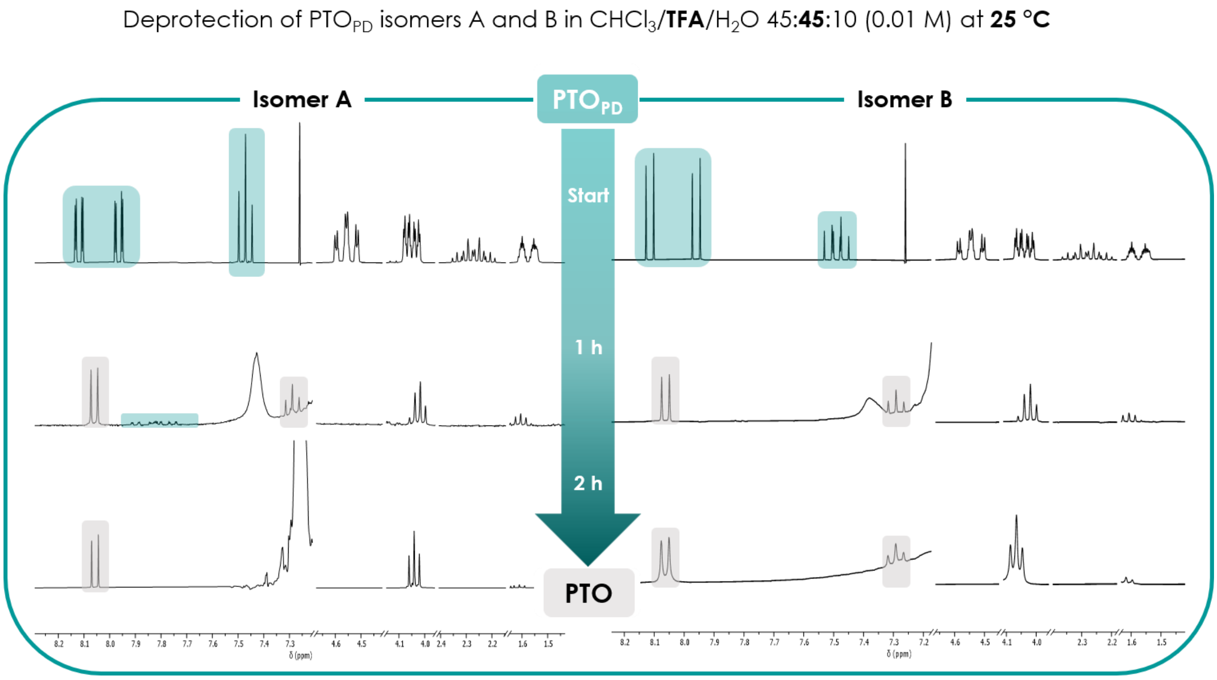

Figure S32. Comparison of deprotection between **PTO_{PD-A}** and **PTO_{PD-B}** (^{1}H NMR spectrum (300 MHz, 25 °C)) in $CDCl_3/TFA/H_2O$ 45:45:10 (0.01 M) over 2 h at 25 °C. Highlighted in blue are the characteristic doublet and triplet peaks of the **$PTO_{PD-A/B}$** isomers; and in grey, the characteristic doublet and triplet peaks of **PTO**.

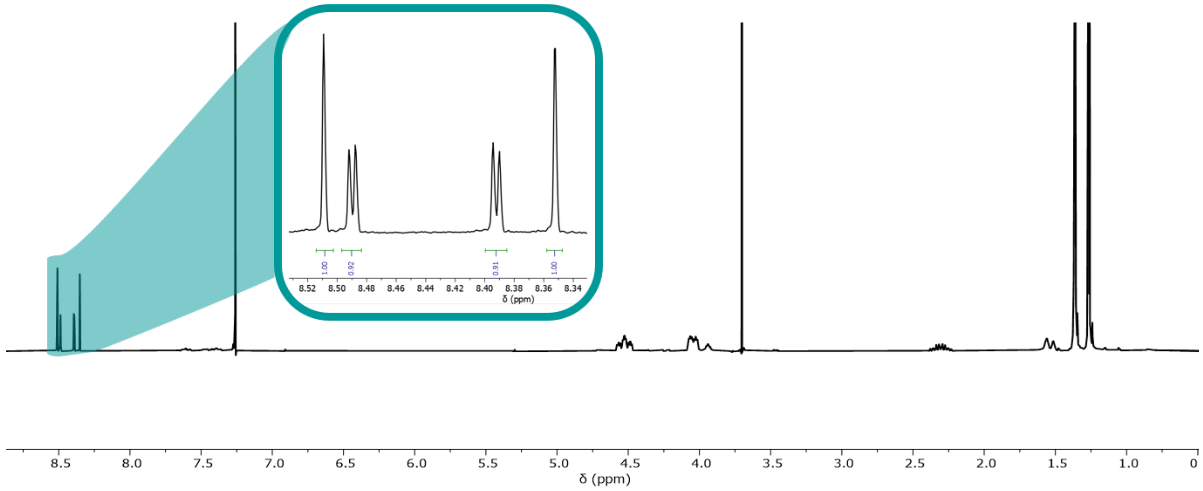

Figure S33. Crude ^{1}H NMR spectra (300 MHz, $CDCl_3$, 25 °C) of **1**$_{\mathbf{PD\text{-}A/B}}$, illustrating the 1:1 ratio between **1**$_{\mathbf{PD\text{-}A}}$ and **1**$_{\mathbf{PD\text{-}B}}$.

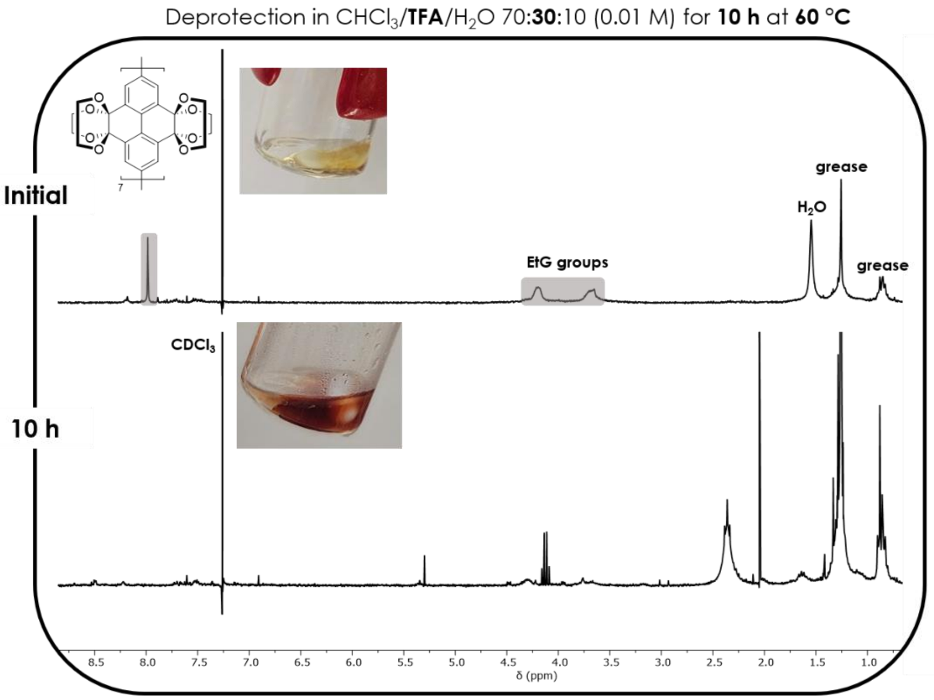

Figure S34. ^{1}H NMR spectrum (300 MHz, $CDCl_3$, 25 °C) of unsuccessful deprotection of previously synthesized [7] 7-fold **PTO**$_{\mathbf{EtG}}$ strained aromatic macrocycle. Deprotection conditions used are the mildest successful conditions found for monomeric **PTO**$_{\mathbf{EtG}}$, and yet, these conditions clearly lead to complete decomposition of the strained macrocycle, with no distinct aromatic peaks found. Reaction mixture after deprotection also contains black insoluble powder which we believe is decomposed **PTO** compounds.

S5. Mass Spectra

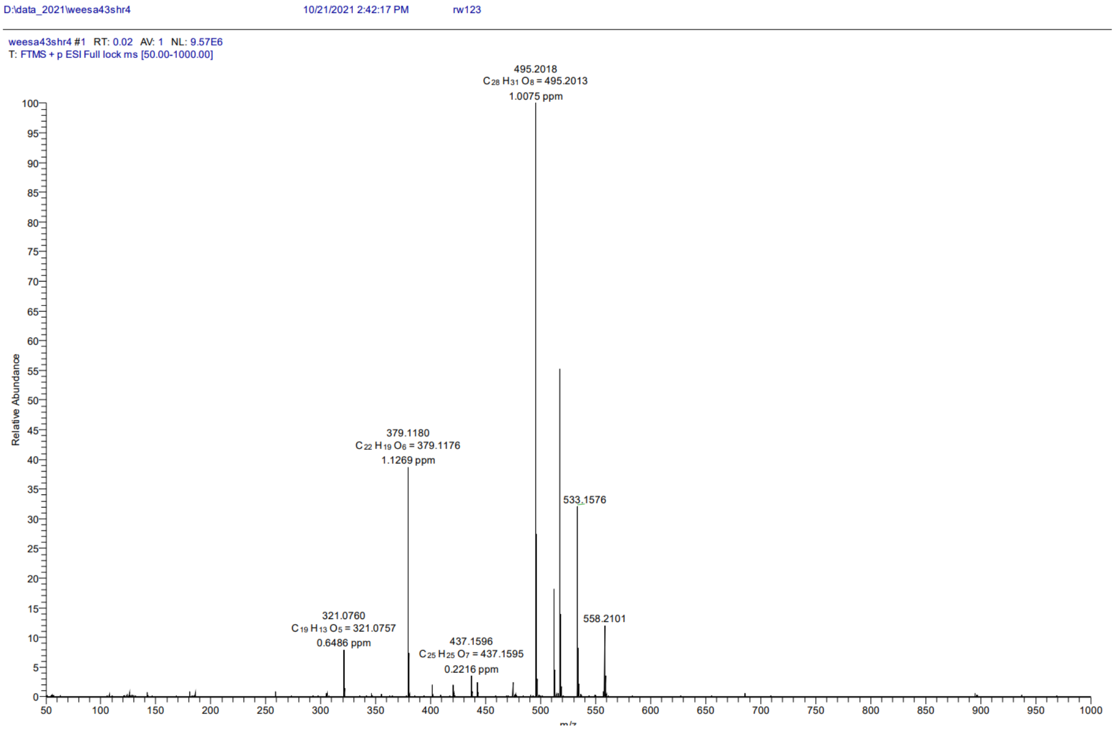

Figure S35. HRMS spectrum of racemic **PTO$_{PrG}$.**

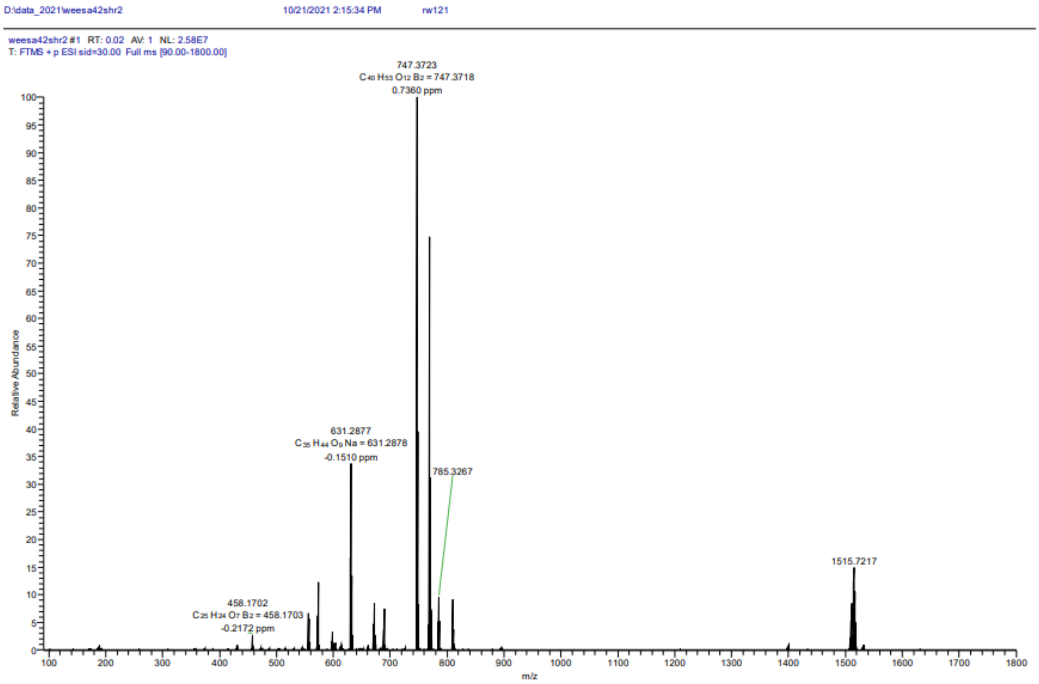

Figure S36. HRMS spectrum of **1$_{PrG}$.**

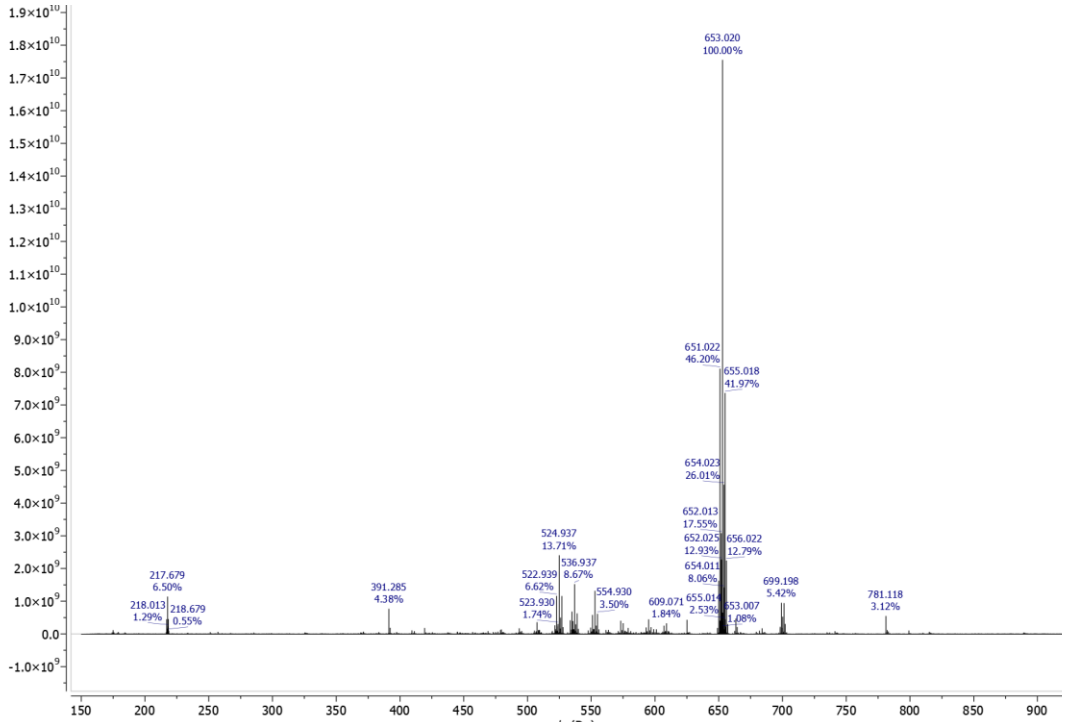

Figure S37. HRMS spectrum of **2$_{PrG}$**.

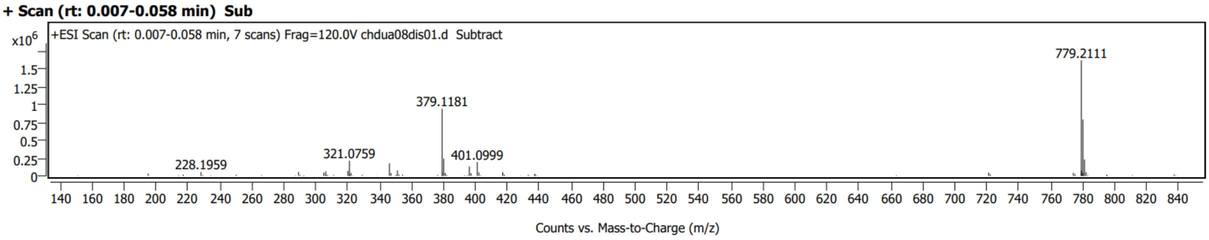

Figure S38. HRMS spectrum of **PTO$_{PD-A/B}$.**

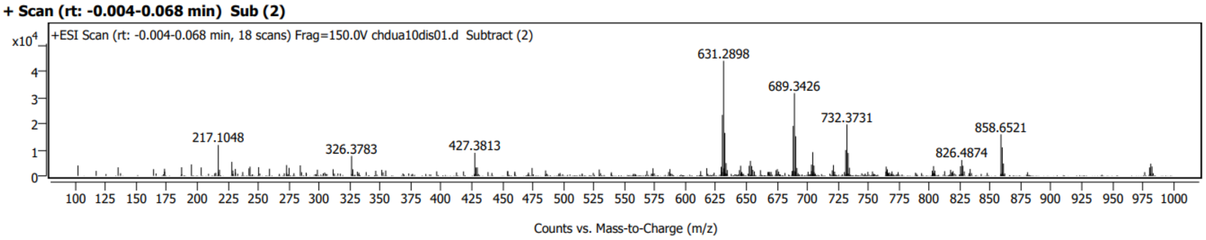

Figure S39. HRMS spectrum of **1$_{PD}$**.

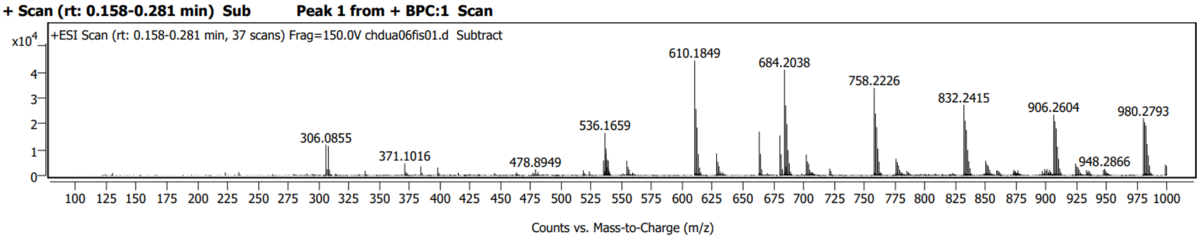

Figure S40. HRMS spectrum of $\mathbf{2}_{\mathbf{PD}}$.

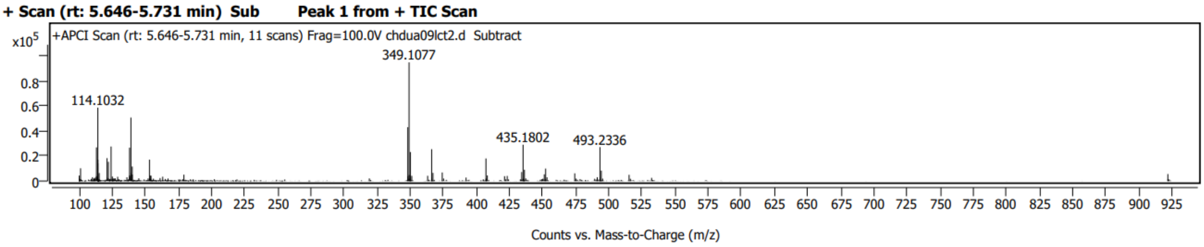

Figure S41. HRMS spectrum of $\mathbf{PTO}_{\mathbf{DMPD}}$.

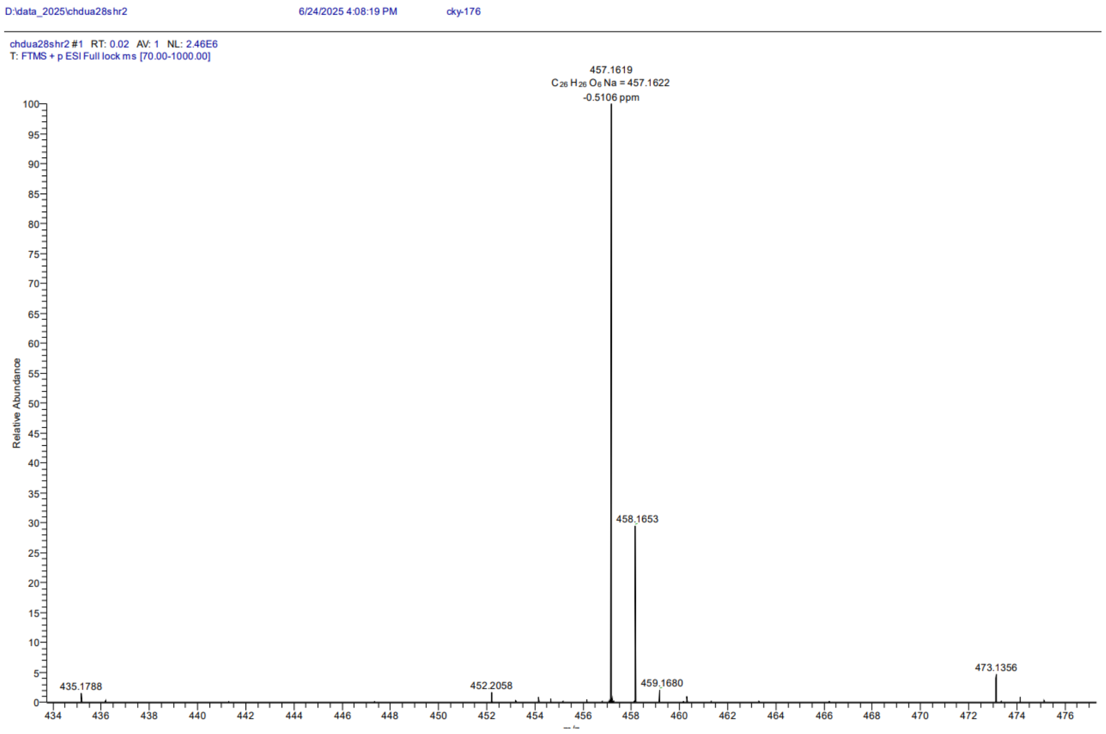

Figure S42. HRMS spectrum of $\mathbf{3}_{\mathbf{PD\text{-}A}}$.

S6. Infrared Spectroscopy

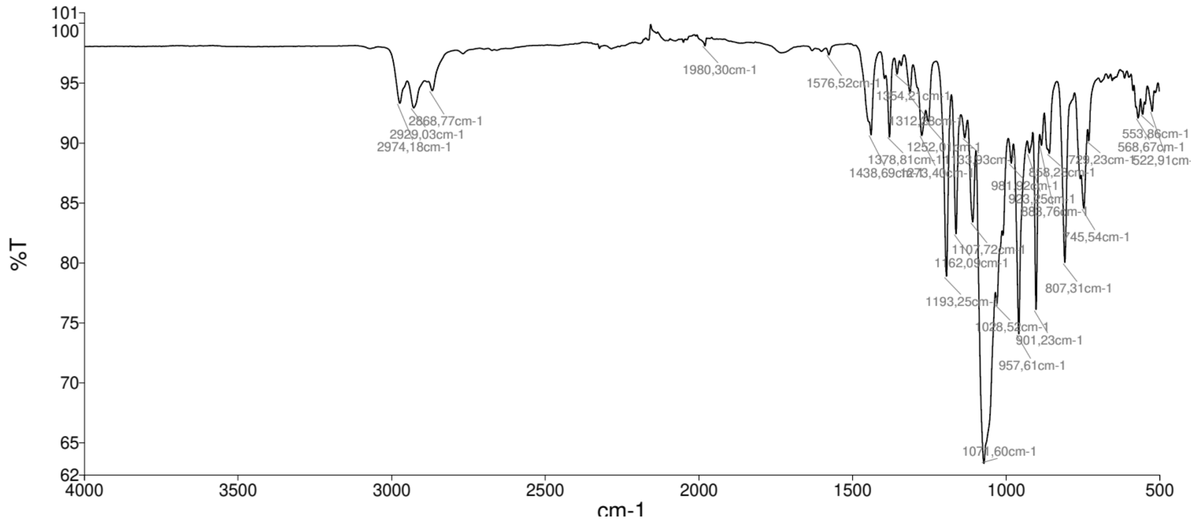

Figure S43. IR spectrum of $\mathbf{PTO_{PrG}}$.

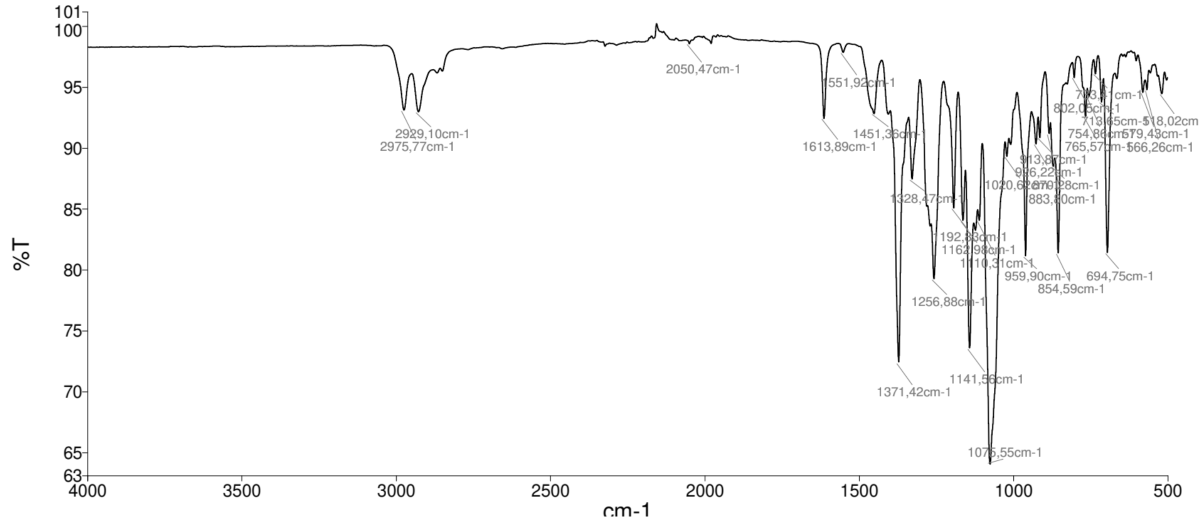

Figure S44. IR spectrum of $\mathbf{1_{PrG}}$.

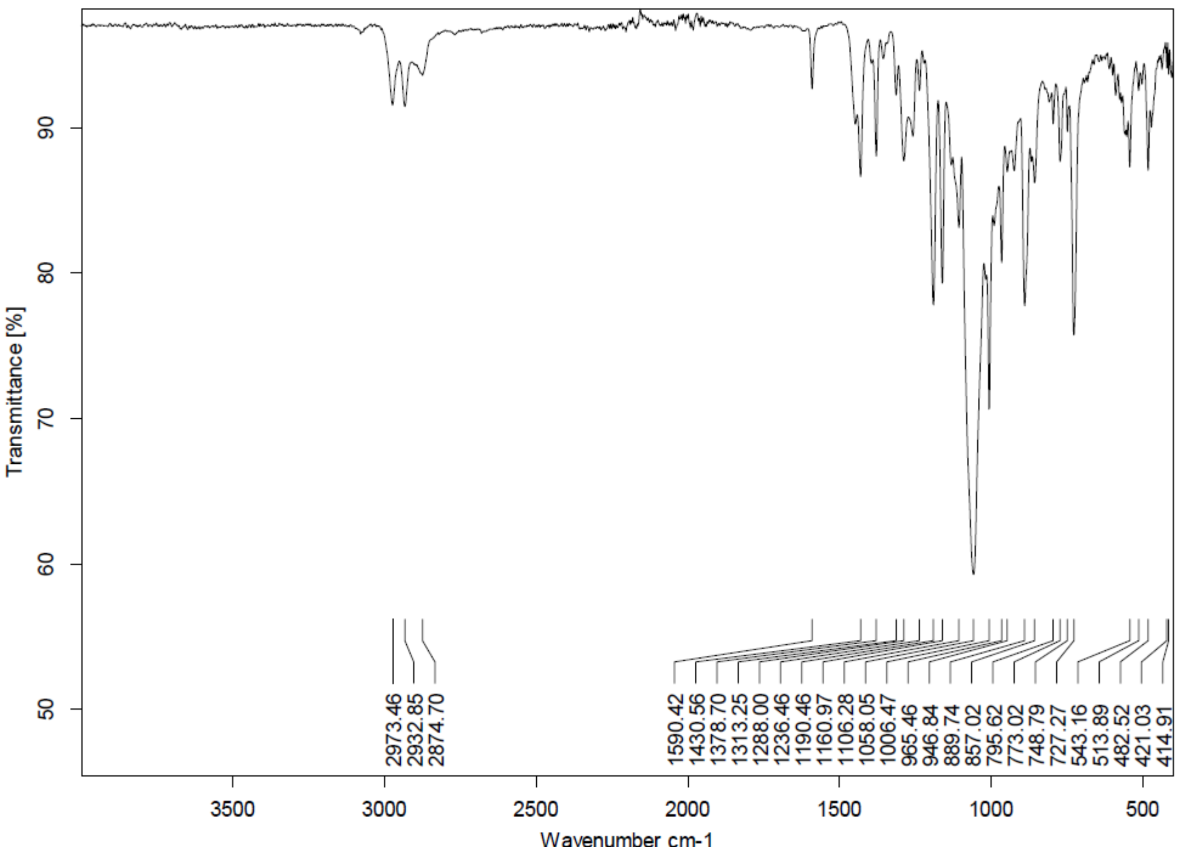

Figure S45. IR spectrum of **2_{PrG}**.

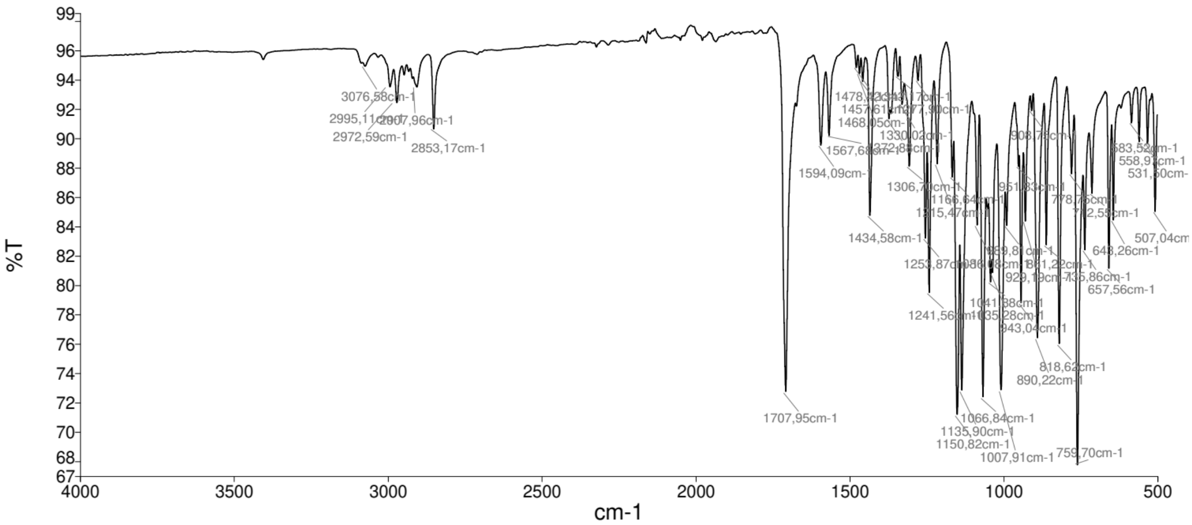

Figure S46. IR spectrum of **$PTO_{PD-A/B}$**.

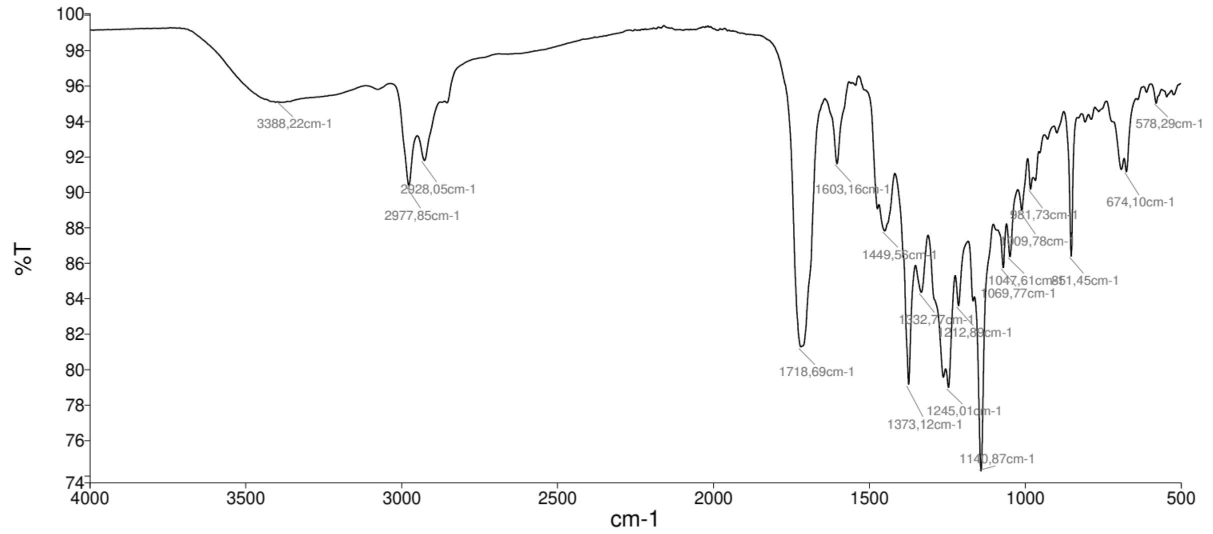

Figure S47. IR spectrum of $\mathbf{1_{PD\text{-}A/B}}$.

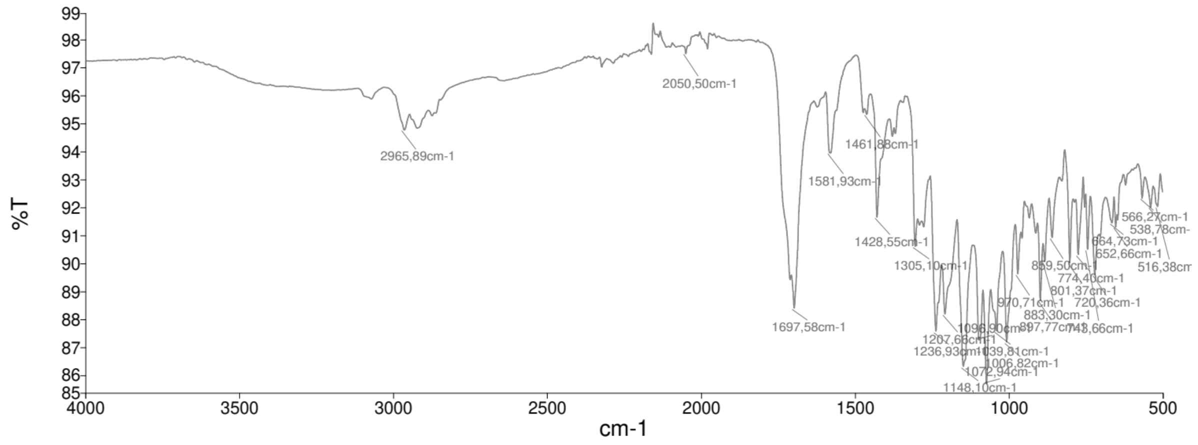

Figure S48. IR spectrum of $\mathbf{2_{PD\text{-}A/B}}$.

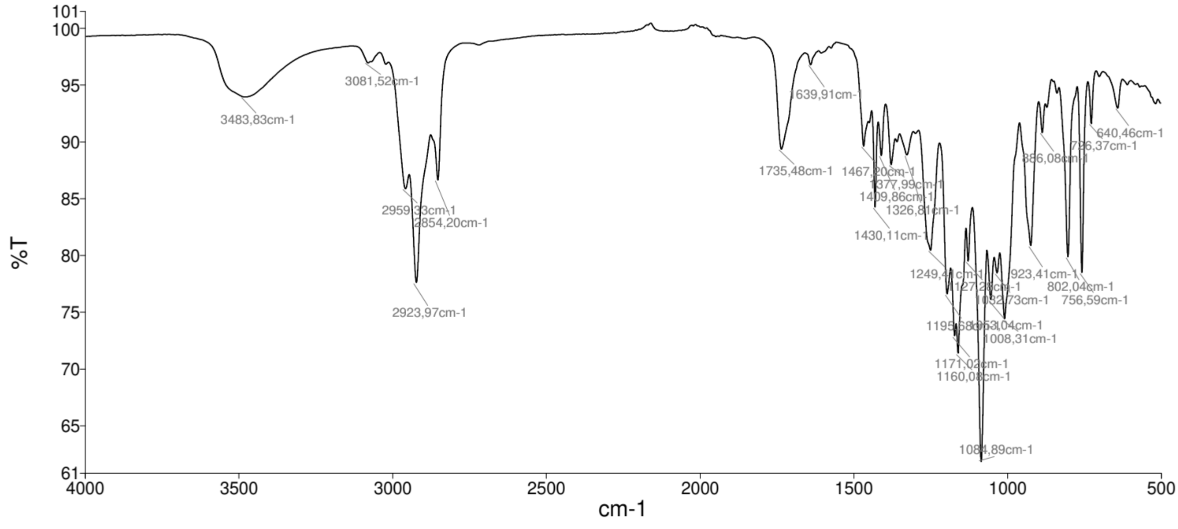

Figure S49. IR spectrum of $\mathbf{3_{PD\text{-}A}}$.

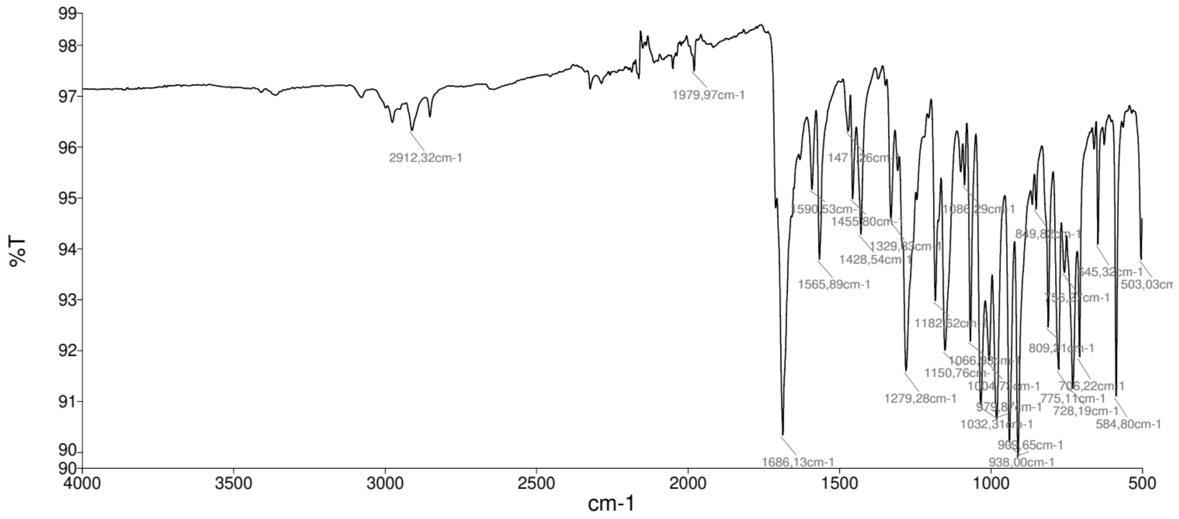

Figure S50. IR spectrum of **$PTO_{DMPD-A/B}$.**

S7. Attempts Towards Alternative PTO Protecting Groups

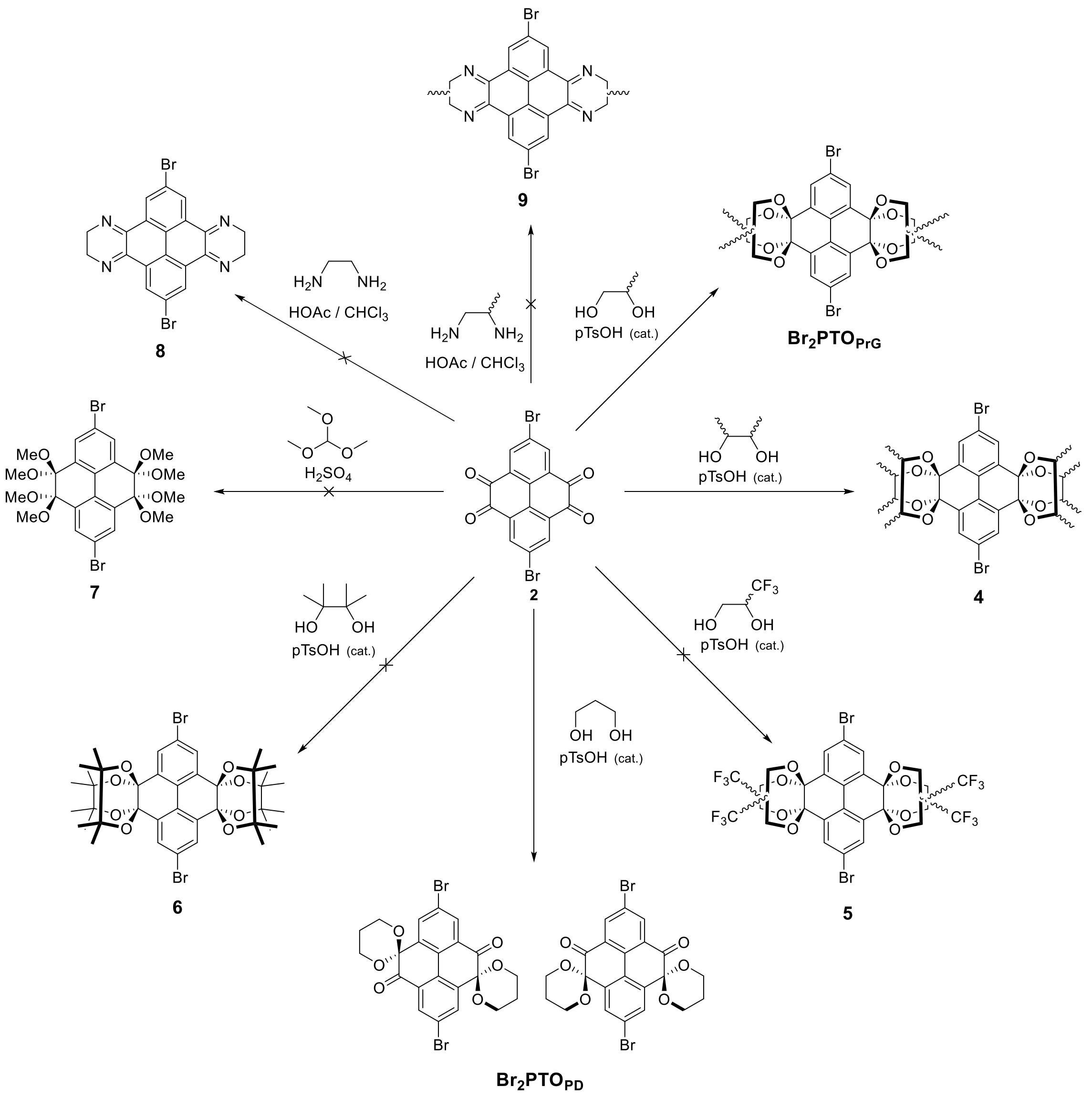

Scheme S1. Attempts towards alternative protecting groups for **PTO**.

Table S1. Overview of attempts towards alternative **PTO** protecting groups.

Compound	Result
**4**	Target successfully synthesized
**5**	Starting material decomposed. Protecting group is volatile and likely distilled over Dean-Stark trap while refluxing
**6**	Reaction mixture turned black during reflux, indicating decomposition
**7**	Mainly starting material. No relevant NMR signals found for target
**8**	Mainly starting material. No relevant NMR signals found for target
**9**	Mainly starting material. No relevant NMR signals found for target

Experimental Procedures of Alternative Protecting Groups Strategies

Syntheses of compounds **4**, **5**, and **6** were conducted according to General Procedure A. Synthesis of compound **7** was conducted following a modified procedure from the literature.[9] Syntheses of compounds **8** and **9** were conducted according to General Procedure B, a modified procedure from the literature.[10]

General procedure A.

Protected **PTO**s were synthesized following a modified procedure that was earlier reported for EtG protections by MERZ *et al.*[6] Dibromo-**PTO 2** (1.00 g, 3.81 mmol), *p*-toluenesulfonic acid hydrate (653 mg, 3.43 mmol, 0.90 equiv.) and corresponding diol (250 equiv.) were added to toluene (150 mL) and refluxed for 15 h in an oil bath using a DEAN-STARK apparatus. After cooling to room temperature, the reaction mixture was washed with H_2O (2 × 100 mL) and brine (100 mL). The combined aqueous phase was extracted with toluene (2 × 75 mL). The combined organic phase was dried over Na_2SO_4, the volatiles were removed under reduced pressure and the crude product was purified using flash column chromatography (SiO_2, *n*-hexane/ethyl acetate).

Compound **4**: **2** (200 mg, 0.476 mmol) and butane-2,3-diol (10.6 mL, 119 mmol, 250 equiv.) in toluene (16 mL) gave target compound.

Compound **5**: **2** (100 mg, 0.238 mmol) and 3,3,3-trifluoropropane-1,2-diol (5.35 mL, 59.5 mmol, 250 equiv.) in toluene (8 mL) gave black decomposed material.

Compound **6**: **2** (200 mg, 0.476 mmol) and 2,3-dimethylbutane-2,3-diol (1.125 g, 9.52 mmol, 20 equiv.) in toluene (16 mL) gave black decomposed material.

Synthesis of compound **7** was conducted following a modified procedure from the literature.[9] Concentrated sulfuric acid (0.01 ml) was added to a stirred solution of **2** (200 mg, 0.476 mmol) in methanol (2 mL) and trimethoxymethane (3.2 mL, 0.290 mmol, 61 equiv.). The reaction mixture was heated under reflux in an oil bath for 15 h and then neutralized with sodium hydrogen carbonate. The solvent was removed under reduced pressure to give mainly starting material.

General procedure B.

Protected **PTO**s were synthesized following a modified procedure by MASTALERZ *et al.*[10] Pyrene-4,5,9,10-tetraone (**PTO**, 200 mg, 0.476 mmol) and diamine (2.05 equiv.) were suspended in chloroform (10 mL), glacial acetic acid (2.5 mL) and heated under argon at 70 °C in an oil bath for 16 h. After cooling to 25 °C, the yellow suspension was filtered and the precipitate was washed with methanol and hot chloroform. Neither the precipitate nor filtrate gave relevant signals for target compound.

Compound **8**: **PTO** (200 mg, 0.476 mmol) and ethane-1,2-diamine (58.7 mg, 0.976 mmol, 2.05 equiv.) gave mainly starting material.

Compound **9**: **PTO** (100 mg, 0.238 mmol) and propane-1,2-diamine (0.083 mL, 0.976 mmol, 2.05 equiv.) gave mainly starting material.

Crude ^{1}H NMRs of Alternative PTO Protecting Group Strategies

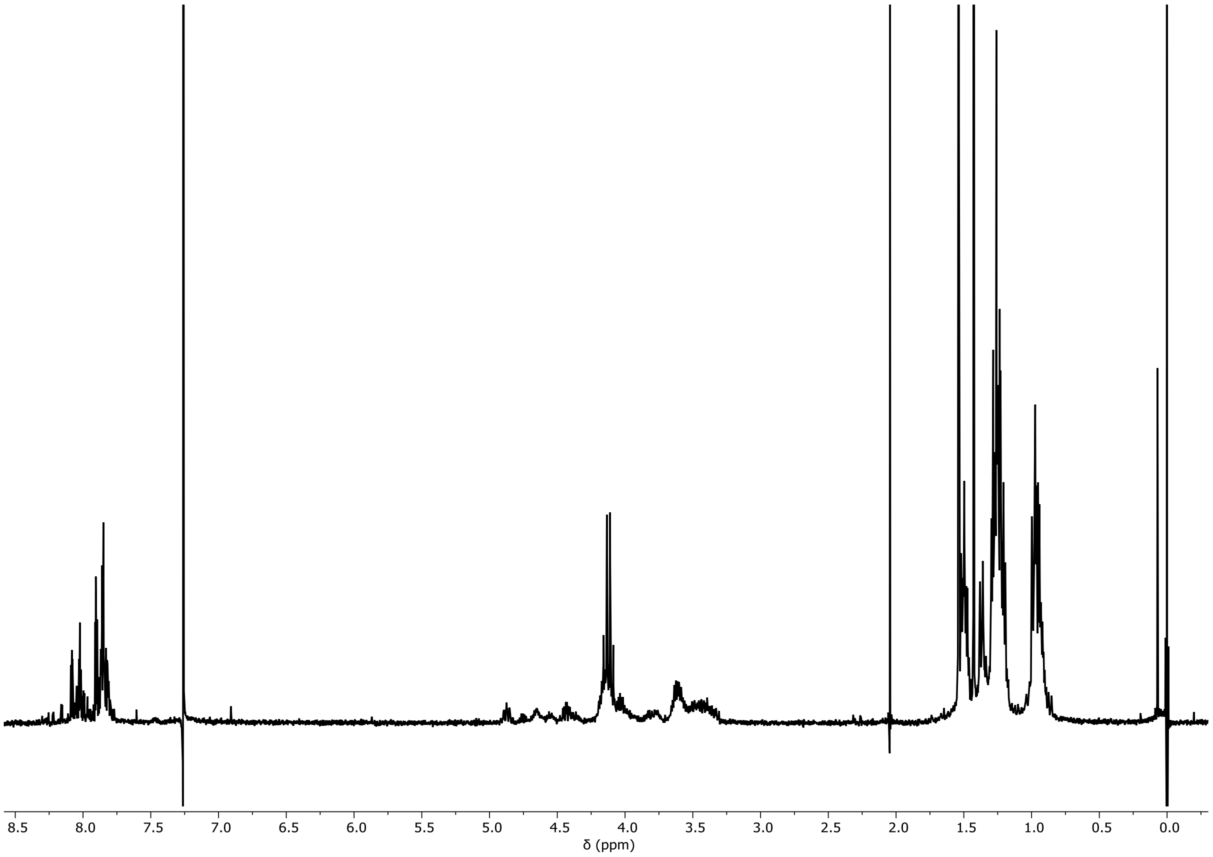

Figure S51. ^{1}H NMR spectrum (300 MHz, $CDCl_3$, 25 °C) of crude compound **4**.

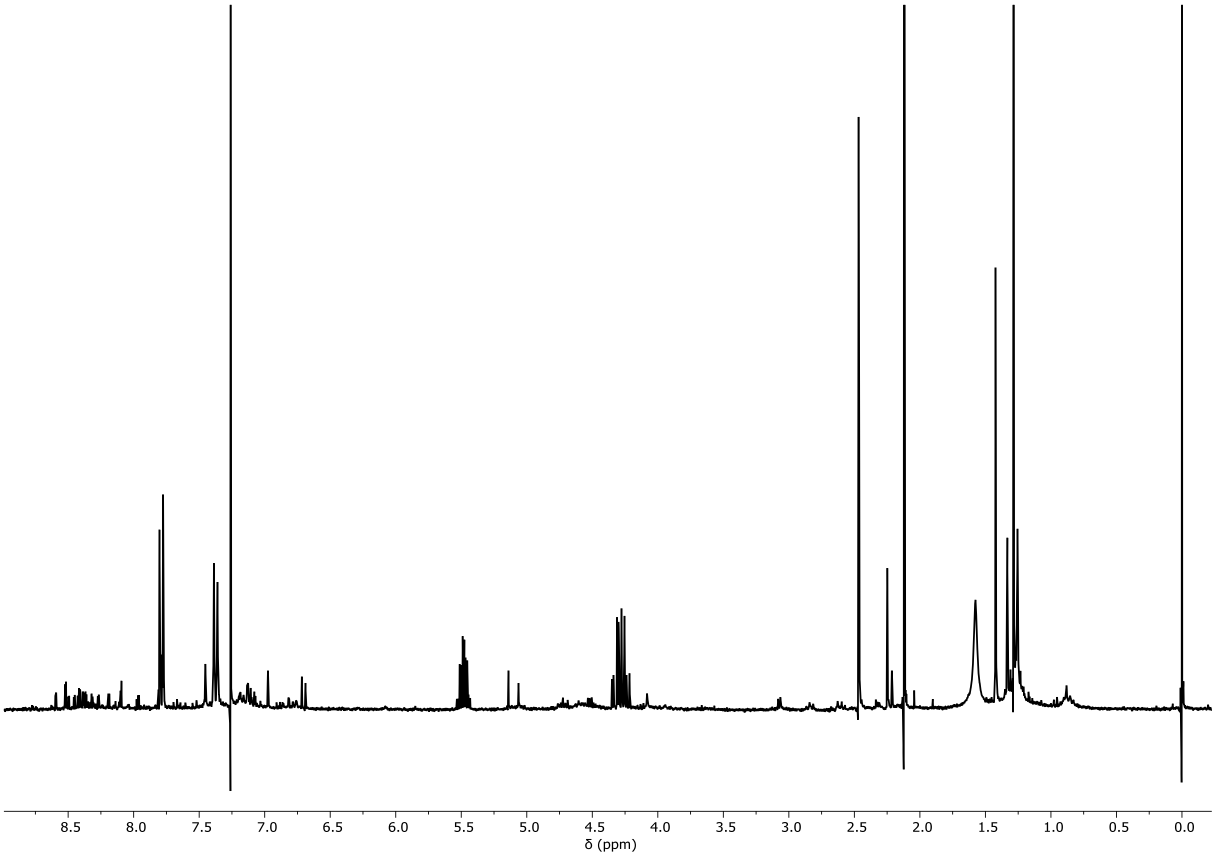

Figure S52. ^{1}H NMR spectrum (300 MHz, $CDCl_3$, 25 °C) of crude product mixture for the synthesis attempt of compound **5**.

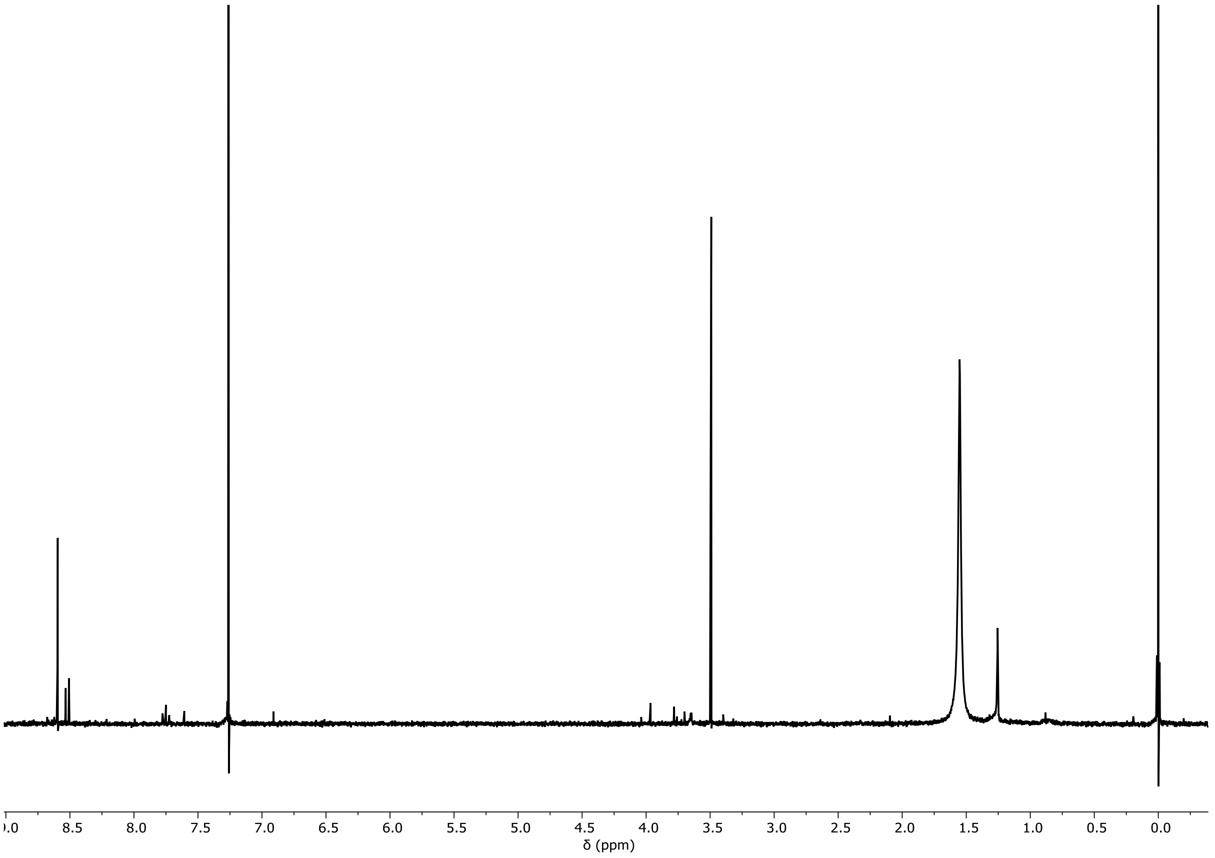

Figure S53. ^{1}H NMR spectrum (300 MHz, $CDCl_3$, 25 °C) of crude product mixture for the synthesis attempt of compound **7**.

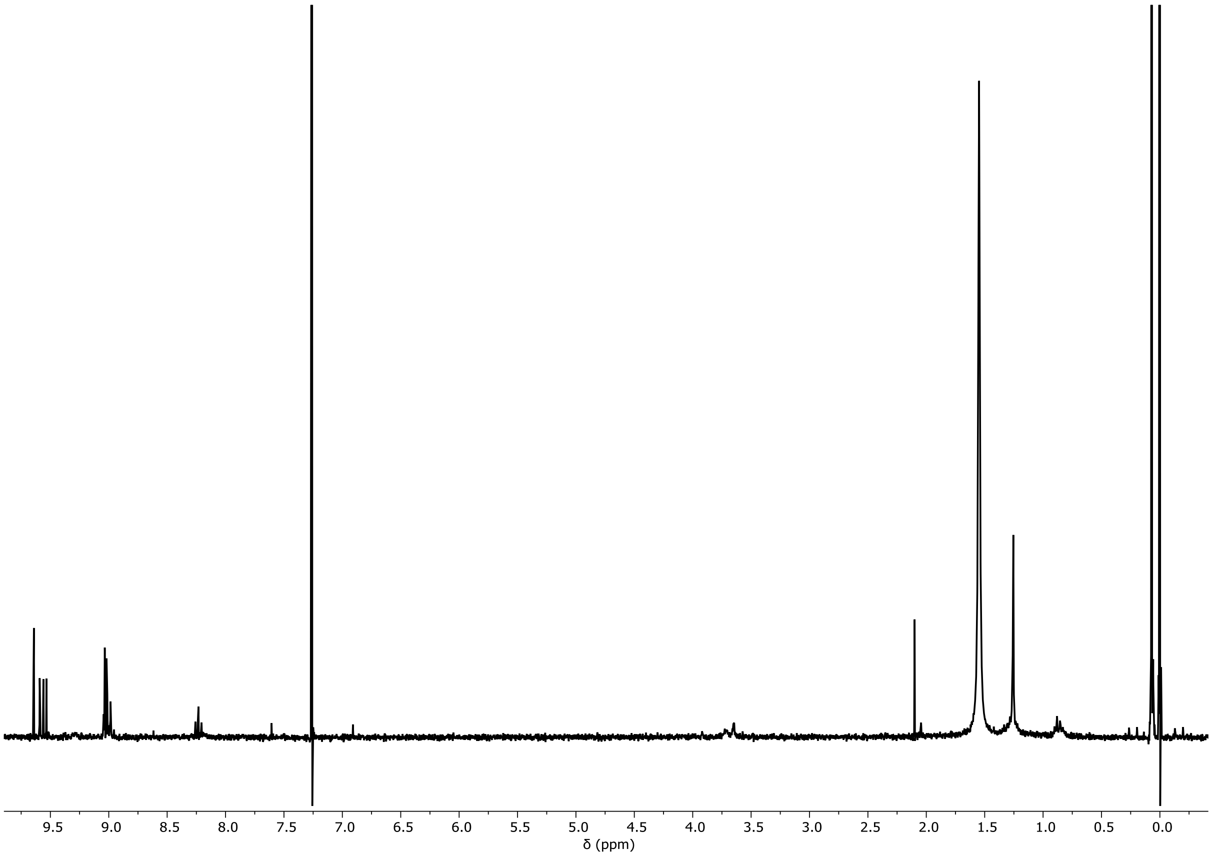

Figure S54. ^{1}H NMR spectrum (300 MHz, $CDCl_3$, 25 °C) of crude product mixture for the synthesis attempt of compound **8**.

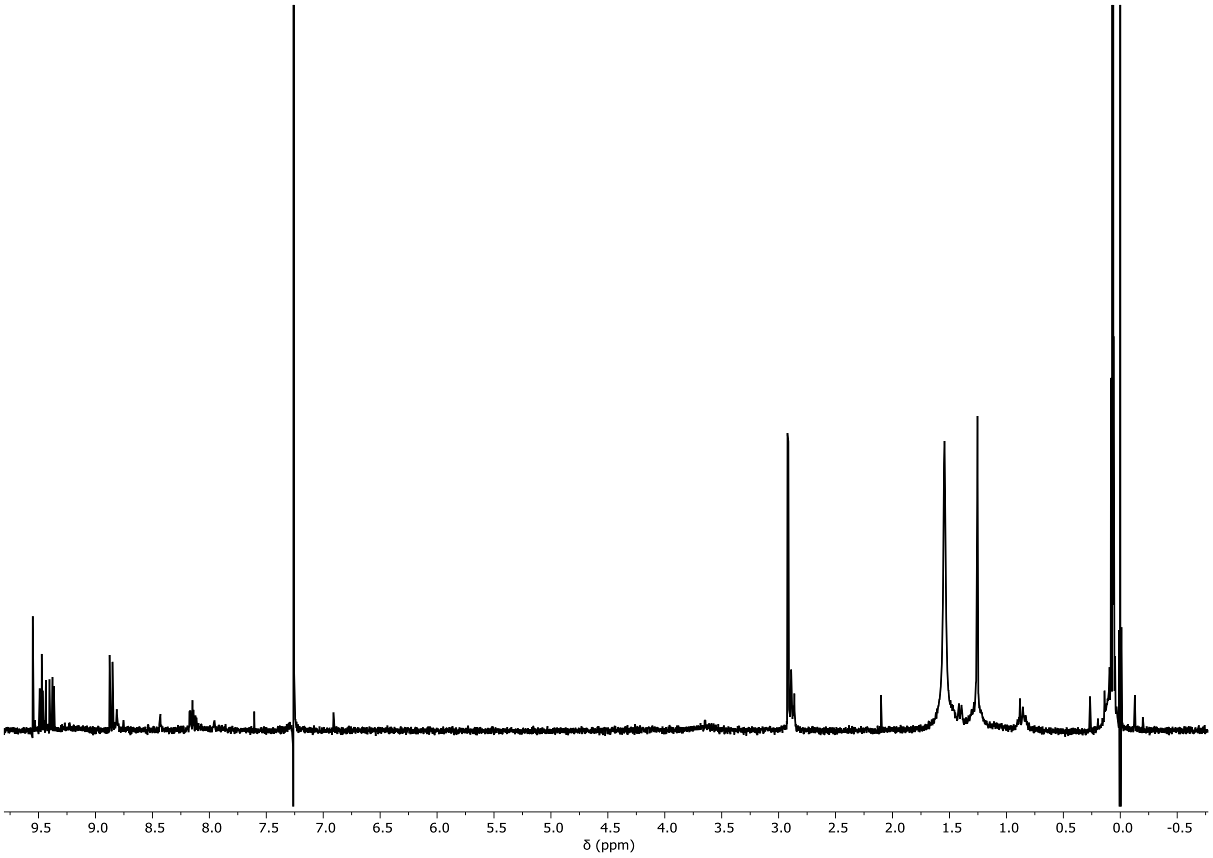

Figure S55. ^{1}H NMR spectrum (300 MHz, $CDCl_3$, 25 °C) of crude product mixture for the synthesis attempt of compound **9**.

S8. Single-Crystal X-Ray Data

X-ray Crystallography. CCDC-2430035 (**PTO$_{PD-A}$**) and CCDC-2384412 (**2$_{PD-B}$**) contain the supplementary crystallographic data for this paper, including structure factors and refinement instructions. These data can be obtained free of charge from the joint Cambridge Crystallographic Data Centre and Fachinformationszentrum Karlsruhe Access Structures service (Cambridge Crystallographic Data Centre, 12 Union Road, Cambridge CB2 1EZ, UK (fax: +44(1223)-336-033; e-mail: deposit@ccdc.cam.ac.uk), or online via www.ccdc.cam.ac.uk/structures.

Single-crystal X-ray Data and Structure Analysis for PTO$_{PD-A}$

Colorless crystalline needles were grown by recrystallization from hot ethyl acetate upon slow cooling and evaporation.

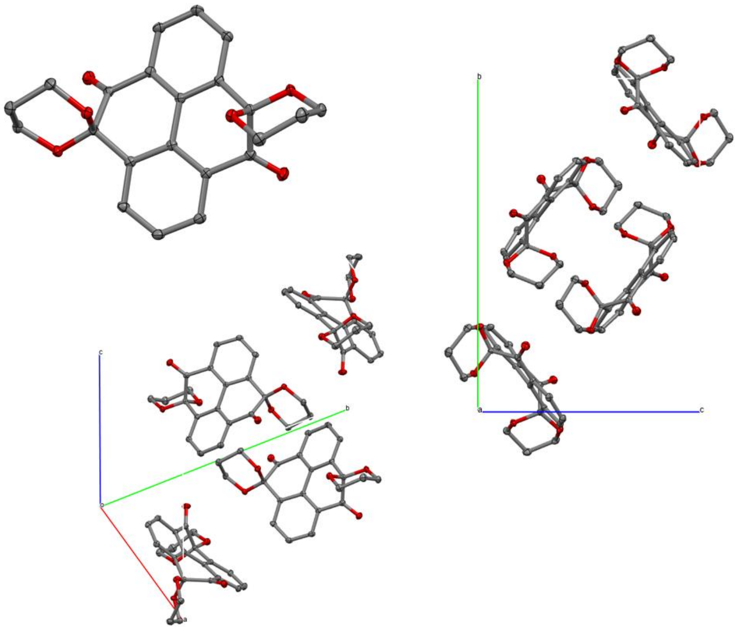

Figure S56. X-ray structure of **PTO$_{PD-A}$** at 100 K. ORTEP representation of as thermal ellipsoids in 50% probability. Hydrogen atoms are omitted for clarity. Color code: carbon (grey) and oxygen (red).

Table S2. Crystal data and structure refinement for **PTO_{PD-A}**

CCDC Deposition number	2430035
Empirical formula	$C_{22}H_{18}O_6$
Formula weight	378.36
Temperature/K	100.00
Crystal system	monoclinic
Space group	$P2_1/n$
a/Å	9.3195(8)
b/Å	16.5063(13)
c/Å	11.3029(9)
α/°	90
β/°	103.373(3)
γ/°	90
Volume/Å^3	1691.6(2)
Z	4
ρ_{calc}g/cm^3	1.486
μ/mm^{-1}	0.109
F(000)	792.0
Crystal size/mm^3	0.326 × 0.261 × 0.102
Radiation	MoKα (λ = 0.71073)
2Θ range for data collection/°	4.45 to 56.742
Index ranges	$-12 \le h \le 12$, $-22 \le k \le 22$, $-15 \le l \le 15$
Reflections collected	88788
Independent reflections	4209 [R_{int} = 0.0417, R_{sigma} = 0.0128]
Data/restraints/parameters	4209/0/253
Goodness-of-fit on F^2	1.056
Final R indexes [I>=2σ (I)]	R_1 = 0.0363, wR_2 = 0.0966
Final R indexes [all data]	R_1 = 0.0376, wR_2 = 0.0977
Largest diff. peak/hole / e Å^{-3}	0.49/-0.23

Single-crystal X-ray Data and Structure Analysis for 2_{PD-B}

Yellow crystalline blocks were grown via layered diffusion of methanol into a toluene solution of the compound.

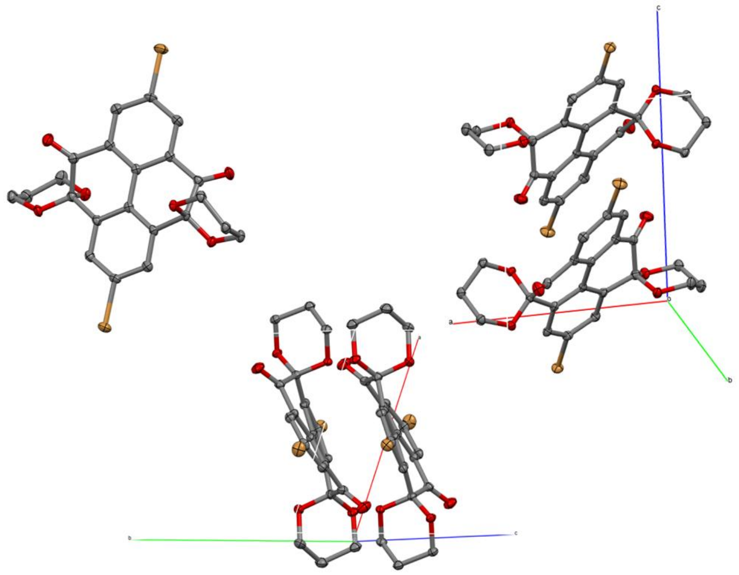

Figure S57. X-ray structure of **2_{PD-B}** at 100 K. ORTEP representation of as thermal ellipsoids in 50% probability. Hydrogen atoms are omitted for clarity. Color code: carbon (grey), oxygen (red) and bromine (brown).

Table S3. Crystal data and structure refinement for **2_{PD-B}**

Identification code	2384412
Empirical formula	$C_{22}H_{16}Br_2O_6$
Formula weight	1072.33
Temperature/K	100.00
Crystal system	triclinic
Space group	*P*-1
a/Å	9.1357(3)
b/Å	9.7781(3)
c/Å	12.0585(4)
α/°	101.609(2)
β/°	95.604(2)
γ/°	112.373(2)
Volume/Å^3	957.50(6)
Z	2
ρ_{calc}g/cm^3	1.860
μ/mm^{-1}	4.273
F(000)	532.0
Crystal size/mm^3	0.435 × 0.218 × 0.17
Radiation	MoKα (λ = 0.71073)
2Θ range for data collection/°	3.514 to 56.664

Index ranges	$-12 \le h \le 12$, $-13 \le k \le 13$, $-16 \le l \le 16$
Reflections collected	63225
Independent reflections	4771 [R_{int} = 0.0414, R_{sigma} = 0.0166]
Data/restraints/parameters	4771/0/271
Goodness-of-fit on F^2	1.052
Final R indexes [I>=2σ (I)]	R_1 = 0.0349, wR_2 = 0.0852
Final R indexes [all data]	R_1 = 0.0408, wR_2 = 0.0890
Largest diff. peak/hole / e Å^{-3}	1.87/-0.82

S9. UV-Vis and ECD Spectroscopy

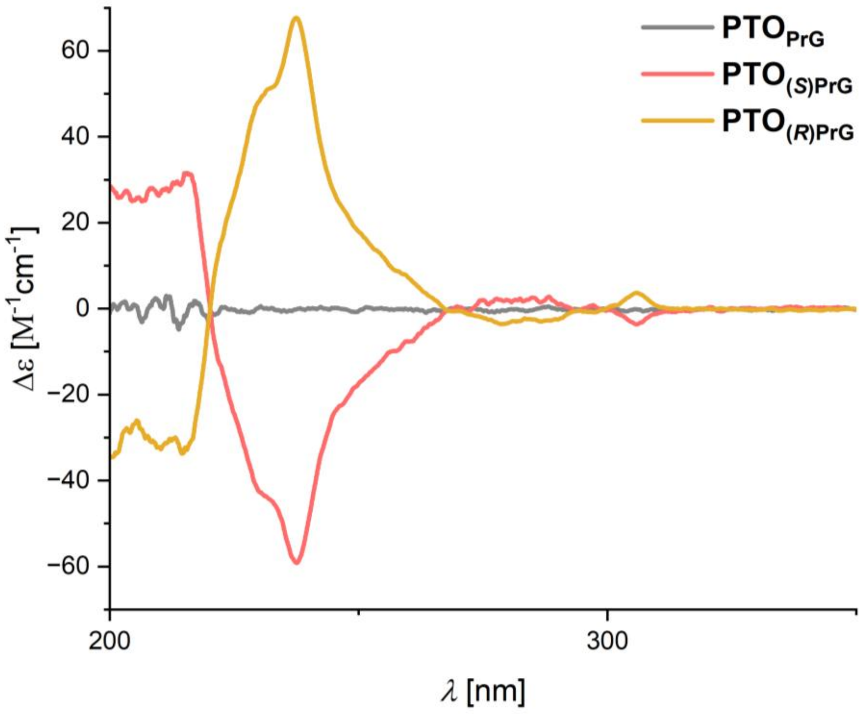

Figure S58. ECD spectra comparison of diastereomer mixture **PTO$_{(S)PrG}$** (red) and diastereomer mixture **PTO$_{(R)PrG}$** (yellow). Furthermore, the racemic mixture **PTO$_{PrG}$** (grey) is almost silent in ECD. The spectra were recorded in acetonitrile at 25 °C.

The observed ECD band from 200–220 nm can be associated with the inherent chirality from the (S)/(R) stereocentres of the propylene glycol. While the second ECD band from 220–270 nm can be associated with the K region of pyrene, chirally twisted out of plane, due to the bulky propylene glycol groups attached. **PTO$_{EtG}$** is known to possess K region twisting, as confirmed by X-ray crystallography.[7] Albeit, not as dramatically distorted when compared to **PTO$_{PrG}$** (**Figure S63**). This can be shown with the corresponding dissymmetry factor (g_{abs} factor) (**Figure S59**). At λ = 237 nm, **PTO$_{(S)PrG}$** exhibited an intense g_{abs} factor of 1.47×10^{-2}, indicating a strong chiral response.

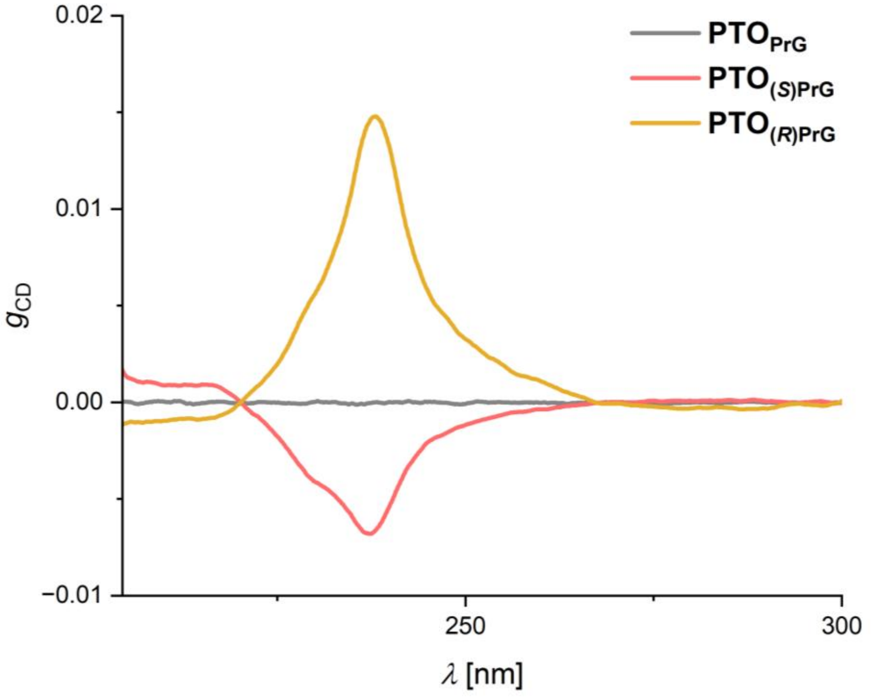

Figure S59. Calculated wavelength-dependent dissymmetry factor (g_{abs} factor) of **PTO$_{(S)PrG}$** (red), **PTO$_{(R)PrG}$** (yellow), and racemic mixture **PTO$_{PrG}$** (grey) based on **Figure S59** and **Figure S60** and equation (1). At λ = 237 nm, **PTO$_{(S)PrG}$** exhibited an intense g_{abs} factor of 1.47×10^{-2}, indicating a strong chiral response. In contrast, **PTO$_{(R)PrG}$** exhibited a g_{abs} factor of 6.78×10^{-3}, approximately two times less than the opposite homochiral analogue. This could be attributed to diastereomeric impurity of the parent propylene glycol purchased, where the (*S*)-propylene glycol must contain the other enantiomer as an impurity. The enantiomers are enriched.

The corresponding dissymmetry factor (g_{abs} factor) was calculated using the equation (1).[11]

$$g_{CD} = \frac{CD\ [mdeg]}{32980\ \times Absorbance\ [a.u.]} \quad \text{eq. (1)}$$

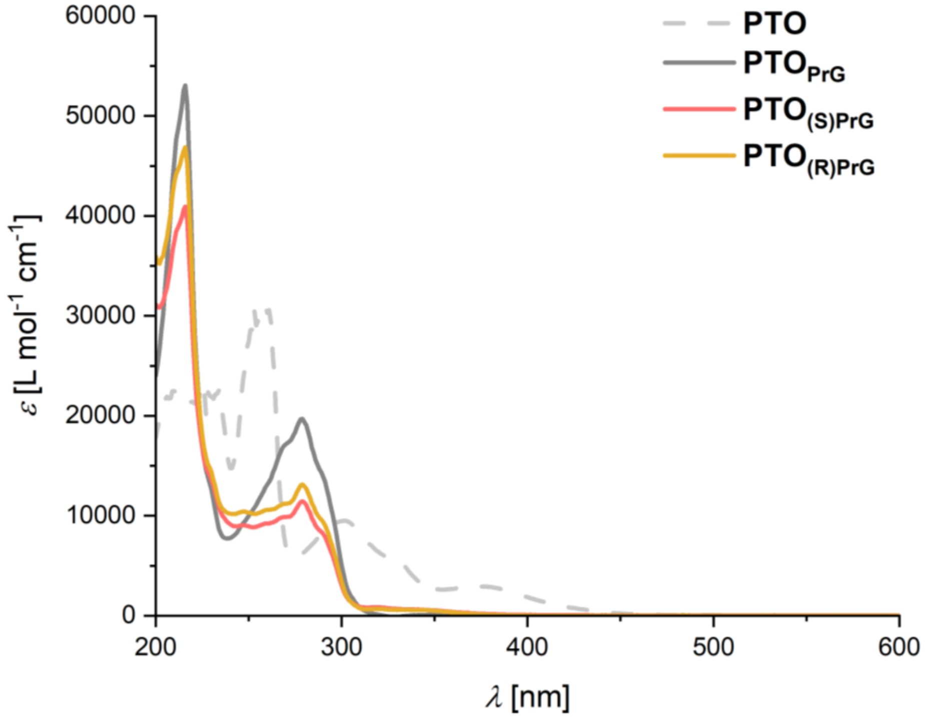

Figure S60. UV-Vis absorption spectra of **PTO$_{(S)PrG}$** (red; c = 4.6×10^{-5} M), **PTO$_{(R)PrG}$** (yellow; c = 4.6×10^{-5} M), racemic **PTO$_{PrG}$** (grey solid; c = 3.6×10^{-5} M), and precursor **PTO** (grey dashed; c = 1.1×10^{-4} M). The spectra were recorded in acetonitrile at 25 °C.

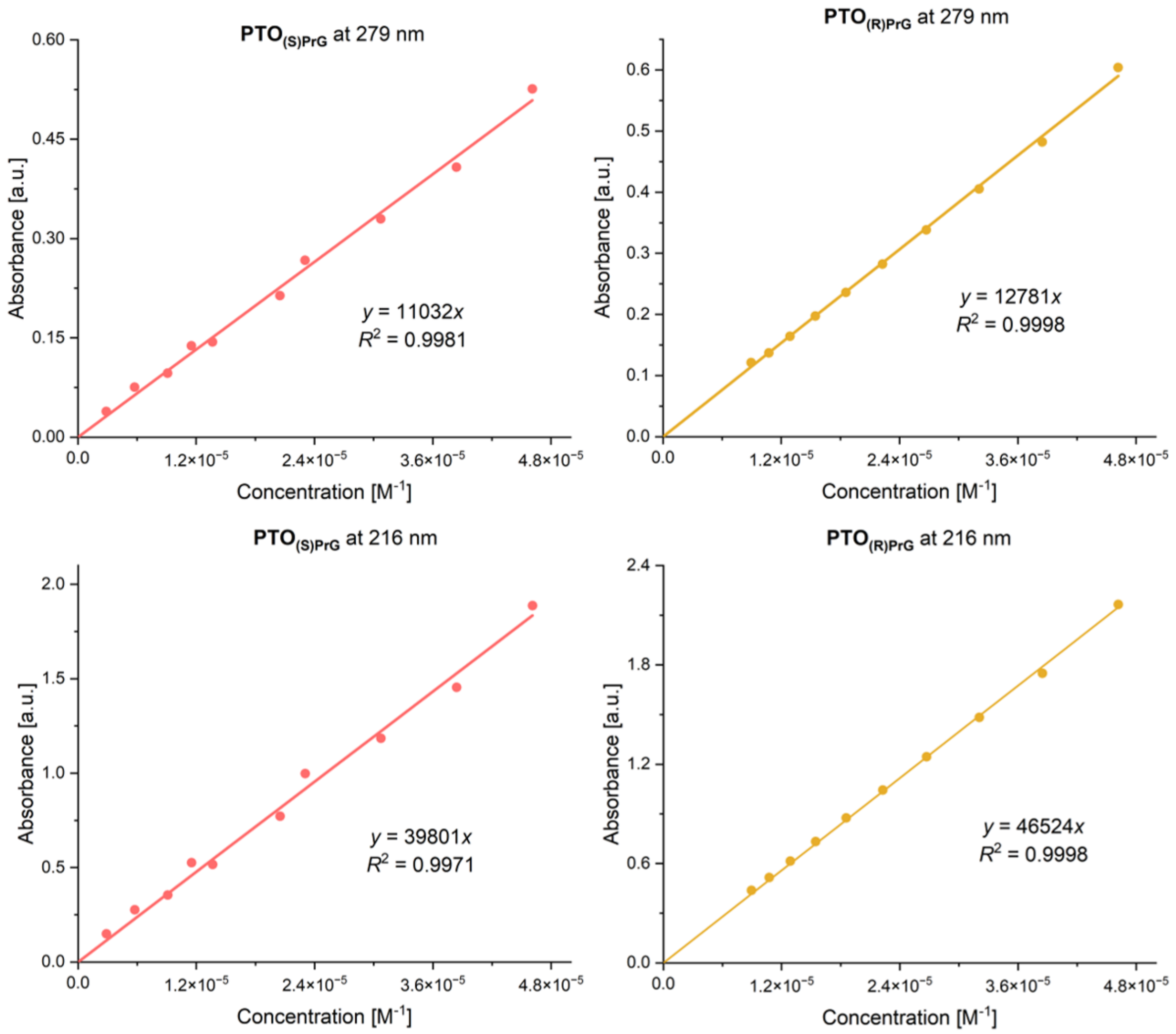

Figure S61. Beer's law plots of (*S*)-PrG- (right) and (*R*)-PrG-protected **PTO** (left) to show recorded UV-Vis spectra (**Figure S60**) at concentrations of 10^{-5} M are valid. Absorbance is taken from the absorbance maximums at 279 nm (top) and 216 nm (bottom). The spectra were recorded in acetonitrile at 298 K.

S10. Computational Methods

All calculations were conducted with Gaussian 16 Revision A.03 on the chccs-cluster of the Department of Chemistry of the Humboldt University of Berlin. The structures are confirmed ground-state minima according to the analysis of their analytical frequencies computed at the same level, which show no imaginary frequencies.

S10.1. Geometry Optimized Structures of PTO_{PrG} Isomers

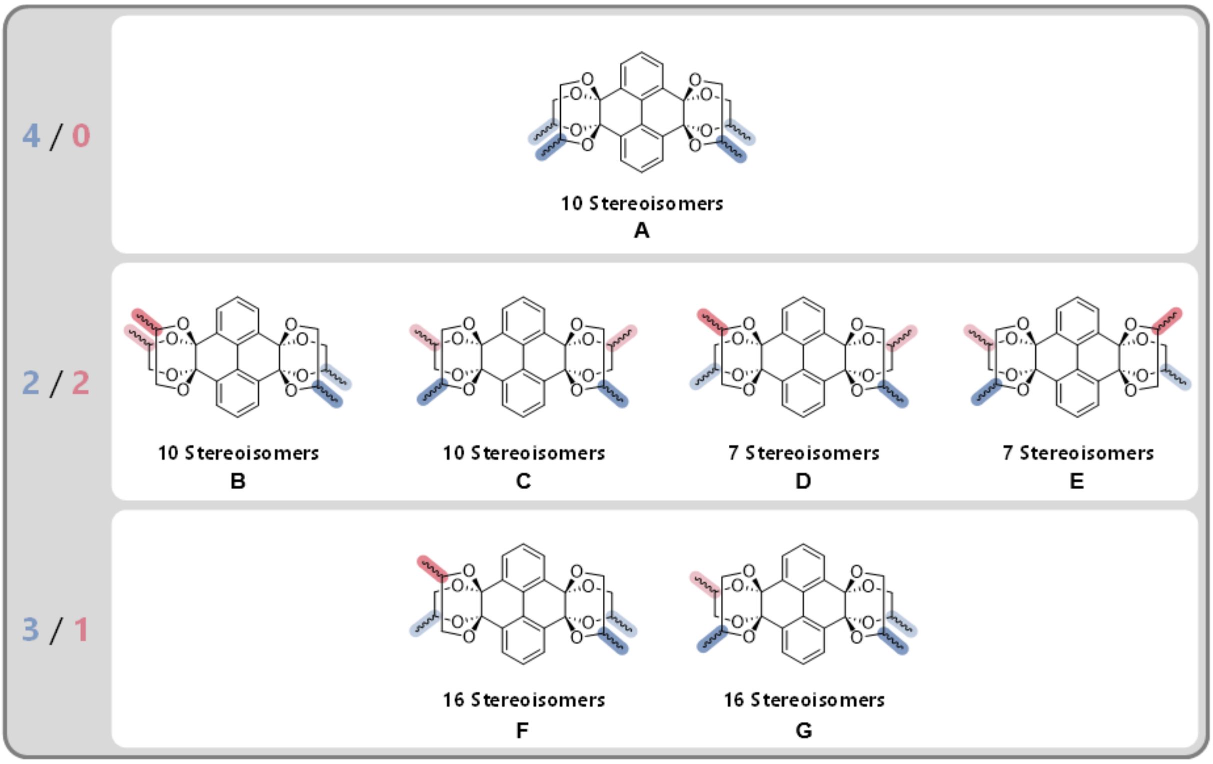

Figure S62. Overview of all possible regioisomers of compound **PTO_{PrG}** categorized by the orientation of the methyl groups pointing "downward" (blue) or "upward" (red).

Table S4. Calculated *Gibbs* free energies of the different regioisomers of **$PTO_{(S)PrG}$** at the DFT-D3-gCP/B3LYP/def2TZVP level of theory at 25 °C and 1 atm pressure.

$PTO_{(S)PrG}$ Isomer	Energy (kcal mol^{-1})	Relative Energy, rounded (kcal mol^{-1})
A	−1058952.96489	7
B	−1058957.78725	2
C	−1058958.90609	1

D	−1058971.36048	0
E	−1058971.36048	0
F	−1058954.71123	5
G	−1058954.71123	5

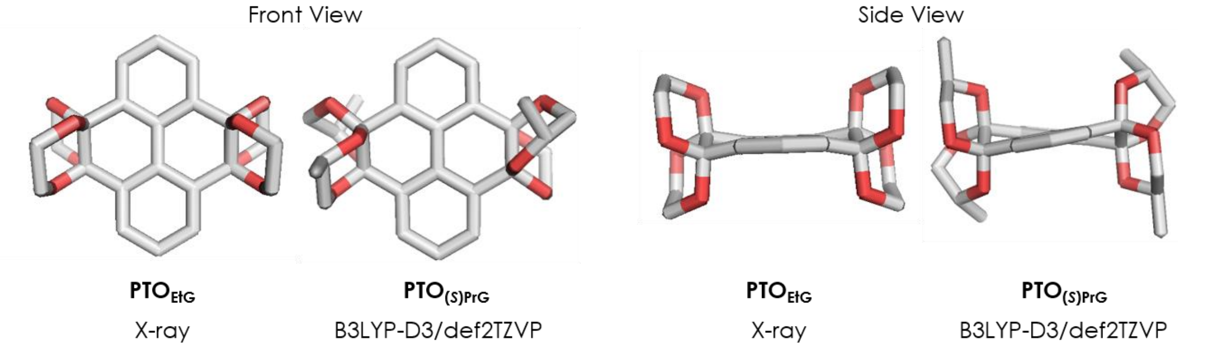

Figure S63. Comparison of K region twisting due to protecting groups of **PTO$_{EtG}$** (X-ray)[7] and **PTO$_{(S)PrG}$** (geometry-optimized (DFT) structure of isomer D/E). Color code: carbon, grey; oxygen, red. Hydrogen atoms are omitted for clarity. Optimized geometry is used for **PTO$_{(S)PrG}$** as crystallization of mixture of isomers was unsuccessful.

S10.2. Atomic Coordinates of Calculated Structures

PTO$_{(S)PrG}$ Isomer A

C	-1.14826	2.85308	-0.16573
C	-1.19708	1.45765	0.01235
C	-0.00635	0.70701	-0.06233
C	1.22407	1.35614	-0.31320
C	1.23567	2.74971	-0.51460
C	0.05955	3.48887	-0.43453
C	-0.05397	-0.77007	0.04972
C	1.14585	-1.50081	0.21493
C	-2.50466	0.75608	0.34786
C	-2.59869	-0.69563	-0.30726
C	-1.29127	-1.45165	-0.08217
C	-1.29724	-2.85982	-0.07594
C	-0.11844	-3.57663	0.09249
C	1.09101	-2.90881	0.24557
O	-2.48794	0.67447	1.76823
O	-2.81813	-0.68013	-1.71865
O	-3.61123	1.56138	-0.04845
O	-3.66027	-1.48267	0.24728
C	-4.35818	-0.88047	1.31671
C	-3.40541	-0.24526	2.33918
C	-3.91088	1.49566	-1.44483
C	-2.94014	0.61456	-2.26759
C	3.66312	0.31579	2.10017
C	3.68542	1.62163	1.29838
C	3.59299	-0.77939	-2.11958
C	4.38587	-1.27983	-0.88766
O	3.53637	-1.68686	0.16547
O	3.63732	1.35732	-0.09536
O	2.49094	-0.41059	1.80008
O	2.62387	0.22276	-1.82693
C	2.46666	-0.76878	0.42734
C	2.51396	0.55007	-0.45056
H	-2.04294	3.45759	-0.09609
H	2.15831	3.27087	-0.73617
H	0.08464	4.56120	-0.58055
H	-2.21877	-3.41381	-0.20041
H	-0.14276	-4.65877	0.10707
H	1.98291	-3.50423	0.38901
H	-4.98482	-1.64660	1.82086
H	-5.06702	-0.12386	0.93008
H	-3.30867	0.53519	-3.31242
H	-1.93460	1.07318	-2.35558
H	3.65782	0.55485	3.18497
H	4.57193	-0.29961	1.91737
H	5.03437	-2.13090	-1.18576
H	5.08078	-0.49876	-0.51566
H	-3.85663	2.52556	-1.85918
C	-5.35120	1.00984	-1.63264
H	-5.47408	-0.03693	-1.29016
H	-6.04680	1.66342	-1.06489
H	-5.63257	1.05310	-2.70643
C	-2.62766	-1.30926	3.11908
H	-1.97239	-1.90547	2.45749
H	-1.99352	-0.81907	3.88787
H	-3.32986	-1.99839	3.63485
H	-4.03491	0.30252	3.07362
H	2.82371	2.25448	1.60444
C	4.95571	2.41168	1.60422
H	4.95180	3.36372	1.03220
H	5.01110	2.65186	2.68756
H	5.85543	1.82680	1.31570
C	2.89649	-1.93868	-2.83605
H	2.13649	-2.41645	-2.19072
H	2.38998	-1.56246	-3.75008
H	3.63889	-2.70671	-3.14074
H	4.33296	-0.35409	-2.83161

PTO$_{(S)PrG}$ Isomer B

C	-1.42910	2.83335	0.09882
C	-1.34661	1.42813	0.10319
C	-0.09319	0.80633	-0.07420
C	1.06755	1.59625	-0.25045
C	0.94682	2.99817	-0.27925
C	-0.29074	3.60797	-0.09949
C	-0.00302	-0.67457	-0.12259
C	1.26053	-1.30483	-0.04406
C	-2.58040	0.57617	0.36613
C	-2.54793	-0.80287	-0.43526
C	-1.17506	-1.45475	-0.29158
C	-1.05307	-2.85454	-0.39773
C	0.18947	-3.47135	-0.30097
C	1.33630	-2.70814	-0.11556
O	-2.54388	0.35190	1.77039
O	-2.77909	-0.66031	-1.83796
O	-3.75998	1.31211	0.05421
O	-3.52963	-1.73616	0.03213
C	-4.26874	-1.31427	1.15894
C	-3.36897	-0.70188	2.24243
C	-4.06440	1.36510	-1.34139

C	-3.02451	0.66720	-2.25020
C	3.66857	0.50288	1.98463
C	3.53801	1.88886	1.34972
C	3.73157	0.14585	-2.24989
C	3.78836	-1.25087	-1.62422
O	3.68733	-1.17229	-0.21081
O	3.48401	1.79123	-0.06181
O	2.56094	-0.29196	1.61153
O	2.51642	0.77724	-1.90420
C	2.51305	-0.47097	0.20370
C	2.42181	0.93872	-0.49769
H	-2.37409	3.33823	0.25091
H	1.81327	3.62634	-0.44213
H	-0.36756	4.68759	-0.11214
H	-1.92265	-3.48033	-0.55187
H	0.26371	-4.54920	-0.36861
H	2.28579	-3.22065	-0.02907
H	-4.81766	-2.18274	1.58118
H	-5.04772	-0.59059	0.85257
H	-3.39370	0.66214	-3.29783
H	-2.06644	1.22345	-2.29008
H	2.64587	2.42329	1.74236
H	4.42541	2.49823	1.62341
H	4.60556	0.76691	-1.95042
H	3.77380	0.05177	-3.35597
C	5.10072	-1.94353	-1.98446
H	5.12087	-2.96176	-1.54118
H	5.19935	-2.03732	-3.08709
H	5.96498	-1.36678	-1.59055
H	2.96506	-1.87031	-2.04184
H	4.62411	0.02562	1.66902
C	3.70573	0.61293	3.50763
H	3.80479	-0.39903	3.95466
H	4.57367	1.22714	3.82960
H	2.77106	1.07895	3.88736
C	-2.49112	-1.76356	2.91110
H	-1.78850	-2.22579	2.19365
H	-1.89913	-1.29796	3.72730
H	-3.12318	-2.56343	3.35219
H	-4.04010	-0.29213	3.02817
H	-4.10801	2.43319	-1.64588
C	-5.45585	0.77123	-1.57999
H	-5.47953	-0.31216	-1.34829
H	-6.20324	1.29565	-0.94770
H	-5.74949	0.89970	-2.64364

PTO$_{(S)PrG}$ Isomer C

C	-1.35570	-2.84750	-0.25676
C	-1.37562	-1.43903	-0.20039
C	-0.15867	-0.74104	-0.02536
C	1.05558	-1.45416	0.10280
C	1.03250	-2.86085	0.07856
C	-0.16255	-3.54736	-0.10796
C	-0.15869	0.74100	0.02528
C	1.05555	1.45414	-0.10297
C	-2.67597	-0.67482	-0.41642
C	-2.67599	0.67479	0.41656
C	-1.37565	1.43893	0.20025
C	-1.35586	2.84749	0.25632
C	-0.16272	3.54737	0.10743
C	1.03244	2.86085	-0.07895
O	-2.70558	-0.41751	-1.81557
O	-2.70528	0.41745	1.81557
O	-3.77201	-1.53604	-0.09659
O	-3.77206	1.53595	0.09690
C	-4.43682	1.22802	-1.11958
C	-3.52586	0.66516	-2.22827
C	-4.43655	-1.22818	1.12001
C	-3.52530	-0.66538	2.22847
C	3.59891	-0.18001	-2.11381
C	3.56431	-1.57581	-1.48598
C	3.59853	0.18010	2.11397
C	3.56393	1.57595	1.48606
O	3.48275	1.48519	0.07279
O	3.48289	-1.48507	-0.07271
O	2.43314	0.53315	-1.75840
O	2.43289	-0.53308	1.75829
C	2.36162	0.70617	-0.35137
C	2.36162	-0.70611	0.35126
H	-2.26366	-3.41464	-0.41471
H	1.94224	-3.43415	0.20293
H	-0.16406	-4.62940	-0.13890
H	-2.26381	3.41462	0.41422
H	-0.16420	4.62943	0.13815
H	1.94220	3.43419	-0.20349
H	-4.95229	2.13725	-1.49504
H	-5.24206	0.49697	-0.92800
H	-5.24190	-0.49729	0.92863
H	-4.95188	-2.13747	1.49560
H	3.62462	-0.27812	-3.22001
H	4.51596	0.38000	-1.82270
H	4.51568	-0.37982	1.82297
H	3.62411	0.27835	3.22015

H	-4.20494	-0.29216	3.02573
C	-2.66362	-1.74278	2.89411
H	-1.93498	-2.19353	2.20446
H	-2.09877	-1.29499	3.73921
H	-3.30786	-2.55190	3.29909
C	-2.66434	1.74272	-2.89411
H	-1.93558	2.19348	-2.20464
H	-2.09970	1.29495	-3.73925
H	-3.30880	2.55186	-3.29891
H	-4.20562	0.29195	-3.02535
H	2.69548	2.13902	1.89234
C	4.82186	2.35854	1.85625
H	4.77608	3.37443	1.40945
H	4.90284	2.46218	2.95949
H	5.72781	1.84164	1.47292
C	4.82228	-2.35831	-1.85597
H	4.77655	-3.37421	-1.40918
H	4.90340	-2.46199	-2.95913
H	5.72813	-1.84138	-1.47246
H	2.69586	-2.13891	-1.89244

PTO$_{(S)PrG}$ Isomer D

C	-1.17760	2.91353	0.23785
C	-1.19973	1.50558	0.23330
C	-0.00003	0.79924	-0.00599
C	1.19936	1.50252	-0.25624
C	1.17626	2.91011	-0.28305
C	-0.00090	3.60567	-0.02826
C	-0.00007	-0.68142	0.00326
C	1.22556	-1.38661	0.07242
C	-2.48279	0.74659	0.56400
C	-2.54864	-0.64078	-0.19994
C	-1.22552	-1.38755	-0.05905
C	-1.20227	-2.79641	-0.06397
C	0.00035	-3.48847	0.01556
C	1.20278	-2.79540	0.08941
O	-2.44426	0.53052	1.96695
O	-2.71702	-0.40080	-1.58753
O	-3.62689	1.54821	0.26761
O	-3.59633	-1.50905	0.25361
C	-4.31672	-1.00209	1.35764
C	-3.38265	-0.42684	2.44722
C	-3.82332	1.69055	-1.13036
C	-3.90650	0.32549	-1.82406
C	3.89840	0.35071	1.82516
C	3.81699	1.70660	1.11362
C	3.39242	-0.45576	-2.43829
C	4.32239	-1.01688	-1.33767
O	3.59780	-1.51077	-0.23028
O	3.62636	1.54591	-0.28308
O	2.71050	-0.37936	1.59293
O	2.45081	0.50626	-1.97390
C	2.54780	-0.63754	0.20783
C	2.48384	0.73996	-0.57374
C	4.07034	0.54669	3.33021
H	-2.07335	3.48430	0.44576
H	2.07181	3.47808	-0.49965
H	-0.00122	4.68808	-0.03671
H	-2.11528	-3.37302	-0.13355
H	0.00049	-4.57093	0.02020
H	2.11587	-3.37126	0.16422
H	-4.93849	-1.81277	1.79334
H	-5.03808	-0.23634	1.00456
H	-4.77607	2.23725	-1.29441
H	-3.01712	2.30890	-1.57990
H	4.78786	-0.21744	1.47105
H	3.00862	2.33021	1.55167
H	4.76869	2.25621	1.27446
H	4.94647	-1.83252	-1.76071
H	5.04174	-0.24645	-0.99098
H	3.20657	1.10465	3.75146
H	5.00395	1.11012	3.54242
H	4.13320	-0.44186	3.83273
C	2.63414	-1.58147	-3.14634
H	1.95918	-2.11595	-2.45212
H	2.02215	-1.15846	-3.97091
H	3.34746	-2.31296	-3.58224
H	4.04243	0.02881	-3.19859
H	-4.79402	-0.23872	-1.45904
C	-4.08493	0.50190	-3.33084
H	-4.14936	-0.49310	-3.82028
H	-5.02001	1.06198	-3.54620
H	-3.22353	1.05507	-3.76291
C	-2.61986	-1.54310	3.16549
H	-1.94754	-2.08536	2.47477
H	-2.00460	-1.10932	3.98199
H	-3.33044	-2.26981	3.61355
H	-4.02977	0.06627	3.20455

PTO$_{(S)PrG}$ Isomer E

C	-1.17760	2.91353	0.23785
C	-1.19973	1.50558	0.23330
C	-0.00003	0.79924	-0.00599

C	1.19936	1.50252	-0.25624
C	1.17626	2.91011	-0.28305
C	-0.00090	3.60567	-0.02826
C	-0.00007	-0.68142	0.00326
C	1.22556	-1.38661	0.07242
C	-2.48279	0.74659	0.56400
C	-2.54864	-0.64078	-0.19994
C	-1.22552	-1.38755	-0.05905
C	-1.20227	-2.79641	-0.06397
C	0.00035	-3.48847	0.01556
C	1.20278	-2.79540	0.08941
O	-2.44426	0.53052	1.96695
O	-2.71702	-0.40080	-1.58753
O	-3.62689	1.54821	0.26761
O	-3.59633	-1.50905	0.25361
C	-4.31672	-1.00209	1.35764
C	-3.38265	-0.42684	2.44722
C	-3.82332	1.69055	-1.13036
C	-3.90650	0.32549	-1.82406
C	3.89840	0.35071	1.82516
C	3.81699	1.70660	1.11362
C	3.39242	-0.45576	-2.43829
C	4.32239	-1.01688	-1.33767
O	3.59780	-1.51077	-0.23028
O	3.62636	1.54591	-0.28308
O	2.71050	-0.37936	1.59293
O	2.45081	0.50626	-1.97390
C	2.54780	-0.63754	0.20783
C	2.48384	0.73996	-0.57374
C	4.07034	0.54669	3.33021
H	-2.07335	3.48430	0.44576
H	2.07181	3.47808	-0.49965
H	-0.00122	4.68808	-0.03671
H	-2.11528	-3.37302	-0.13355
H	0.00049	-4.57093	0.02020
H	2.11587	-3.37126	0.16422
H	-4.93849	-1.81277	1.79334
H	-5.03808	-0.23634	1.00456
H	-4.77607	2.23725	-1.29441
H	-3.01712	2.30890	-1.57990
H	4.78786	-0.21744	1.47105
H	3.00862	2.33021	1.55167
H	4.76869	2.25621	1.27446
H	4.94647	-1.83252	-1.76071
H	5.04174	-0.24645	-0.99098
H	3.20657	1.10465	3.75146
H	5.00395	1.11012	3.54242
H	4.13320	-0.44186	3.83273
C	2.63414	-1.58147	-3.14634
H	1.95918	-2.11595	-2.45212
H	2.02215	-1.15846	-3.97091
H	3.34746	-2.31296	-3.58224
H	4.04243	0.02881	-3.19859
H	-4.79402	-0.23872	-1.45904
C	-4.08493	0.50190	-3.33084
H	-4.14936	-0.49310	-3.82028
H	-5.02001	1.06198	-3.54620
H	-3.22353	1.05507	-3.76291
C	-2.61986	-1.54310	3.16549
H	-1.94754	-2.08536	2.47477
H	-2.00460	-1.10932	3.98199
H	-3.33044	-2.26981	3.61355
H	-4.02977	0.06627	3.20455

PTO$_{(S)PrG}$ Isomer F

C	-1.23934	2.80405	-0.12675
C	-1.22820	1.40377	0.01769
C	-0.00615	0.70539	-0.06788
C	1.19981	1.41805	-0.27899
C	1.14759	2.81328	-0.45923
C	-0.06024	3.49763	-0.37605
C	-0.00502	-0.77980	-0.05947
C	1.21668	-1.48173	0.03168
C	-2.50392	0.63544	0.32643
C	-2.55615	-0.77643	-0.41270
C	-1.21755	-1.49319	-0.25517
C	-1.17105	-2.89547	-0.38046
C	0.03387	-3.58129	-0.28150
C	1.21806	-2.88574	-0.06920
O	-2.45542	0.46762	1.73896
O	-2.80652	-0.68753	-1.81601
O	-3.64757	1.42022	0.00046
O	-3.57415	-1.63659	0.11501
C	-4.27384	-1.12690	1.22996
C	-3.32481	-0.51923	2.27082
C	-3.97413	1.42415	-1.39166
C	-2.98737	0.63188	-2.28340
C	3.69270	0.09884	2.11915
C	3.71086	1.51417	1.56128
C	4.02330	-1.43014	-1.41341
O	3.62607	-1.53378	-0.05243
O	3.58487	1.51609	0.14179
O	2.50802	-0.54557	1.71555

O	2.79115	0.64158	-1.80689
C	2.50555	-0.72426	0.30761
C	2.53815	0.70149	-0.40392
C	2.68844	2.41986	2.26463
H	-2.15904	3.36855	-0.04985
H	2.04997	3.37799	-0.65714
H	-0.08131	4.57273	-0.49976
H	-2.07274	-3.46842	-0.55600
H	0.04973	-4.66016	-0.36850
H	2.13501	-3.45371	0.01618
H	-4.86125	-1.94553	1.69751
H	-5.01771	-0.37614	0.90235
H	-3.37348	0.60092	-3.32454
H	-2.00139	1.13287	-2.36039
H	3.74107	0.12305	3.22872
H	4.59695	-0.46419	1.79770
H	4.70830	1.94177	1.80165
H	2.75047	3.45039	1.85661
H	2.91636	2.46780	3.35086
H	1.65286	2.03982	2.16884
C	3.02411	-0.65872	-2.29827
H	2.05923	-1.19957	-2.39179
H	3.43121	-0.58540	-3.32905
H	-3.96858	2.47777	-1.74547
C	-5.39836	0.89365	-1.58112
H	-6.10654	1.48469	-0.96268
H	-5.70355	0.98867	-2.64511
H	-5.47392	-0.17544	-1.29955
C	-2.49066	-1.59835	2.96758
H	-1.82927	-2.12820	2.25693
H	-1.85750	-1.13192	3.75183
H	-3.15506	-2.34370	3.45428
H	-3.95801	-0.04156	3.04963
H	4.09692	-2.45845	-1.82893
C	5.41943	-0.80491	-1.47813
H	5.41795	0.22779	-1.07576
H	6.12949	-1.41364	-0.87925
H	5.78153	-0.77801	-2.52788

PTO$_{(S)PrG}$ Isomer G

C	-1.23934	2.80405	-0.12675
C	-1.22820	1.40377	0.01769
C	-0.00615	0.70539	-0.06788
C	1.19981	1.41805	-0.27899
C	1.14759	2.81328	-0.45923
C	-0.06024	3.49763	-0.37605
C	-0.00502	-0.77980	-0.05947
C	1.21668	-1.48173	0.03168
C	-2.50392	0.63544	0.32643
C	-2.55615	-0.77643	-0.41270
C	-1.21755	-1.49319	-0.25517
C	-1.17105	-2.89547	-0.38046
C	0.03387	-3.58129	-0.28150
C	1.21806	-2.88574	-0.06920
O	-2.45542	0.46762	1.73896
O	-2.80652	-0.68753	-1.81601
O	-3.64757	1.42022	0.00046
O	-3.57415	-1.63659	0.11501
C	-4.27384	-1.12690	1.22996
C	-3.32481	-0.51923	2.27082
C	-3.97413	1.42415	-1.39166
C	-2.98737	0.63188	-2.28340
C	3.69270	0.09884	2.11915
C	3.71086	1.51417	1.56128
C	4.02330	-1.43014	-1.41341
O	3.62607	-1.53378	-0.05243
O	3.58487	1.51609	0.14179
O	2.50802	-0.54557	1.71555
O	2.79115	0.64158	-1.80689
C	2.50555	-0.72426	0.30761
C	2.53815	0.70149	-0.40392
C	2.68844	2.41986	2.26463
H	-2.15904	3.36855	-0.04985
H	2.04997	3.37799	-0.65714
H	-0.08131	4.57273	-0.49976
H	-2.07274	-3.46842	-0.55600
H	0.04973	-4.66016	-0.36850
H	2.13501	-3.45371	0.01618
H	-4.86125	-1.94553	1.69751
H	-5.01771	-0.37614	0.90235
H	-3.37348	0.60092	-3.32454
H	-2.00139	1.13287	-2.36039
H	3.74107	0.12305	3.22872
H	4.59695	-0.46419	1.79770
H	4.70830	1.94177	1.80165
H	2.75047	3.45039	1.85661
H	2.91636	2.46780	3.35086
H	1.65286	2.03982	2.16884
C	3.02411	-0.65872	-2.29827
H	2.05923	-1.19957	-2.39179
H	3.43121	-0.58540	-3.32905
H	-3.96858	2.47777	-1.74547
C	-5.39836	0.89365	-1.58112

H	-6.10654	1.48469	-0.96268	C	5.41943	-0.80491	-1.47813
H	-5.70355	0.98867	-2.64511	H	5.41795	0.22779	-1.07576
H	-5.47392	-0.17544	-1.29955	H	6.12949	-1.41364	-0.87925
C	-2.49066	-1.59835	2.96758	H	5.78153	-0.77801	-2.52788
H	-1.82927	-2.12820	2.25693				
H	-1.85750	-1.13192	3.75183				
H	-3.15506	-2.34370	3.45428				
H	-3.95801	-0.04156	3.04963				
H	4.09692	-2.45845	-1.82893				

S11. References

[1] O. V. Dolomanov, L. J. Bourhis, R. J. Gildea, J. a. K. Howard, H. Puschmann, "OLEX2: a complete structure solution, refinement and analysis program" *J. Appl. Cryst.* **2009**, *42*, 339–341.

[2] G. M. Sheldrick, "SHELXT – Integrated space-group and crystal-structure determination" *Acta Cryst.* **2015**, *A71*, 3–8.

[3] G. M. Sheldrick, "A short history of SHELX" *Acta Cryst. A* **2008**, *A64*, 112–122.

[4] J. Hu, D. Zhang, F. W. Harris, "Ruthenium(III) Chloride Catalyzed Oxidation of Pyrene and 2,7-Disubstitued Pyrenes: An Efficient, One-Step Synthesis of Pyrene-4,5-diones and Pyrene-4,5,9,10-tetraones" *J. Org. Chem.* **2005**, *70*, 707–708.

[5] V. A. Kuehl, J. Yin, P. H. H. Duong, B. Mastorovich, B. Newell, K. D. Li-Oakey, B. A. Parkinson, J. O. Hoberg, "A Highly Ordered Nanoporous, Two-Dimensional Covalent Organic Framework with Modifiable Pores, and Its Application in Water Purification and Ion Sieving" *J. Am. Chem. Soc.* **2018**, *140*, 18200–18207.

[6] J. Merz, M. Dietz, Y. Vonhausen, F. Wöber, A. Friedrich, D. Sieh, I. Krummenacher, H. Braunschweig, M. Moos, M. Holzapfel, C. Lambert, T. B. Marder, "Synthesis, Photophysical and Electronic Properties of New Red-to-NIR Emitting Donor–Acceptor Pyrene Derivatives" *Chem. – Eur. J.* **2020**, *26*, 438–453.

[7] N. Grabicki, K. T. D. Nguyen, S. Weidner, O. Dumele, "Confined Spaces in [n]Cyclo-2,7-pyrenylenes" *Angew. Chem. Int. Ed.* **2021**, *60*, 14909–14914.

[8] C.-J. Yao, Z. Wu, J. Xie, F. Yu, W. Guo, Z. J. Xu, D.-S. Li, S. Zhang, Q. Zhang, "Two-Dimensional (2D) Covalent Organic Framework as Efficient Cathode for Binder-free Lithium-Ion Battery" *ChemSusChem* **2020**, *13*, 2457–2463.

[9] P. Grice, S. V. Ley, J. Pietruszka, H. W. M. Priepke, S. L. Warriner, "Preparation, structure, derivatisation and NMR data ofcyclohexane-1,2-diacetal protected carbohydrates" *J. Chem. Soc. Perkin 1* **1997**, 351–364.

[10] B. Kohl, M. V. Bohnwagner, F. Rominger, H. Wadepohl, A. Dreuw, M. Mastalerz, "Attractive Dispersion Interactions Versus Steric Repulsion of tert-Butyl groups in the Crystal Packing of a D3-Symmetric Tris(quinoxalinophenanthrophenazine)" *Chem. – Eur. J.* **2016**, *22*, 646–655.

[11] A. Ishii, T. Miyasaka, "Direct detection of circular polarized light in helical 1D perovskite-based photodiode" *Sci. Adv.* **2020**, *6*, eabd3274.

S12. Author Contributions

R.W., O.D., and B.E. devised the project. R.W and K.C. carried out most of the synthesis and characterization with support from P.W.. K.C. carried out the deprotection experiments, UV-Vis spectroscopy, ECD spectroscopy, and theoretical calculations. R.W. and K.C. carried out single-crystal growth. C.D. carried out the X-ray crystallography. R.W., K.C., and B.E. wrote the manuscript, with input from other authors.
